# Supplementary figures and images for: LRG1 is an adipokine that promotes insulin sensitivity and suppresses inflammation
Source: eLife. 2022 Nov 8;11:e81559. doi: 10.7554/eLife.81559 (PMC9674348; doi:10.7554/eLife.81559)

Figure 1—source data 1

Figure 1C

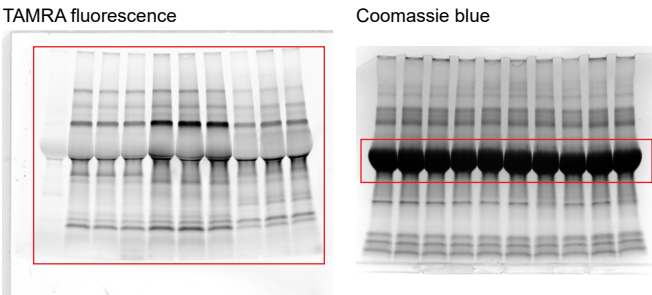

Supplement: Figure 1—source data 1. [file elife-81559-fig1-data1.pdf]

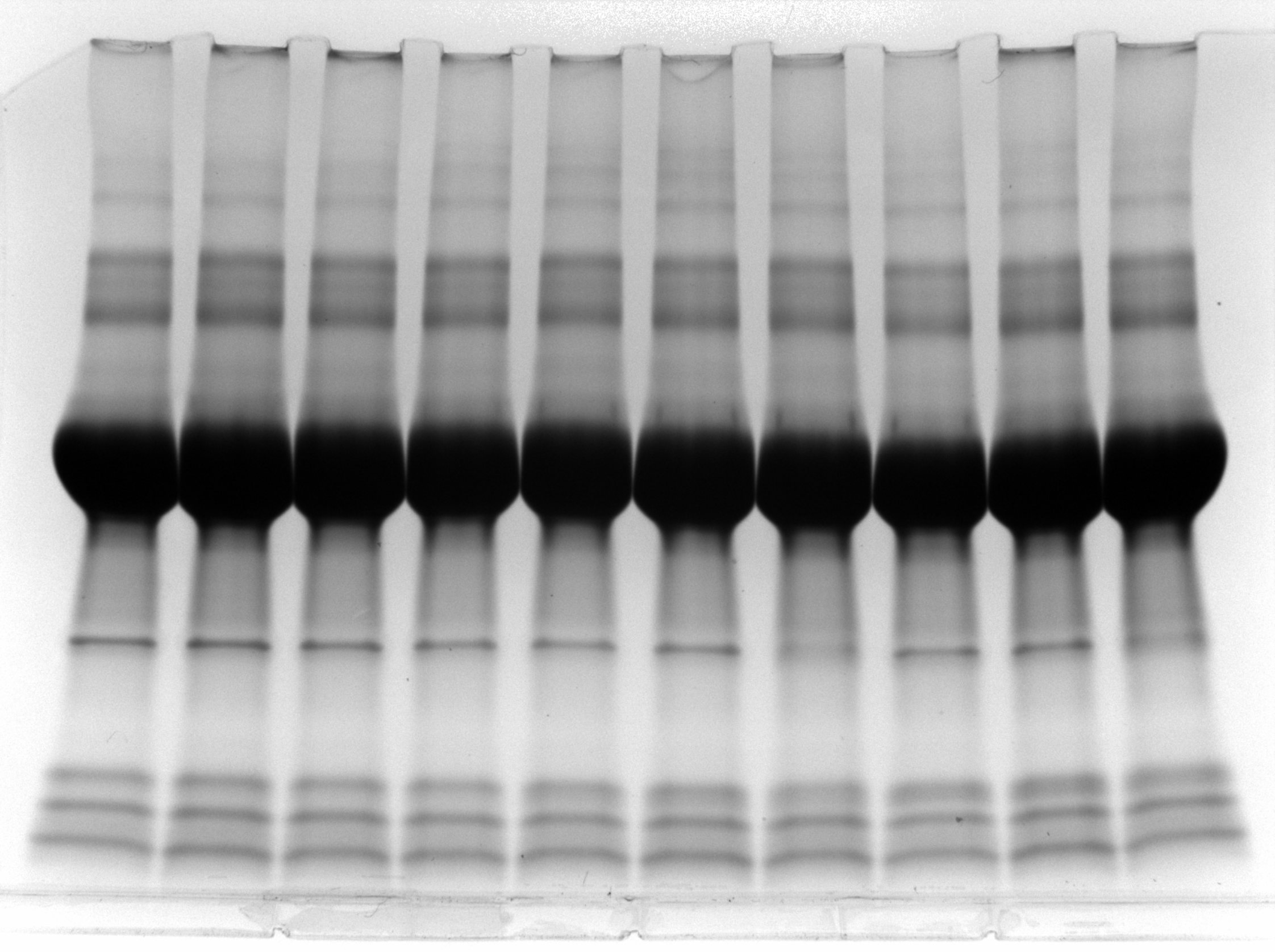

Supplement: Figure 1—source data 2. [file elife-81559-fig1-data2.zip › Figure 1-source data 2/Primary Adipocytes CM Coomassie.tif]

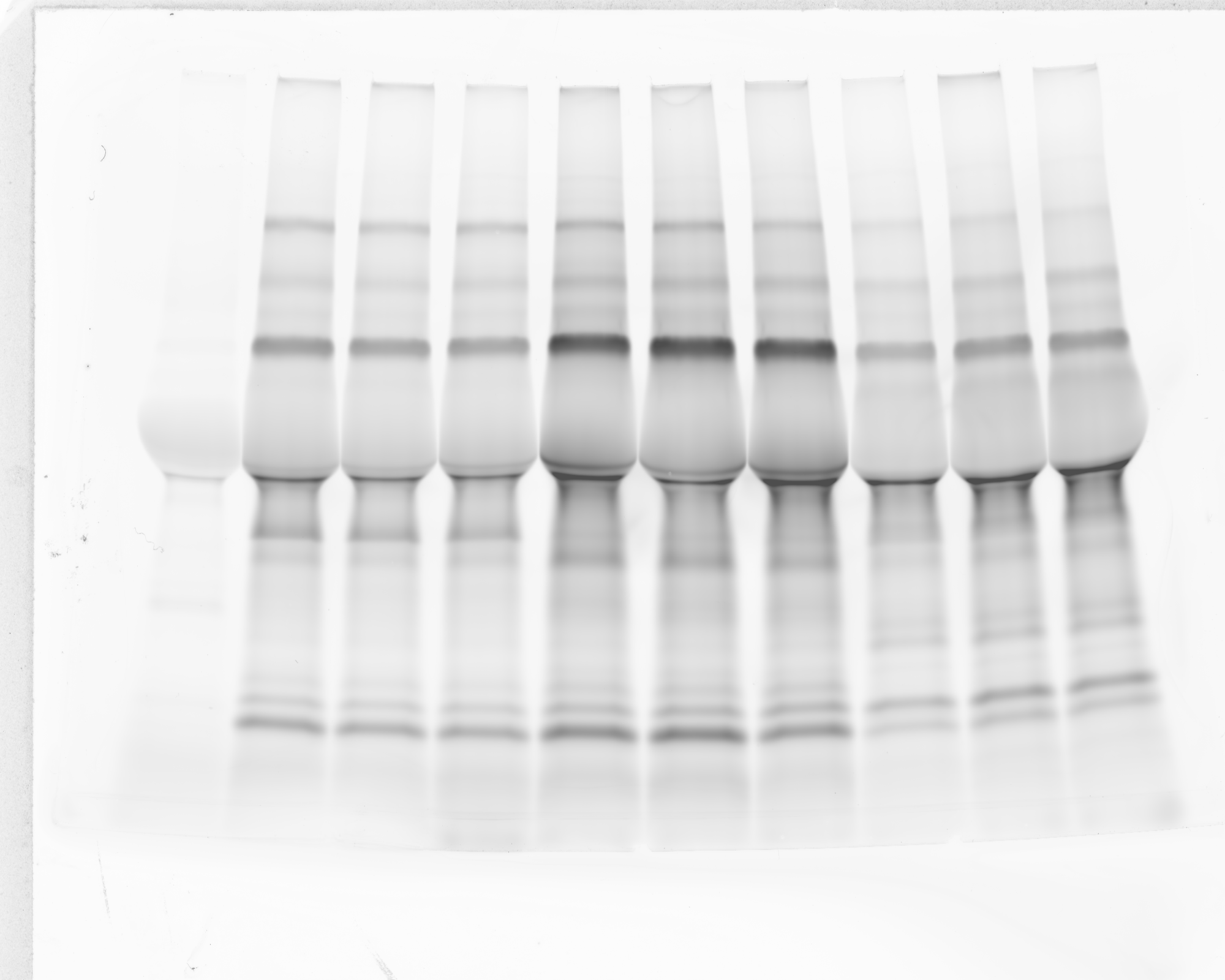

Supplement: Figure 1—source data 2. [file elife-81559-fig1-data2.zip › Figure 1-source data 2/Primary Adipocytes CM TAMRA.tif]

Figure 2—source data 1

Figure 2C

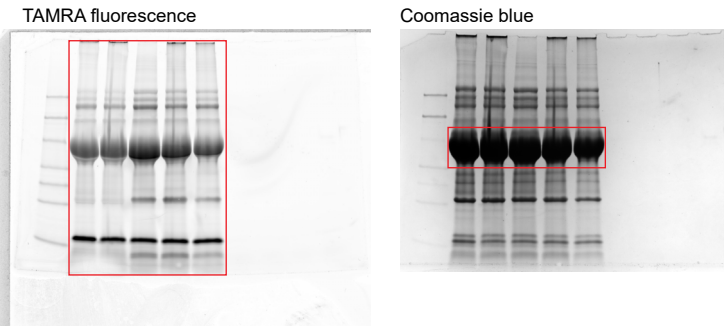

Supplement: Figure 2—source data 1. [file elife-81559-fig2-data1.pdf]

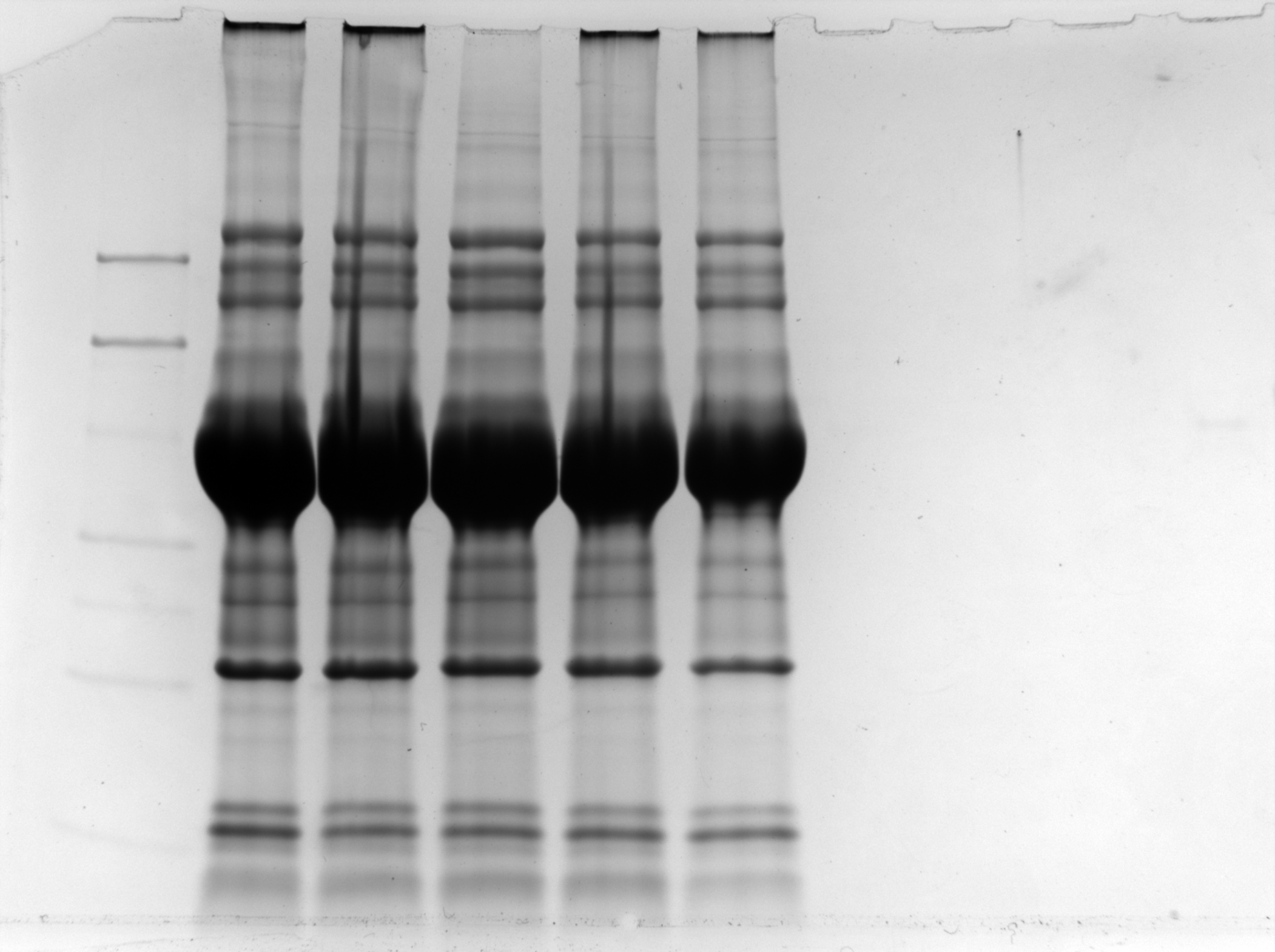

Supplement: Figure 2—source data 2. [file elife-81559-fig2-data2.zip › Figure 2-source data 2/B6 Mice AHA Injection Coomassie.tif]

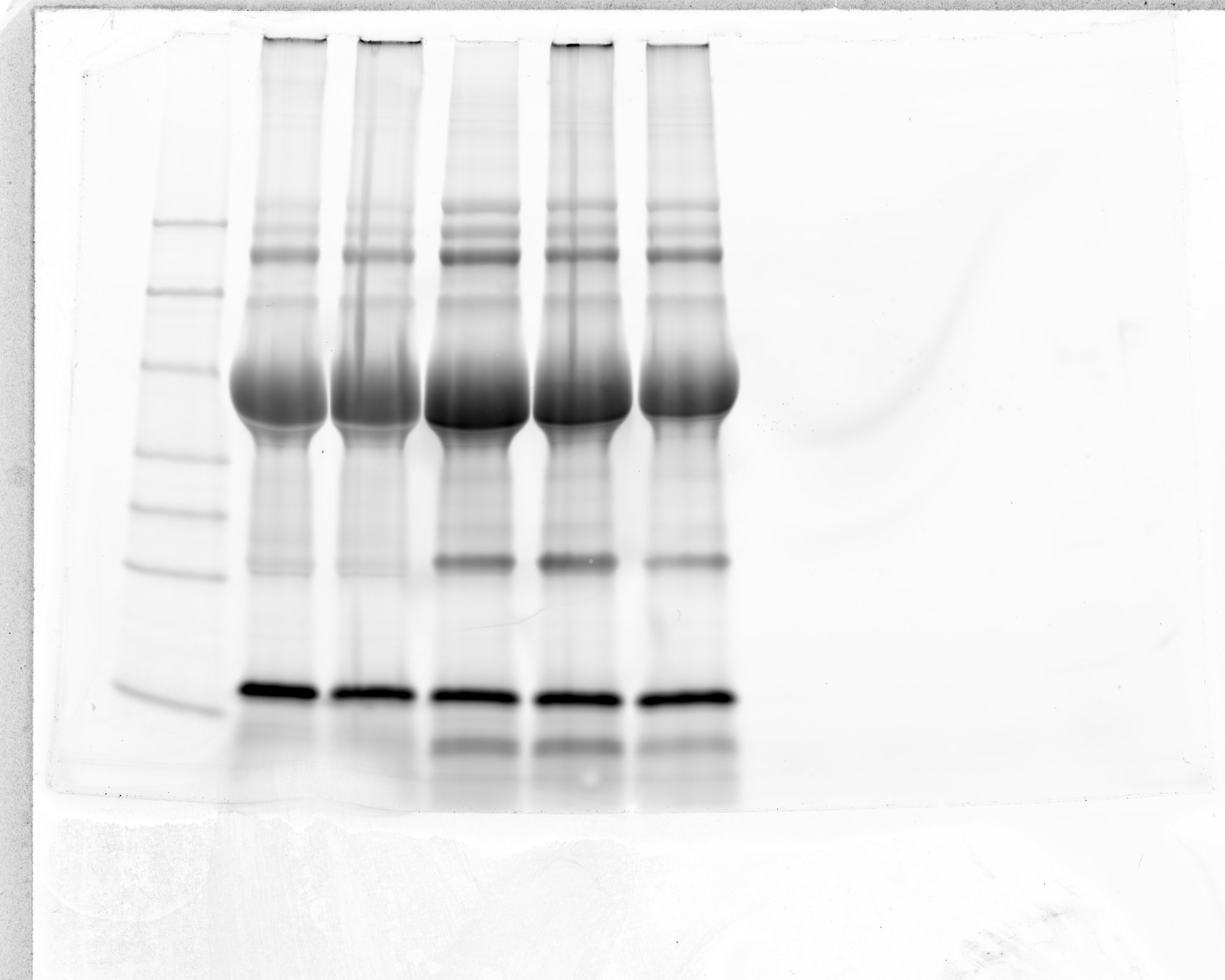

Supplement: Figure 2—source data 2. [file elife-81559-fig2-data2.zip › Figure 2-source data 2/B6 mice AHA injection TAMRA.jpg]

Figure 3—source data 1

Figure 3E  
LRG1

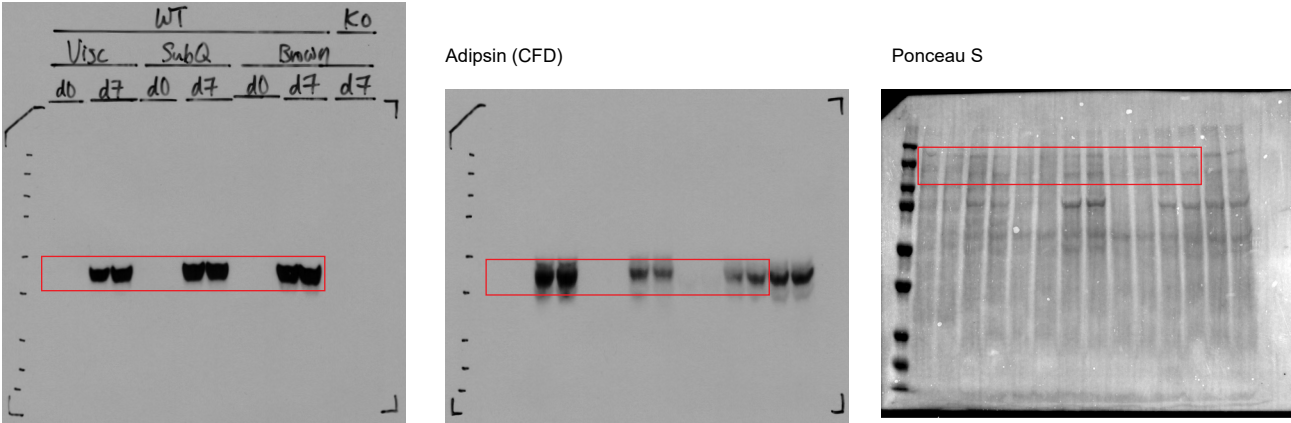

Figures 3F, 7C

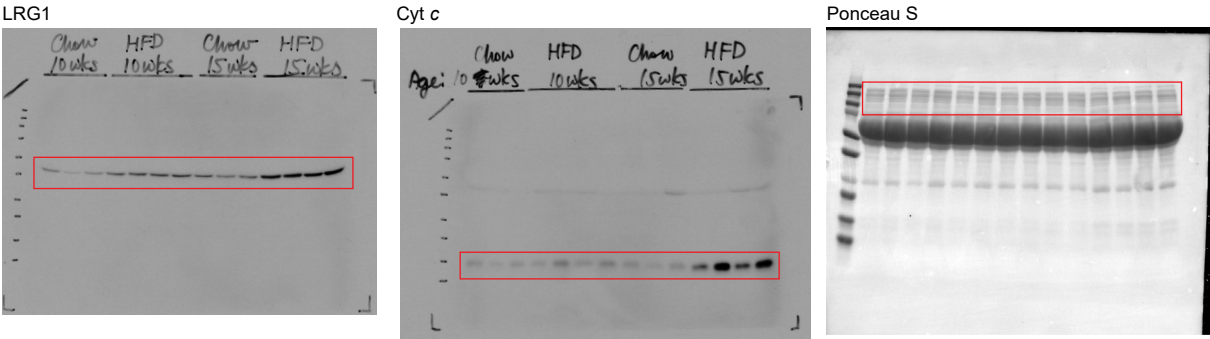

Figures 3I, 7D

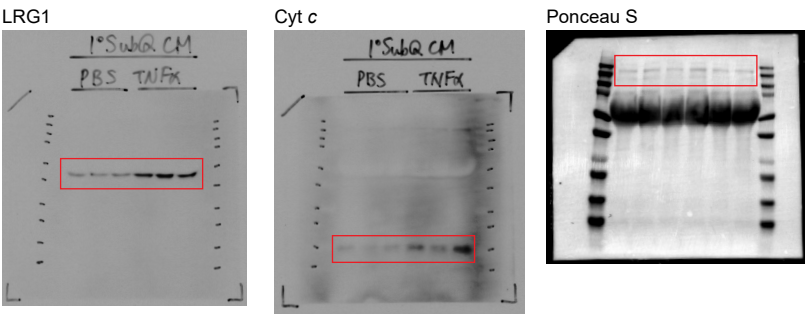

Figure 3—figure supplement 2A

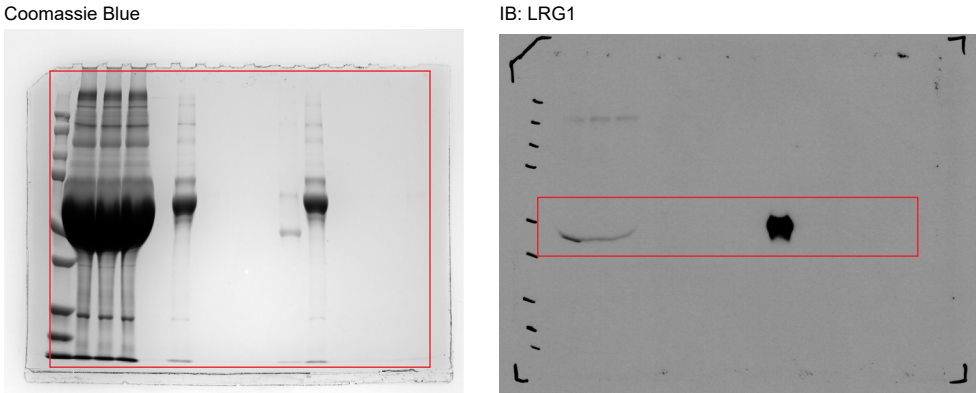

Supplement: Figure 3—source data 1. [file elife-81559-fig3-data1.pdf]

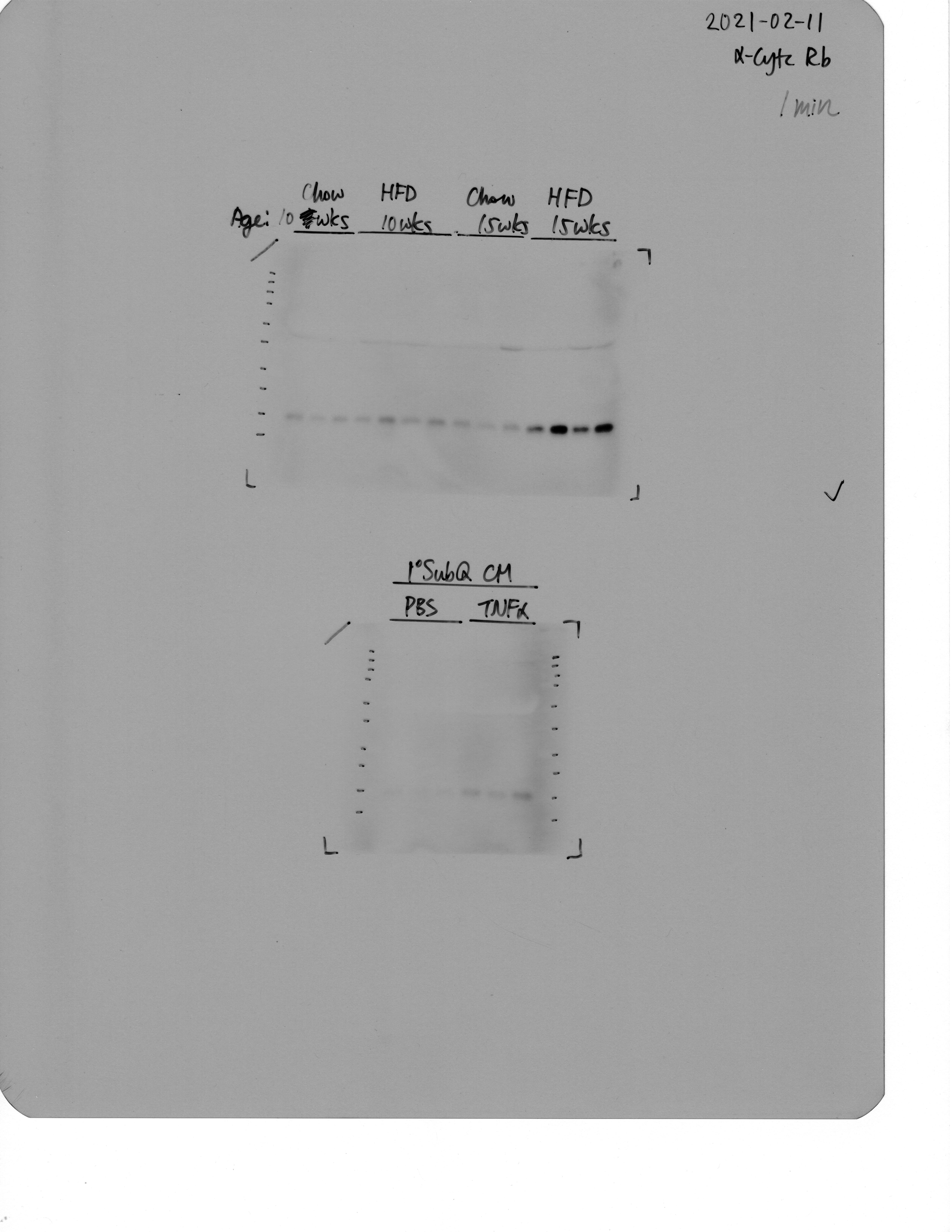

Supplement: Figure 3—source data 2. [file elife-81559-fig3-data2.zip › Figure 3-source data 2/Chow vs HFD Serum a-Cyt c 1min.jpg]

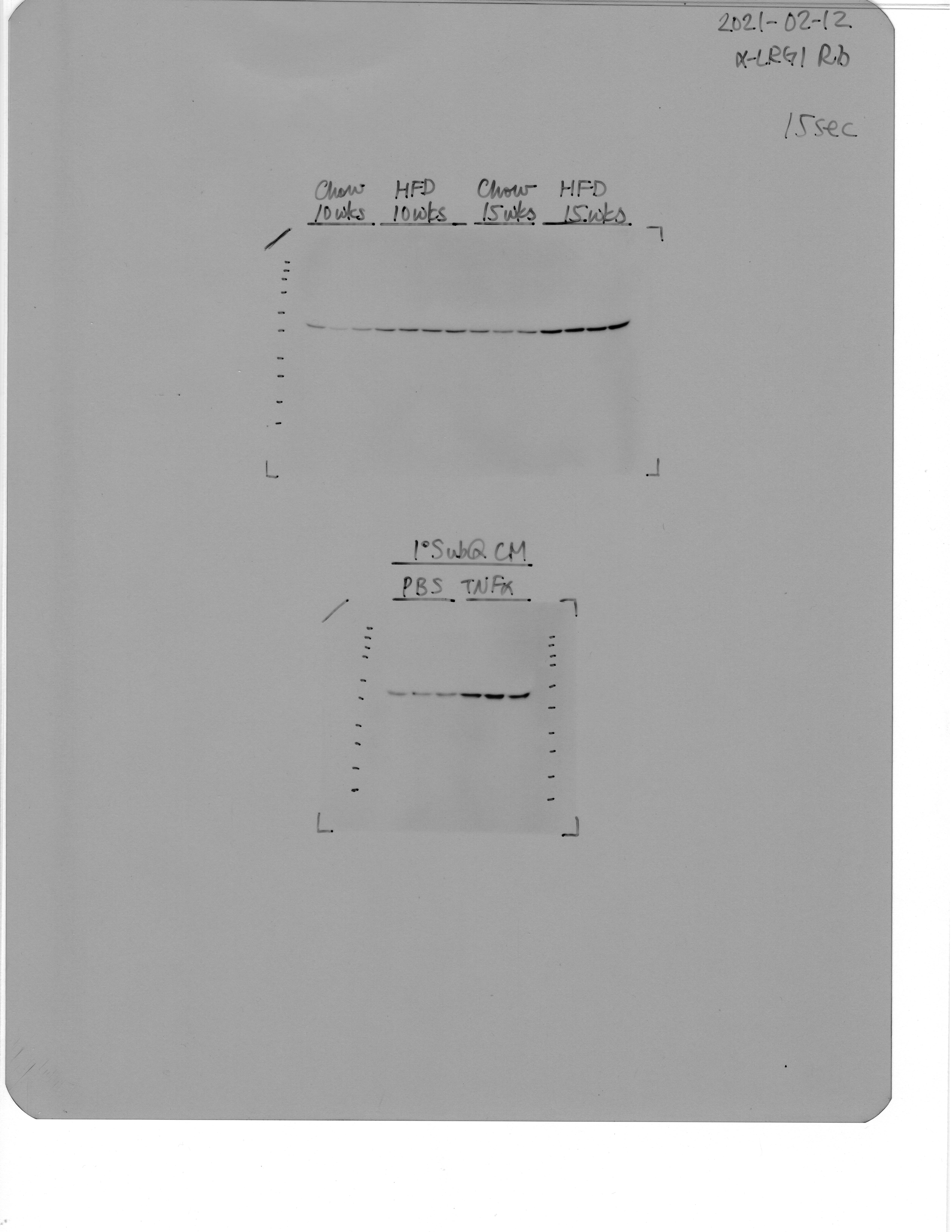

Supplement: Figure 3—source data 2. [file elife-81559-fig3-data2.zip › Figure 3-source data 2/Chow vs HFD Serum and SubQ CM TNFa a-LRG1 15sec.jpg]

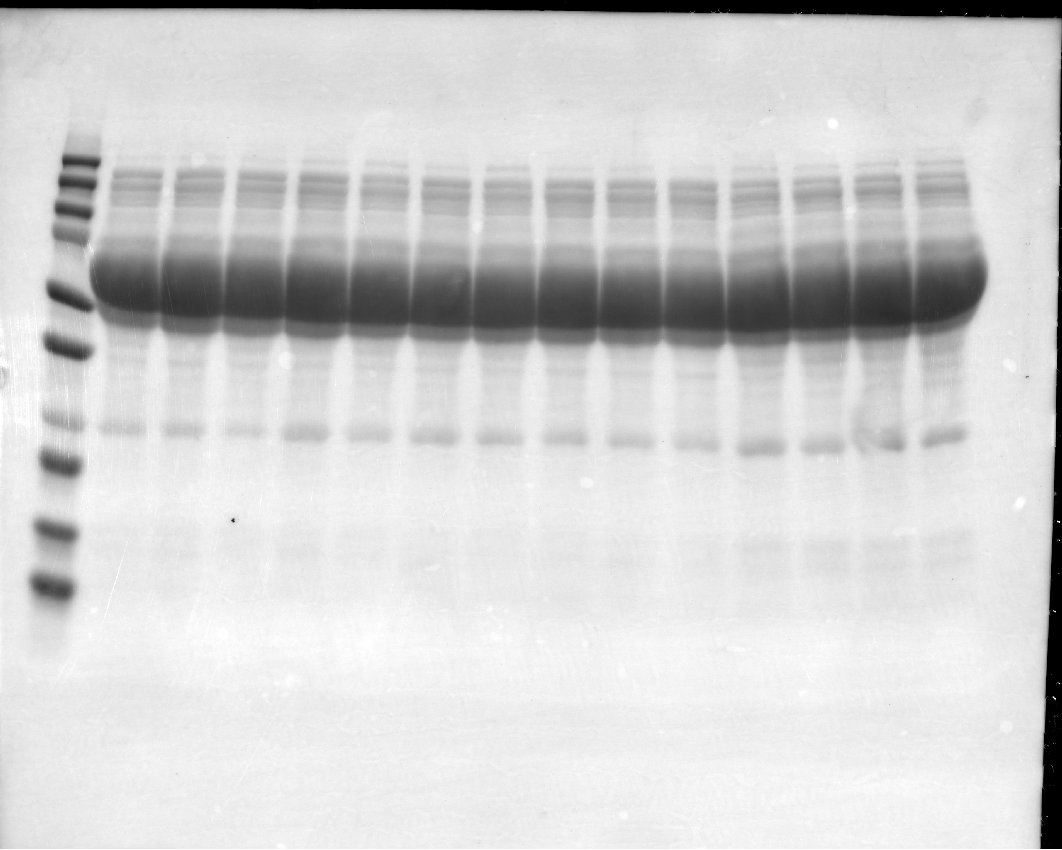

Supplement: Figure 3—source data 2. [file elife-81559-fig3-data2.zip › Figure 3-source data 2/Chow vs HFD Serum Ponceau S.tif]

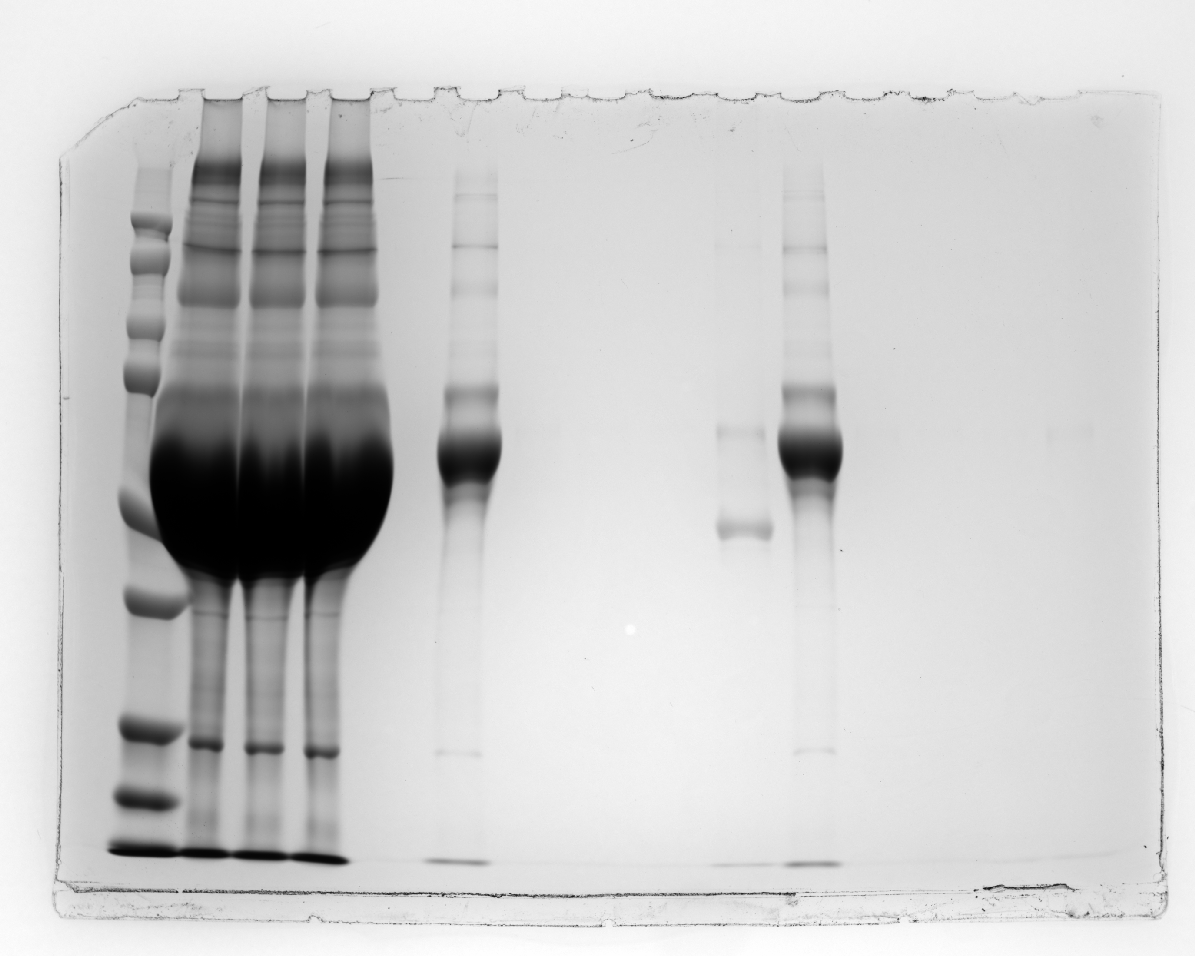

Supplement: Figure 3—source data 2. [file elife-81559-fig3-data2.zip › Figure 3-source data 2/Figure 3-S2/FBS Cyt c-agarose depletion Coomassie.tif]

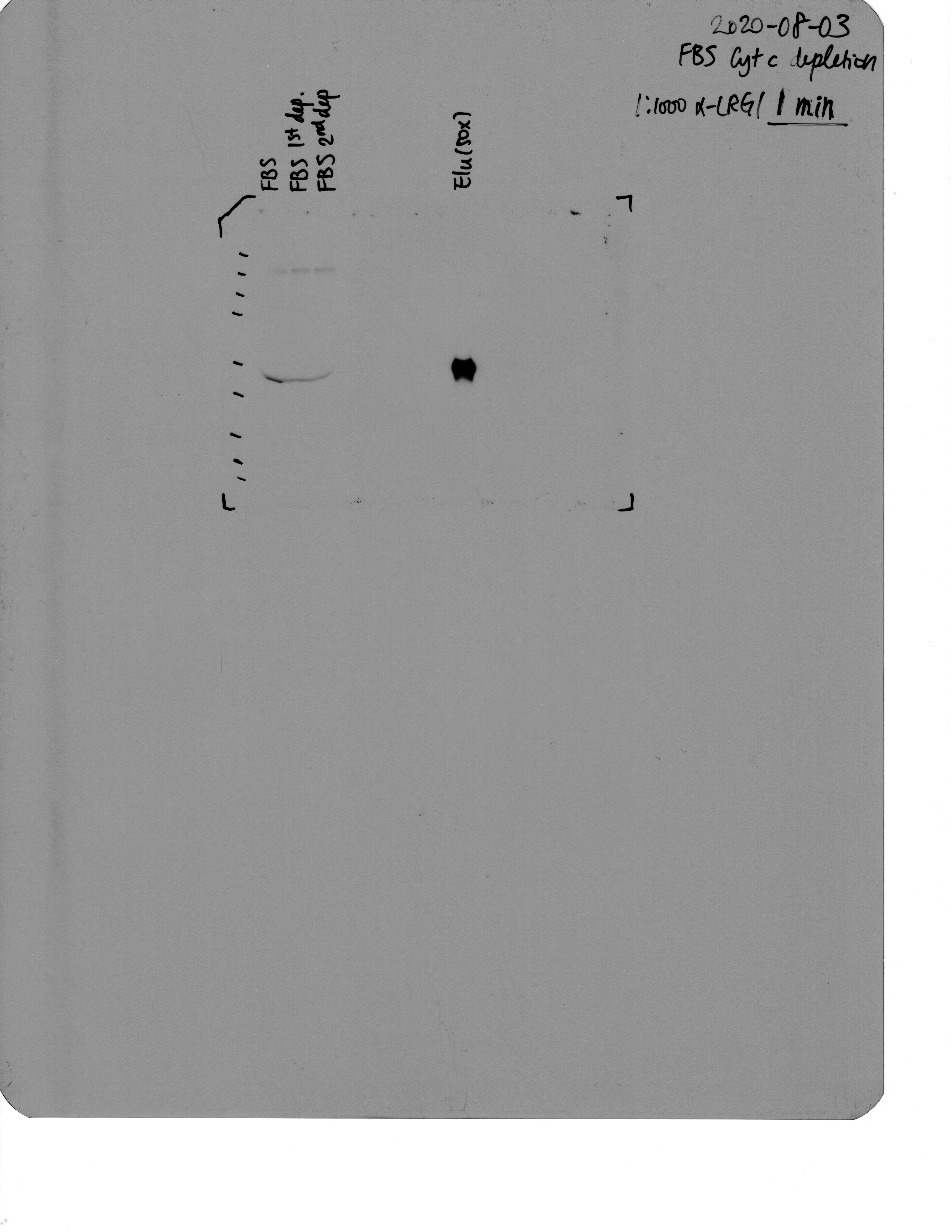

Supplement: Figure 3—source data 2. [file elife-81559-fig3-data2.zip › Figure 3-source data 2/Figure 3-S2/FBS Cytc-agarose depletion a-LRG1 1min.jpg]

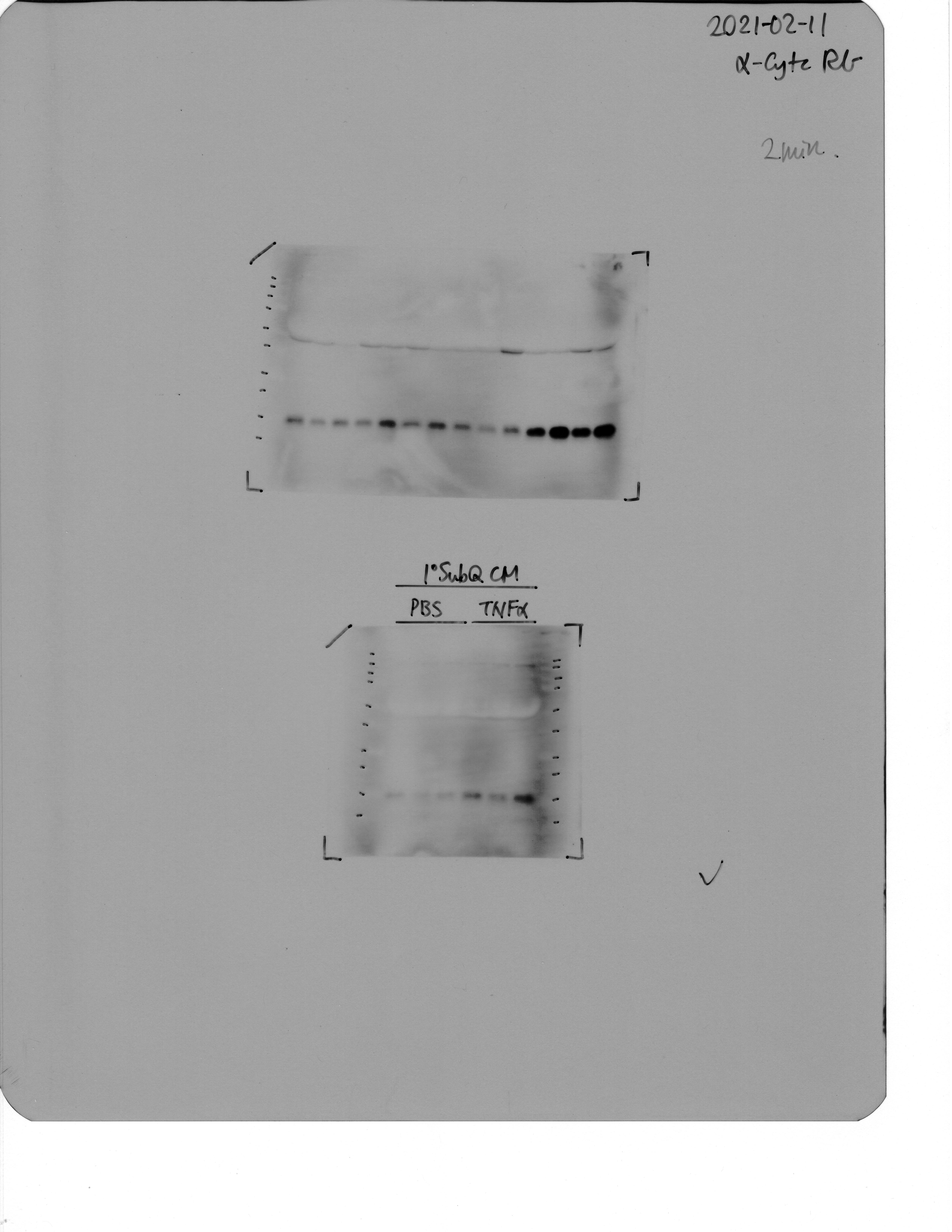

Supplement: Figure 3—source data 2. [file elife-81559-fig3-data2.zip › Figure 3-source data 2/SubQ CM TNFa a-Cyt c 2min.jpg]

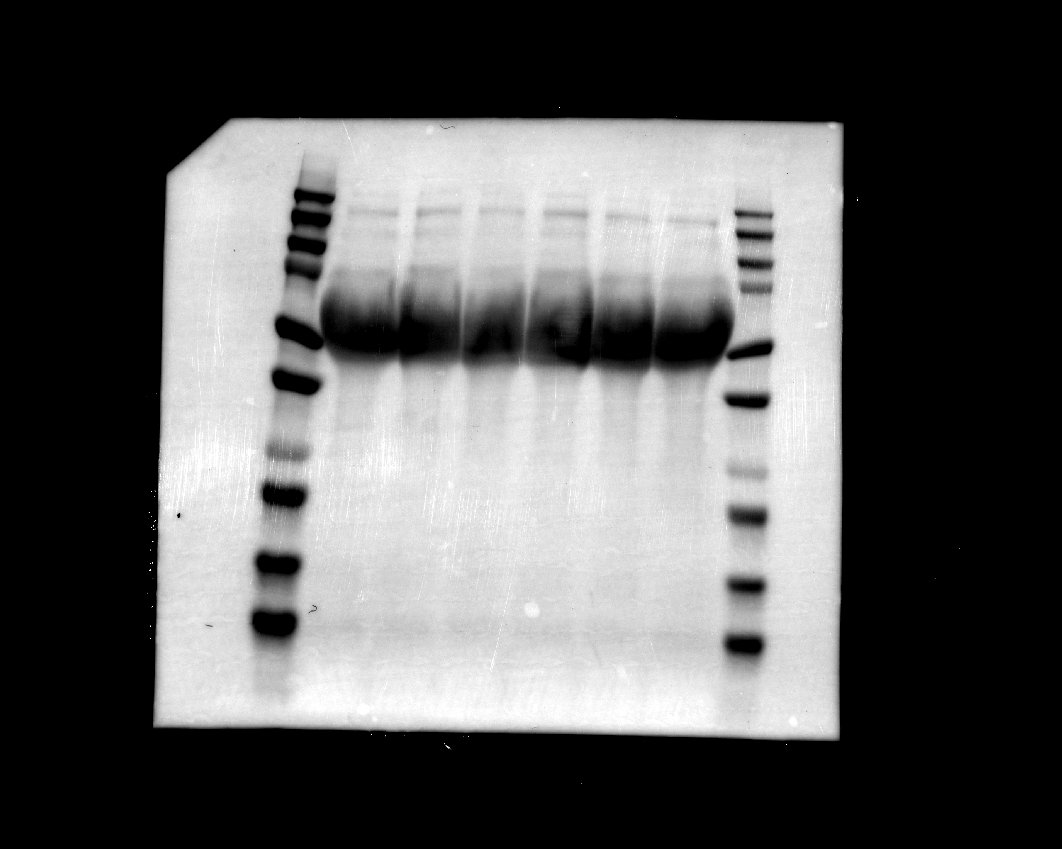

Supplement: Figure 3—source data 2. [file elife-81559-fig3-data2.zip › Figure 3-source data 2/SubQ CM TNFa Ponceau S(Ponceau S) Adj.jpg]

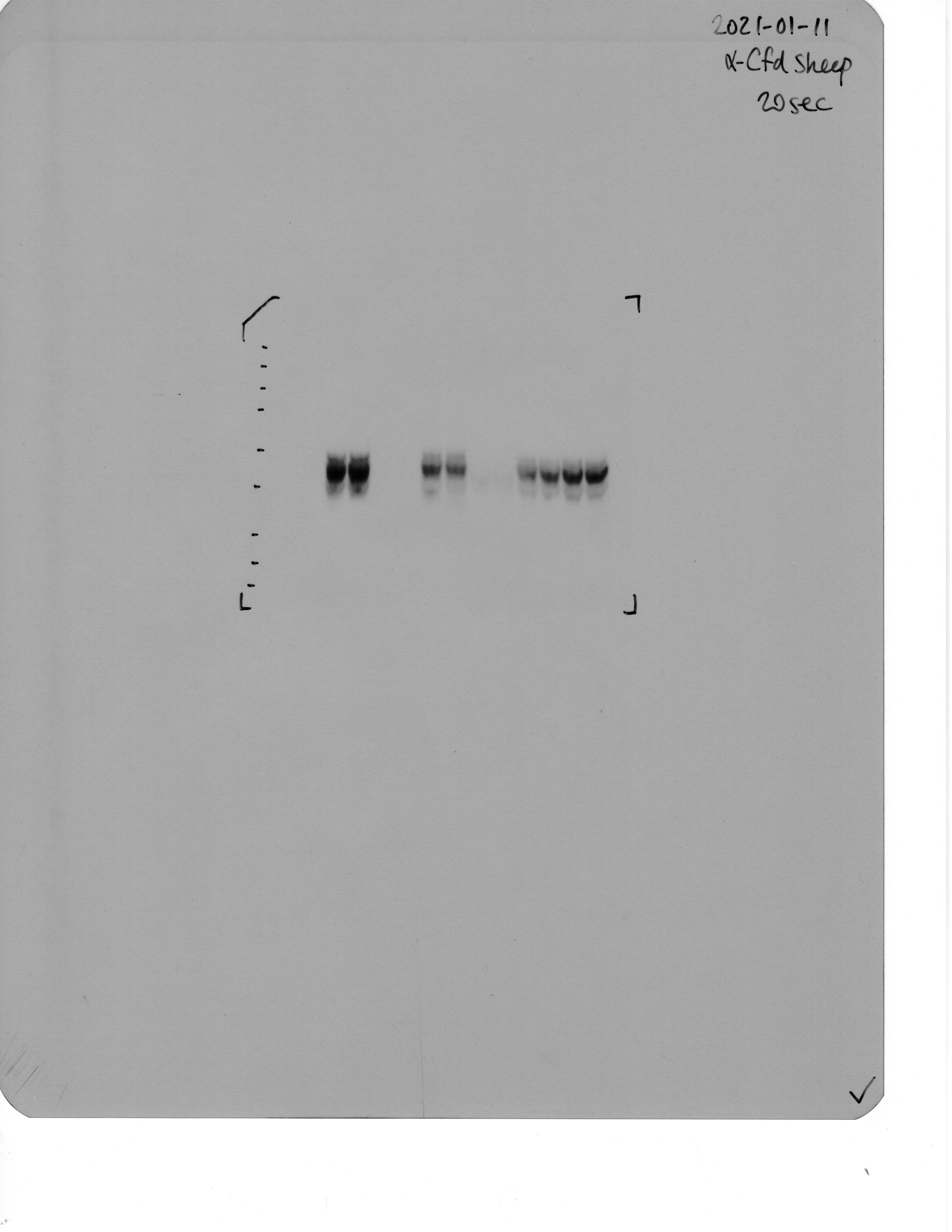

Supplement: Figure 3—source data 2. [file elife-81559-fig3-data2.zip › Figure 3-source data 2/WT and LRG1-KO CM d0-d7 a-Cfd 20sec.jpg]

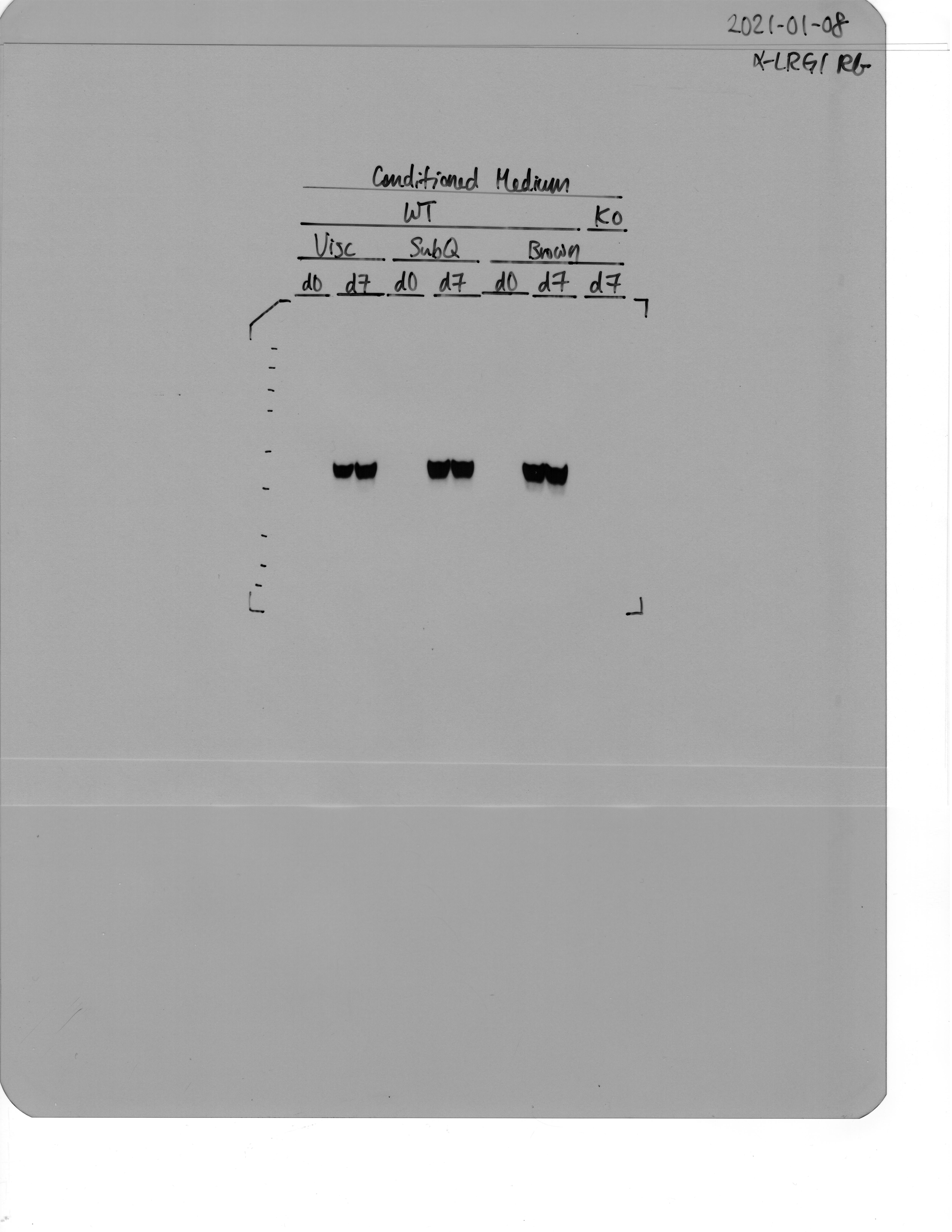

Supplement: Figure 3—source data 2. [file elife-81559-fig3-data2.zip › Figure 3-source data 2/WT and LRG1-KO CM d0-d7 a-LRG1 1sec.jpg]

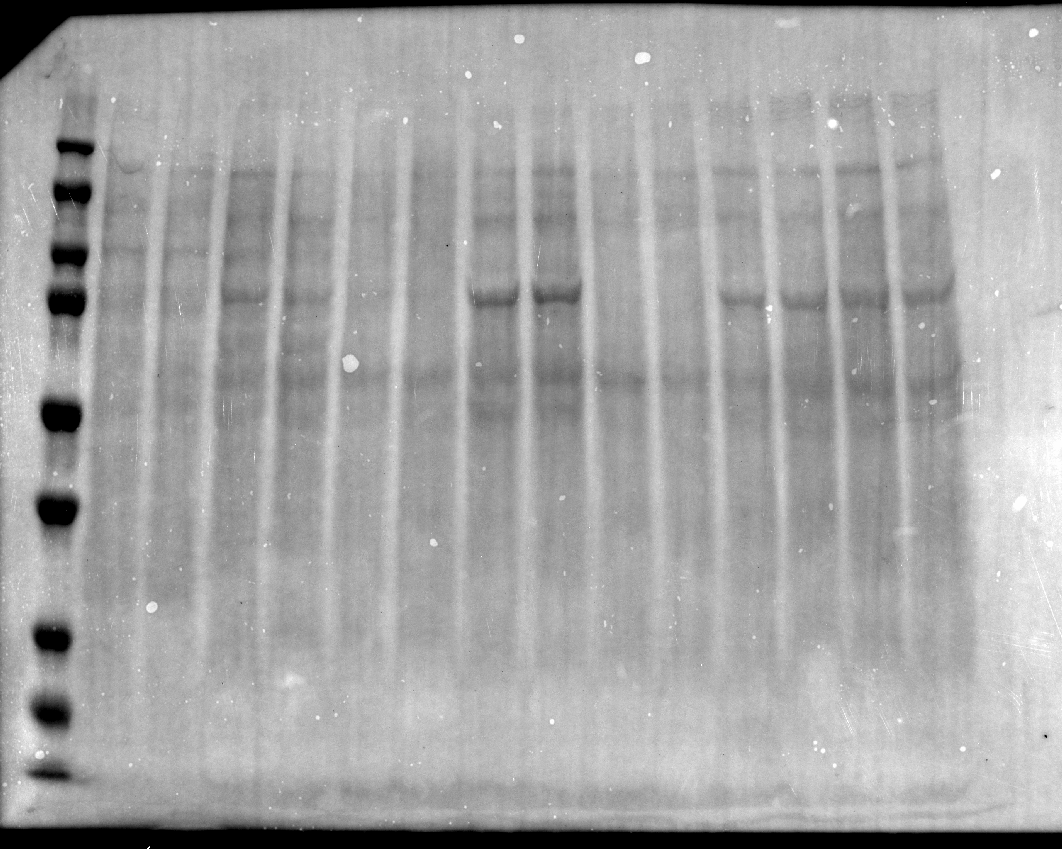

Supplement: Figure 3—source data 2. [file elife-81559-fig3-data2.zip › Figure 3-source data 2/WT and LRG1-KO CM d0-d7 Ponceau S.tif]

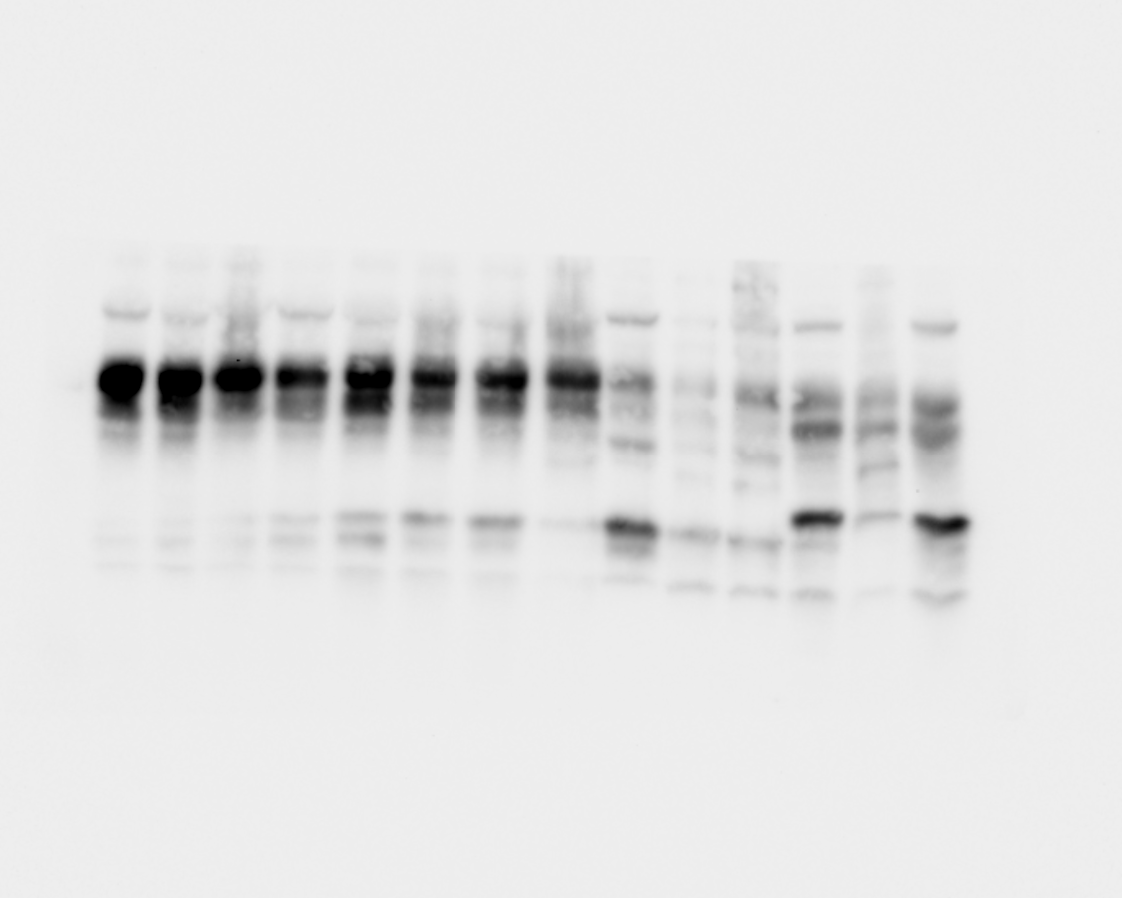

Supplement: Figure 4—source data 2. [file elife-81559-fig4-data2.zip › Figure 4-source data 2/AAV Tissues a-LRG1.tif]

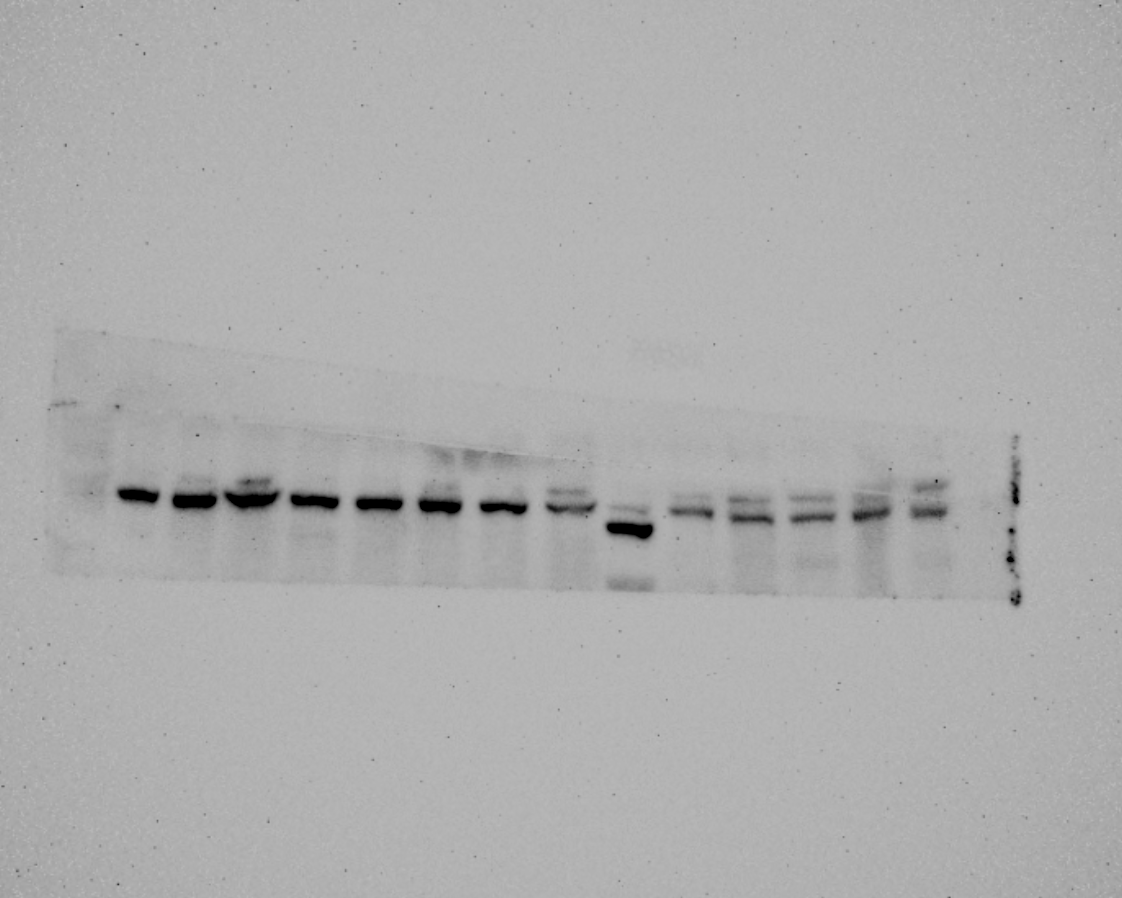

Supplement: Figure 4—source data 2. [file elife-81559-fig4-data2.zip › Figure 4-source data 2/AAV Tissues a-Vinculin.tif]

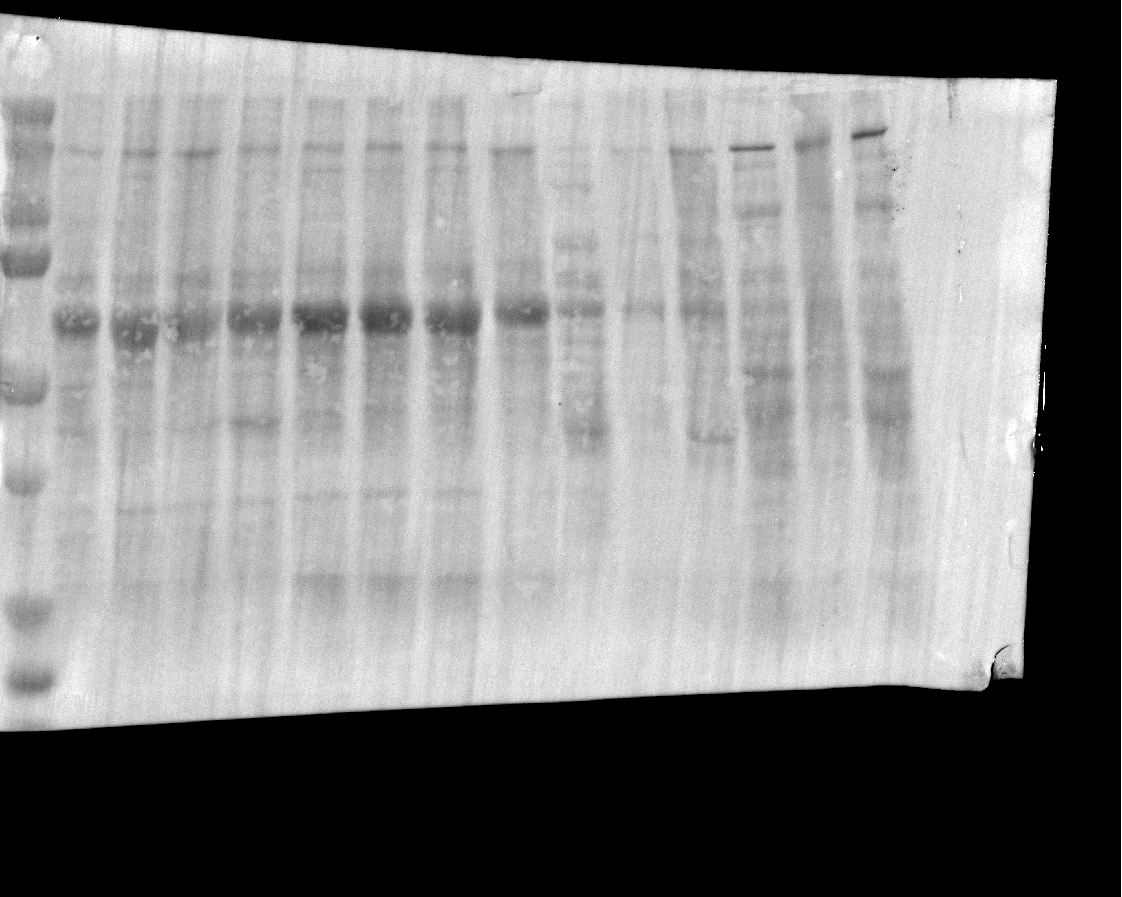

Supplement: Figure 4—source data 2. [file elife-81559-fig4-data2.zip › Figure 4-source data 2/AAV Tissues Ponceau S.tif]

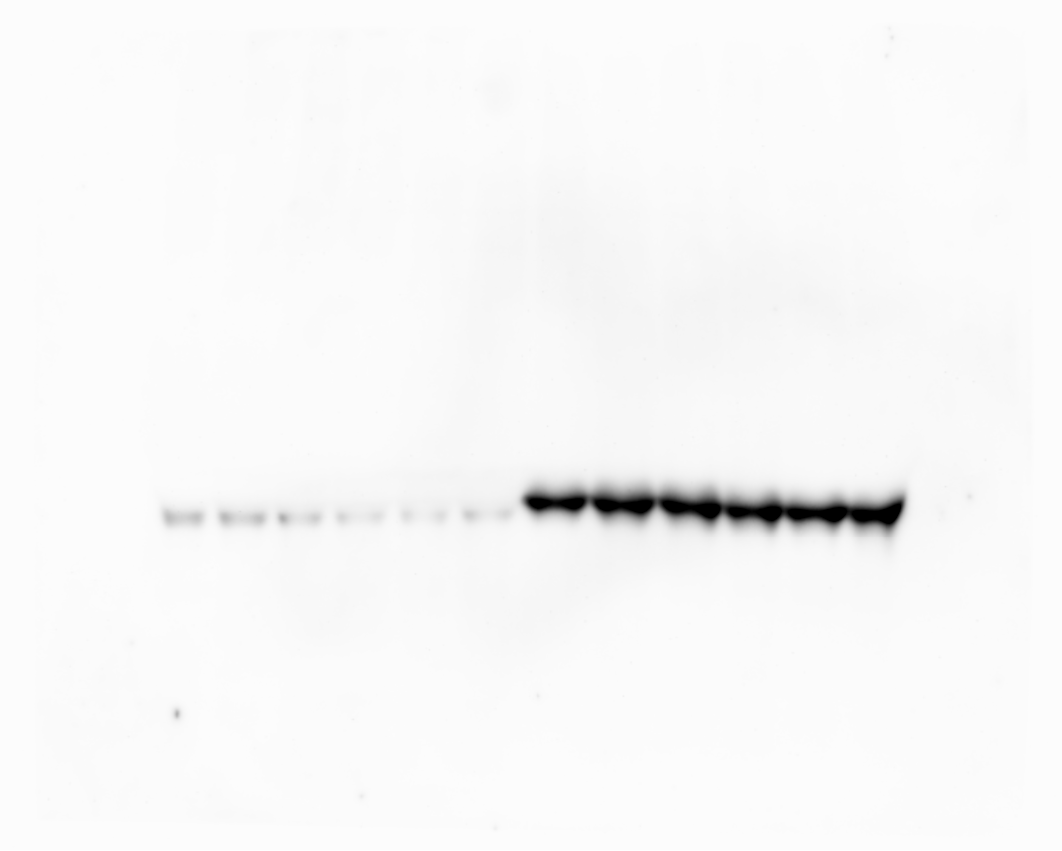

Supplement: Figure 4—source data 2. [file elife-81559-fig4-data2.zip › Figure 4-source data 2/B6 AAV Plasma a-LRG1.tif]

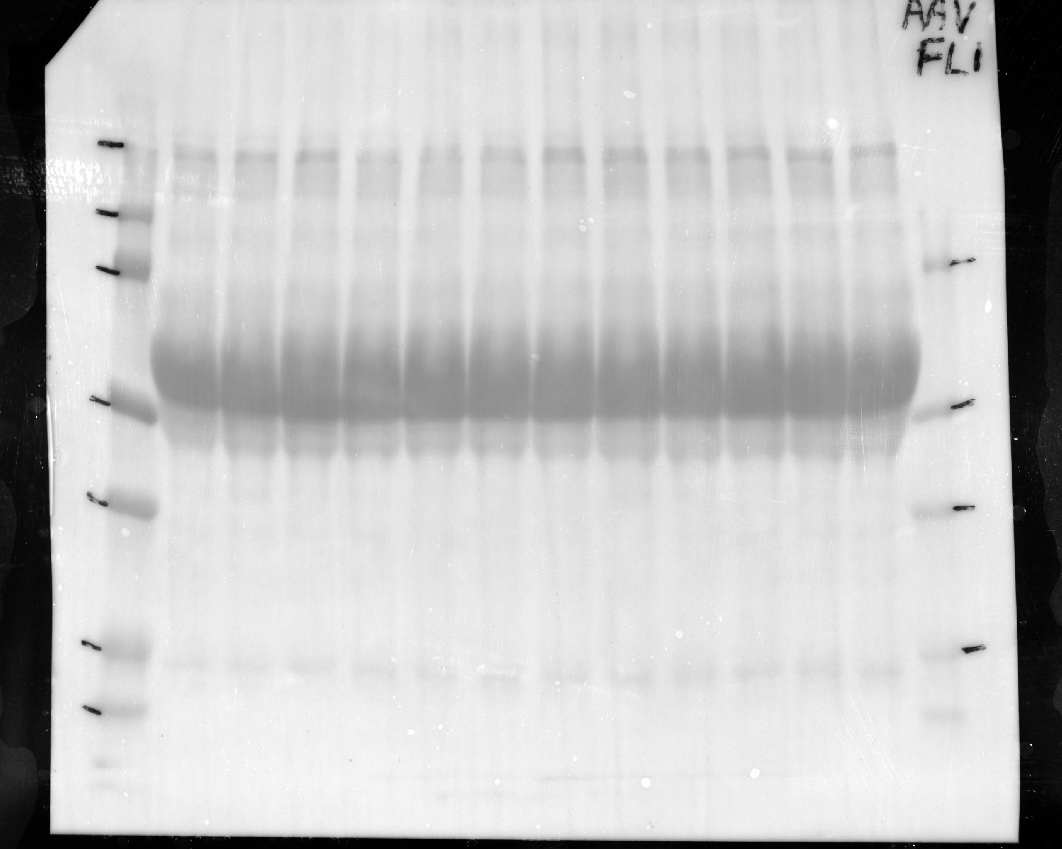

Supplement: Figure 4—source data 2. [file elife-81559-fig4-data2.zip › Figure 4-source data 2/B6 AAV Plasma Ponceau S.tif]

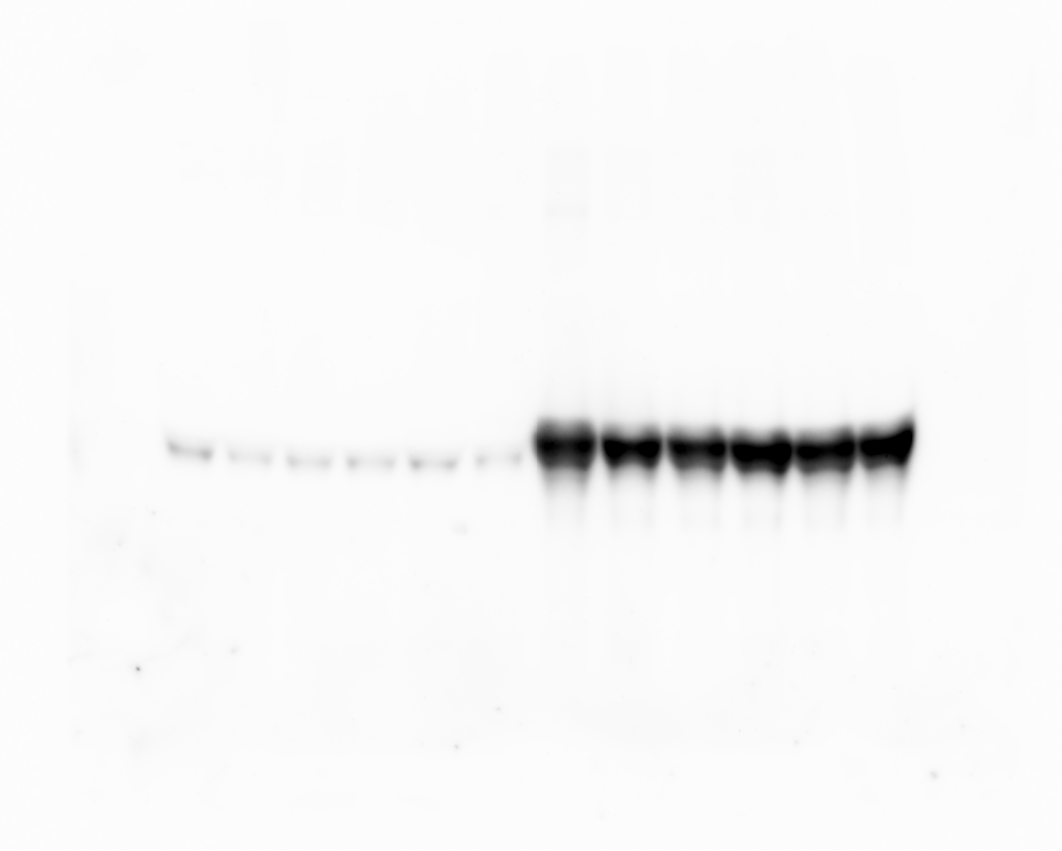

Supplement: Figure 4—source data 2. [file elife-81559-fig4-data2.zip › Figure 4-source data 2/B6 AdV Plasma a-LRG1.tif]

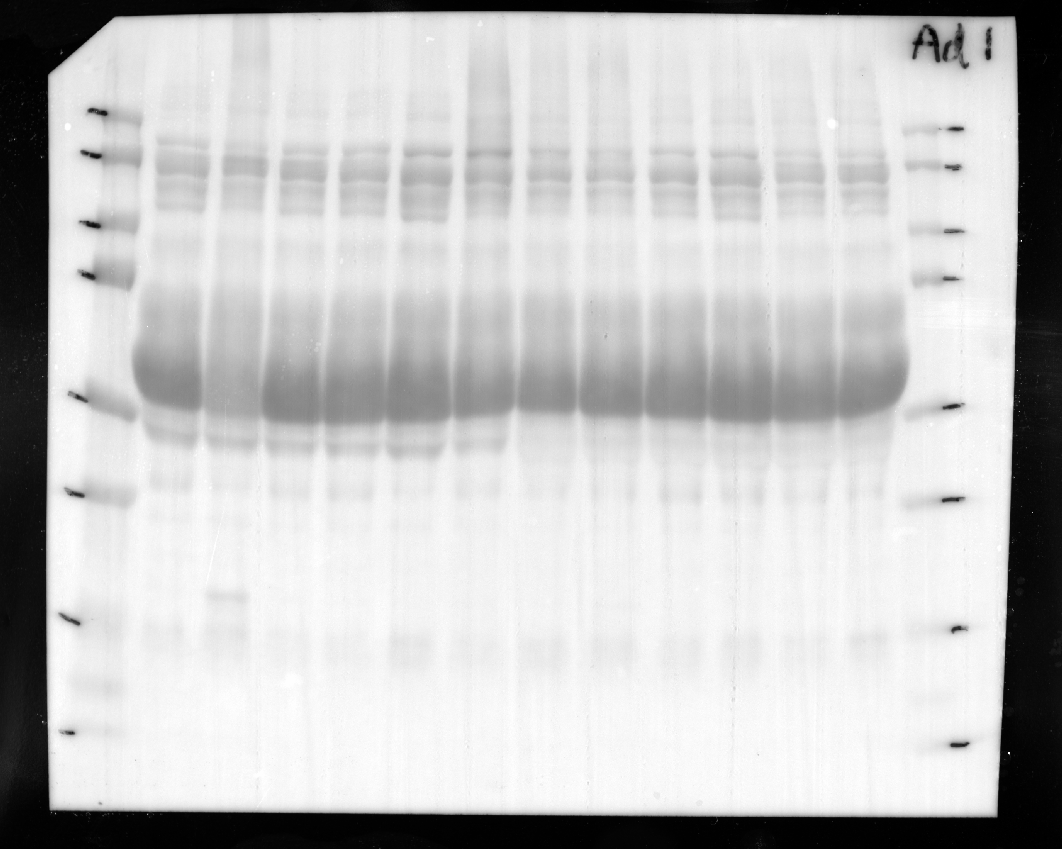

Supplement: Figure 4—source data 2. [file elife-81559-fig4-data2.zip › Figure 4-source data 2/B6 AdV Plasma Ponceau S.tif]

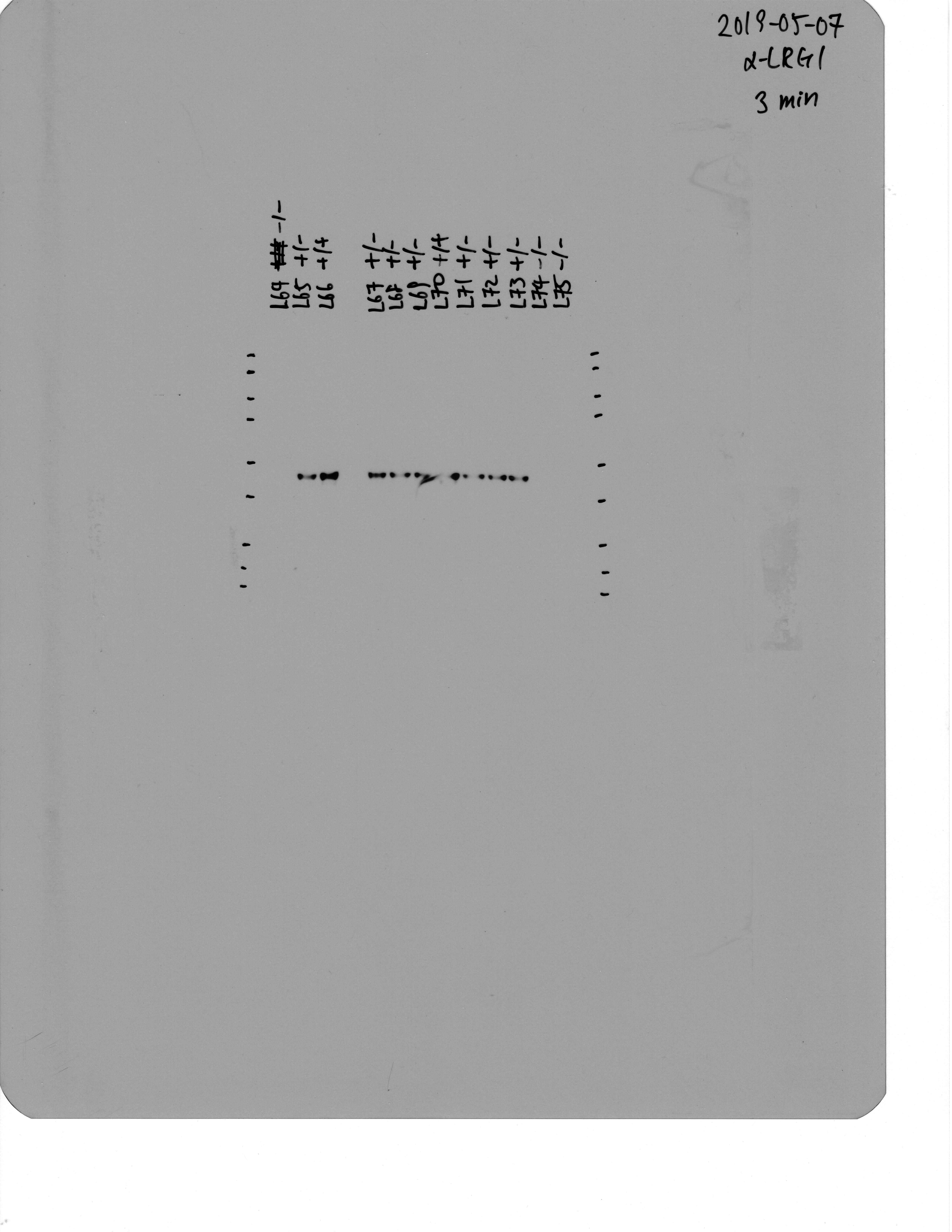

Supplement: Figure 4—source data 2. [file elife-81559-fig4-data2.zip › Figure 4-source data 2/Figure 4-S2/WT Het KO Plasma a-LRG1 3min.jpg]

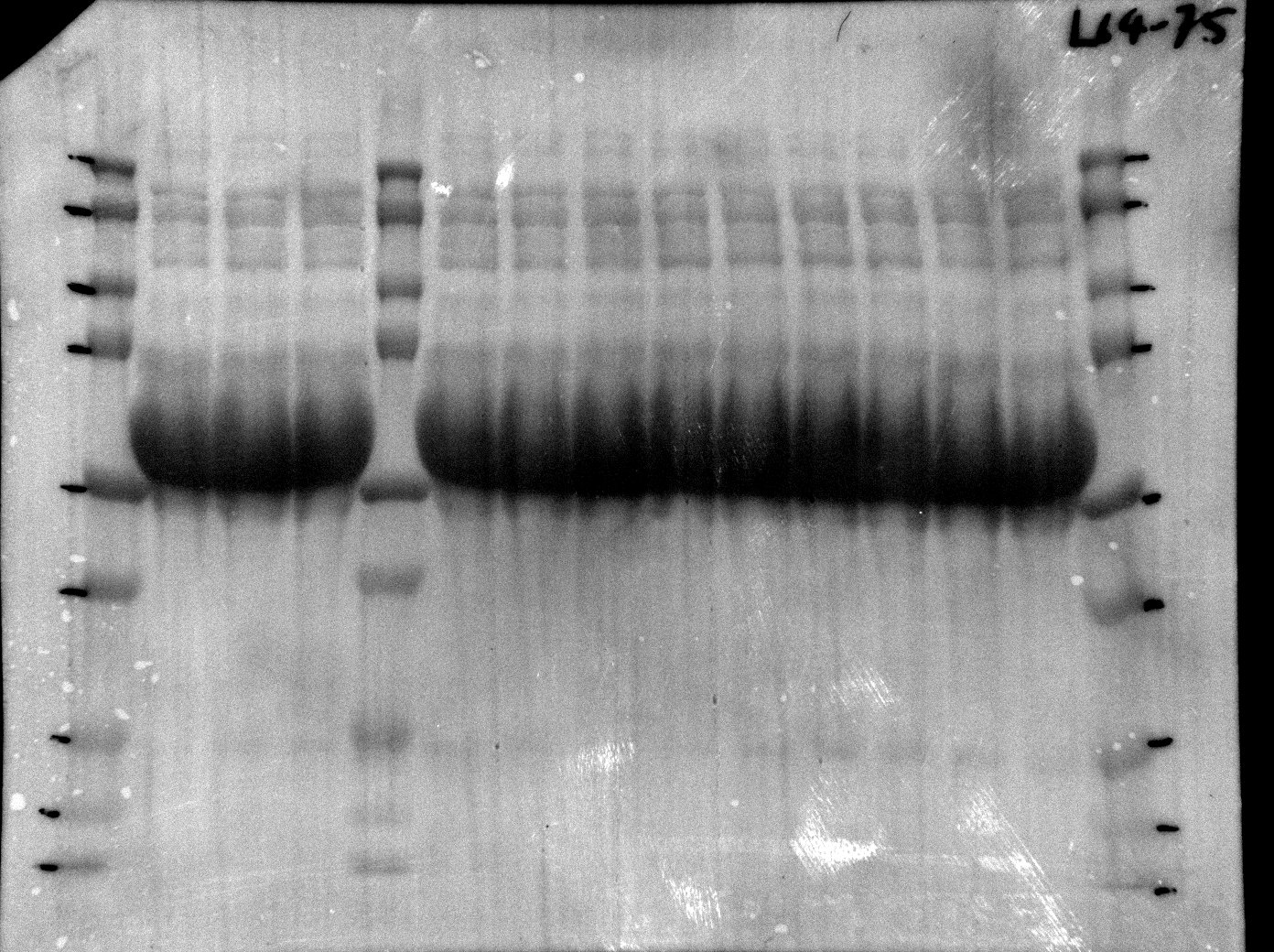

Supplement: Figure 4—source data 2. [file elife-81559-fig4-data2.zip › Figure 4-source data 2/Figure 4-S2/WT Het KO Plasma Ponceau S.jpg]

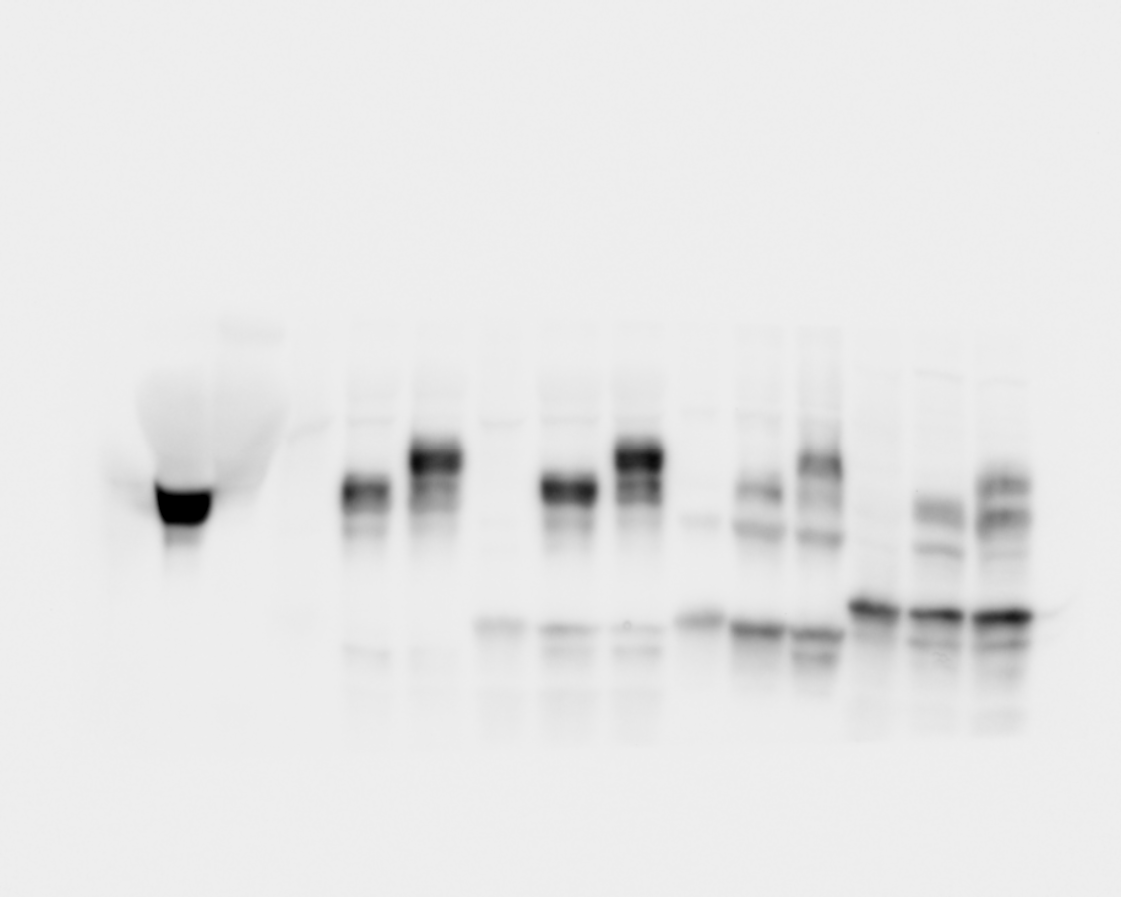

Supplement: Figure 4—source data 2. [file elife-81559-fig4-data2.zip › Figure 4-source data 2/WT LRG1-KO and AAV Tissues a-LRG1.tif]

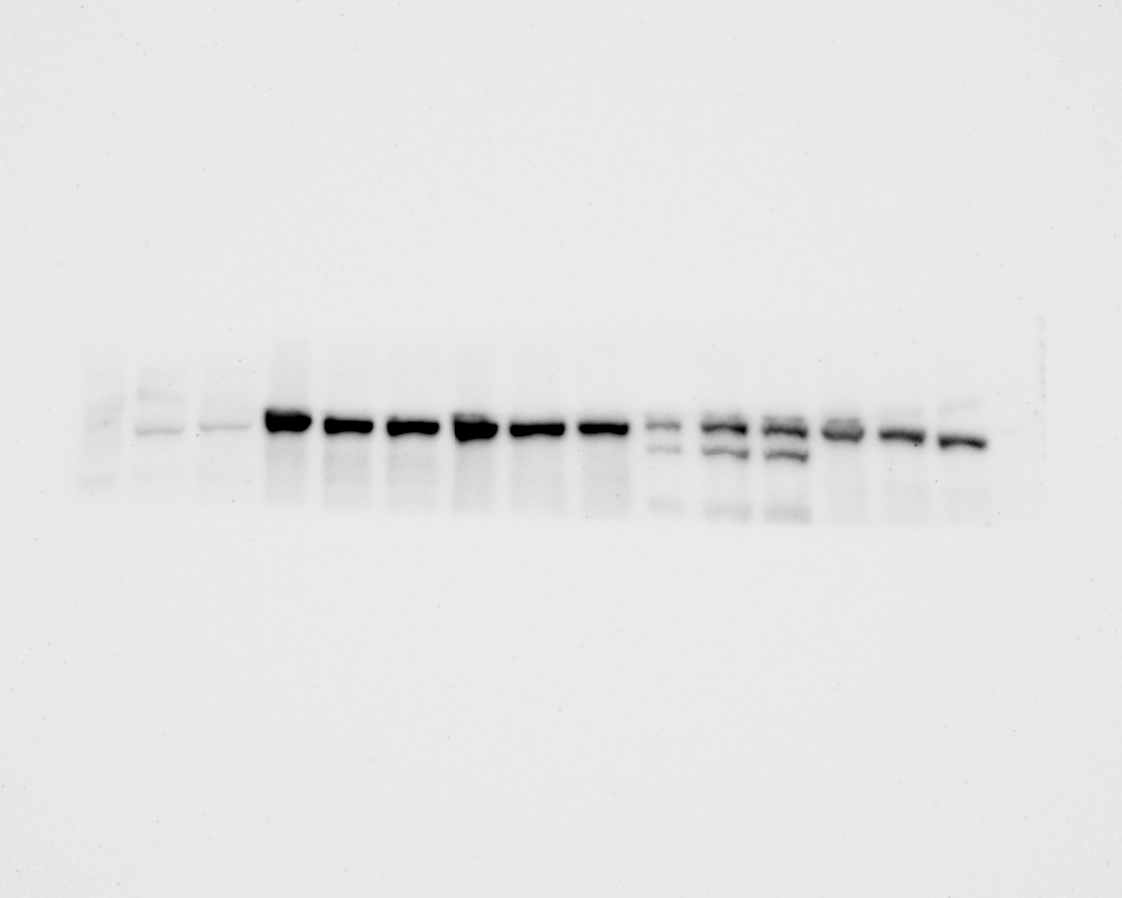

Supplement: Figure 4—source data 2. [file elife-81559-fig4-data2.zip › Figure 4-source data 2/WT LRG1-KO and AAV Tissues a-Vinculin.tif]

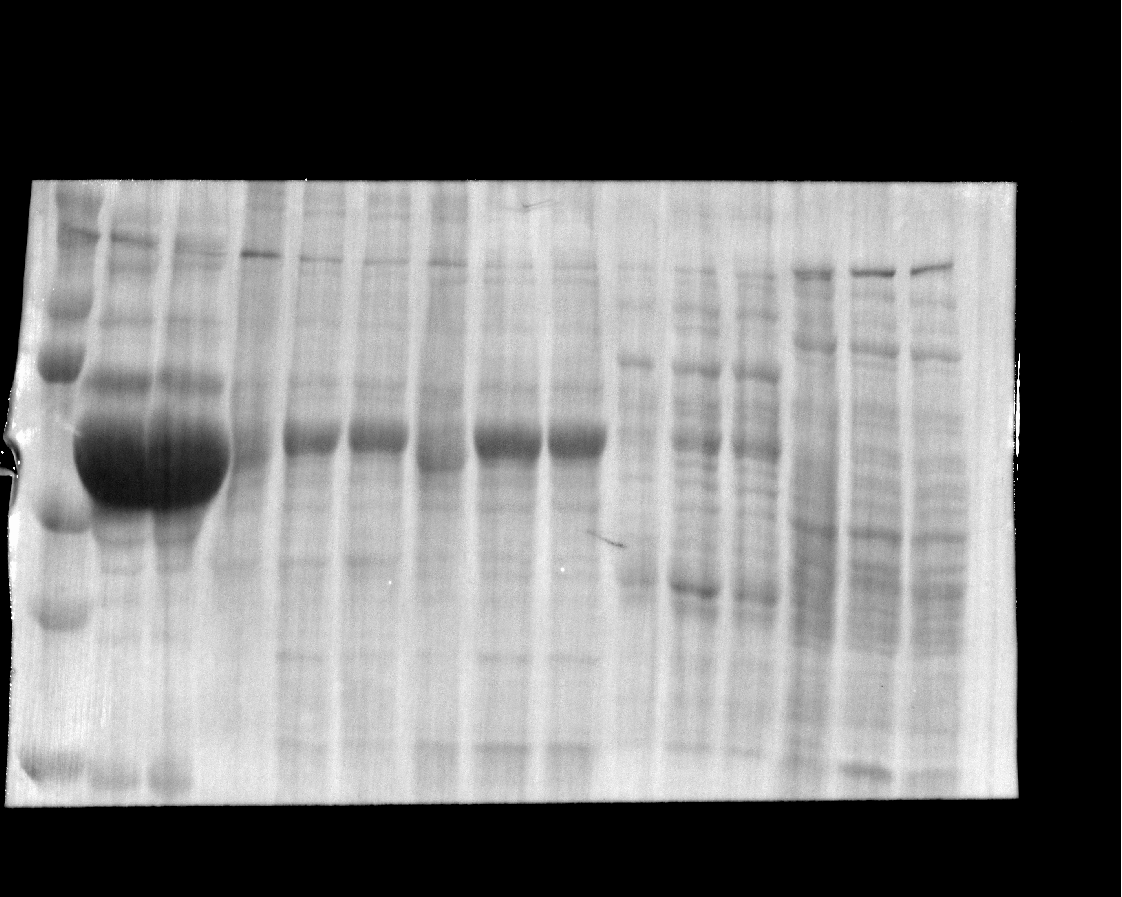

Supplement: Figure 4—source data 2. [file elife-81559-fig4-data2.zip › Figure 4-source data 2/WT LRG1-KO and AAV Tissues Ponceau S.tif]

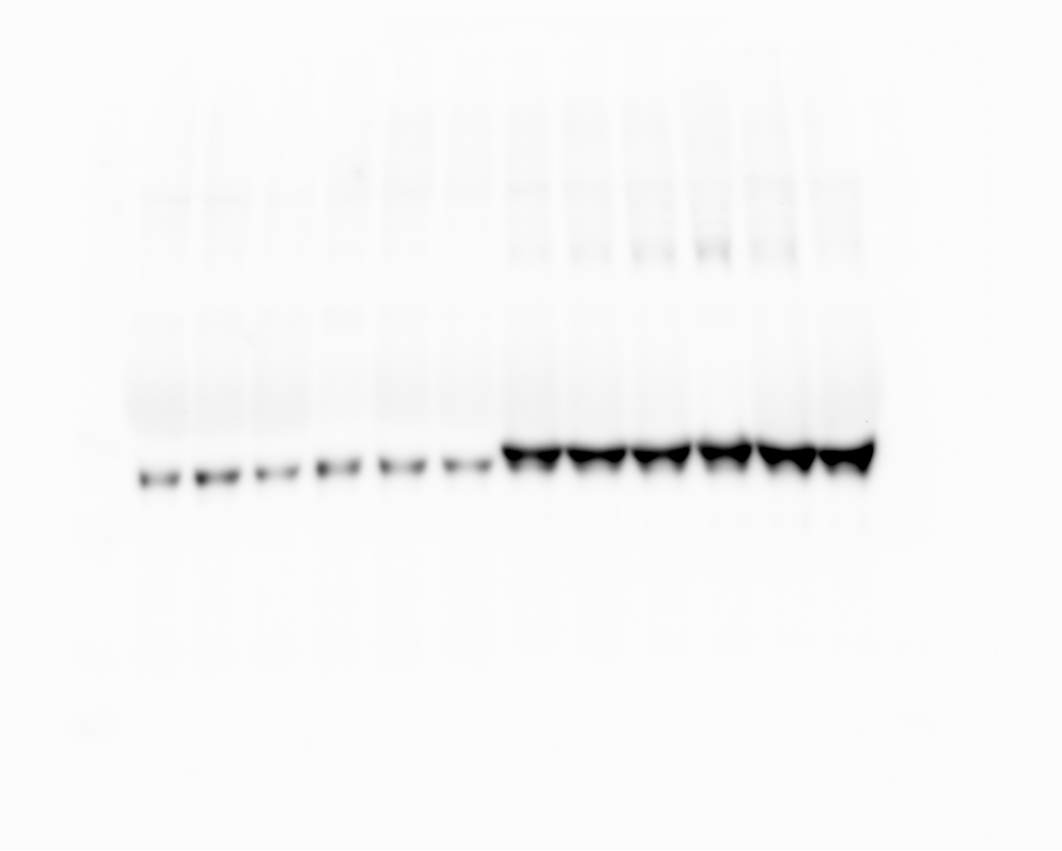

Supplement: Figure 5—source data 2. [file elife-81559-fig5-data2.zip › Figure 5-source data 2/db-db AAV Plasma a-LRG1.tif]

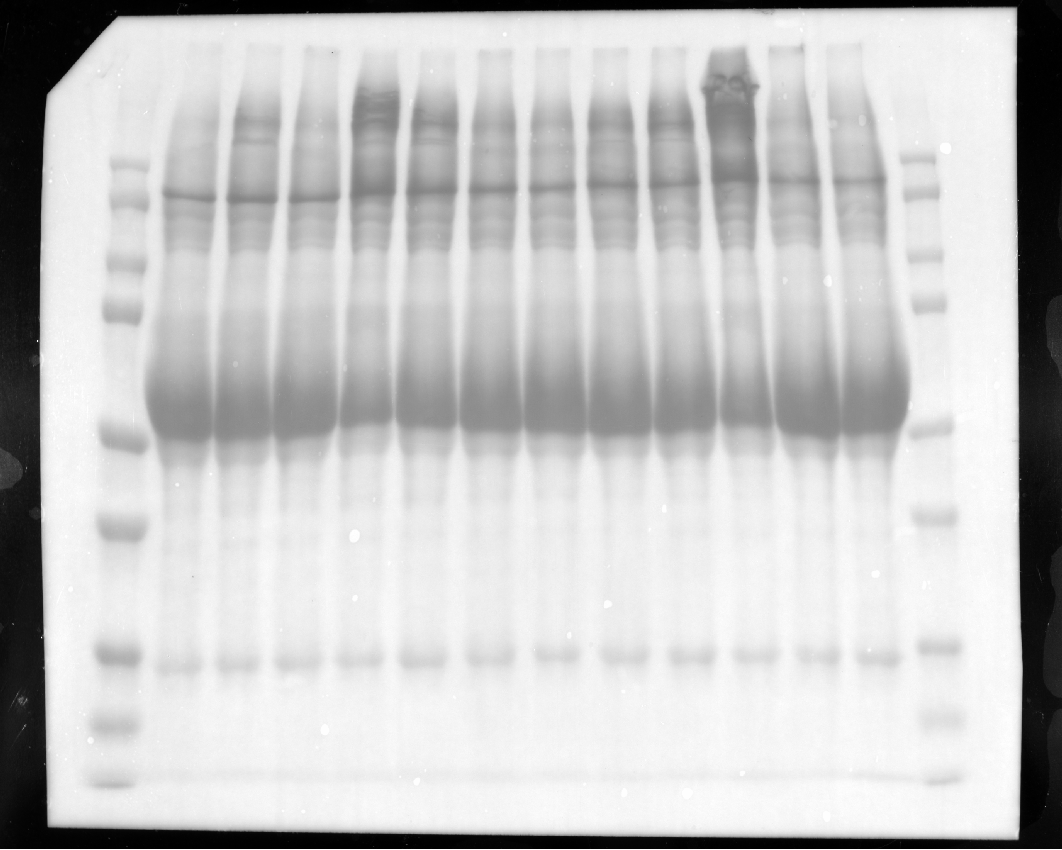

Supplement: Figure 5—source data 2. [file elife-81559-fig5-data2.zip › Figure 5-source data 2/db-db AAV Plasma Ponceau S.tif]

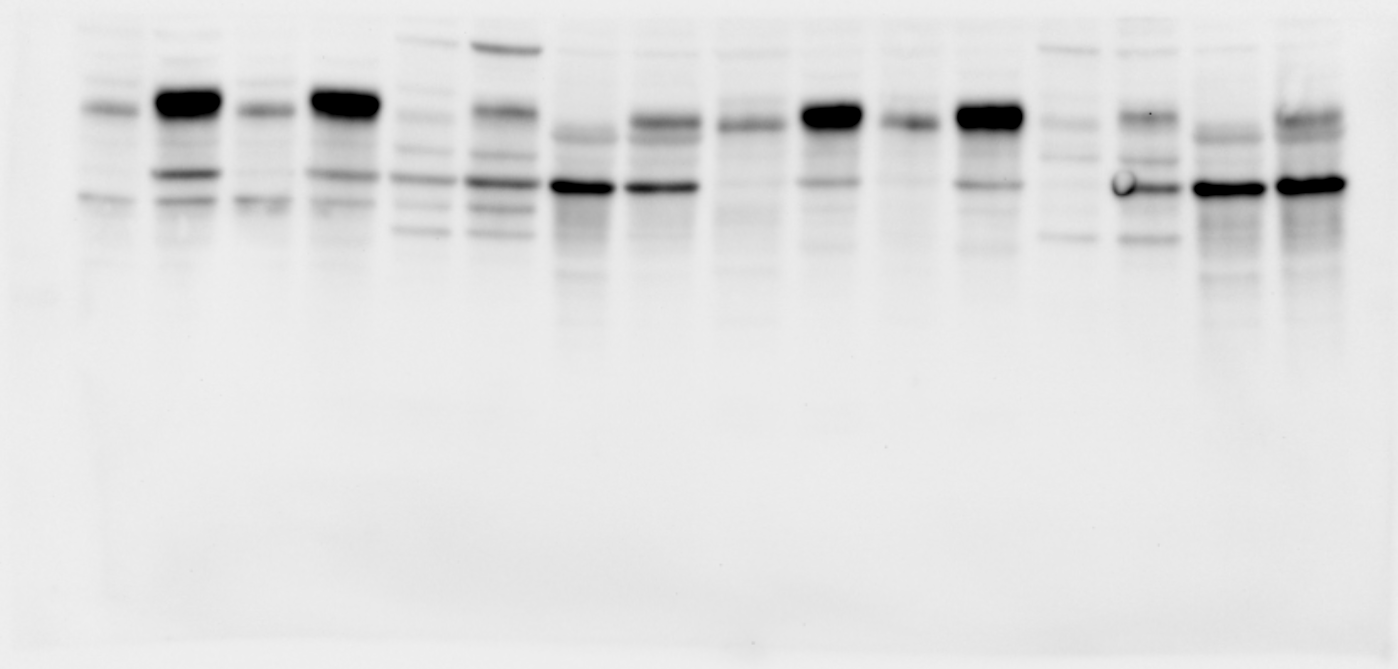

Supplement: Figure 5—source data 2. [file elife-81559-fig5-data2.zip › Figure 5-source data 2/Figure 5-S1/db-db AAV Tissues a-LRG1.tif]

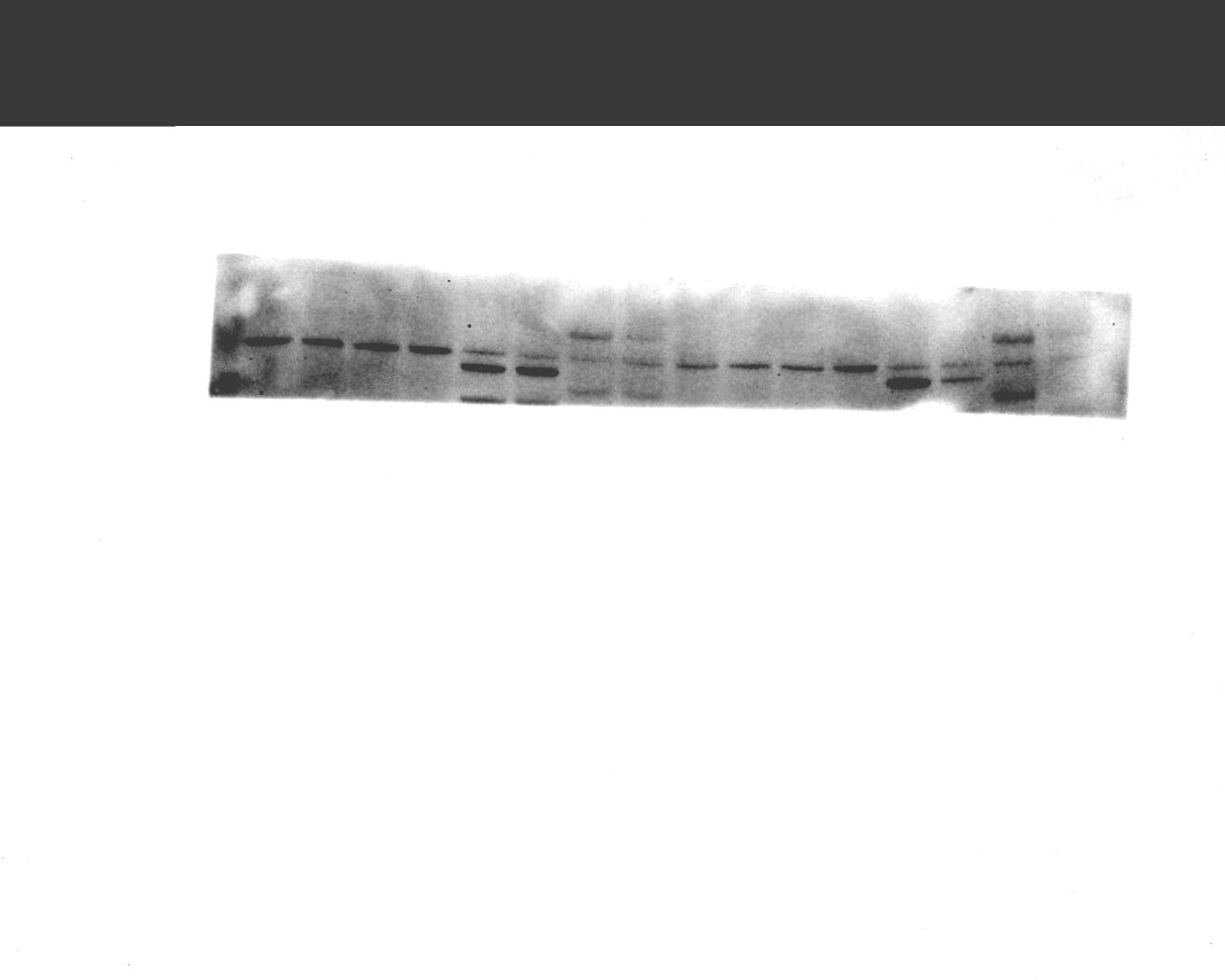

Supplement: Figure 5—source data 2. [file elife-81559-fig5-data2.zip › Figure 5-source data 2/Figure 5-S1/db-db AAV Tissues a-Vinculin.tif]

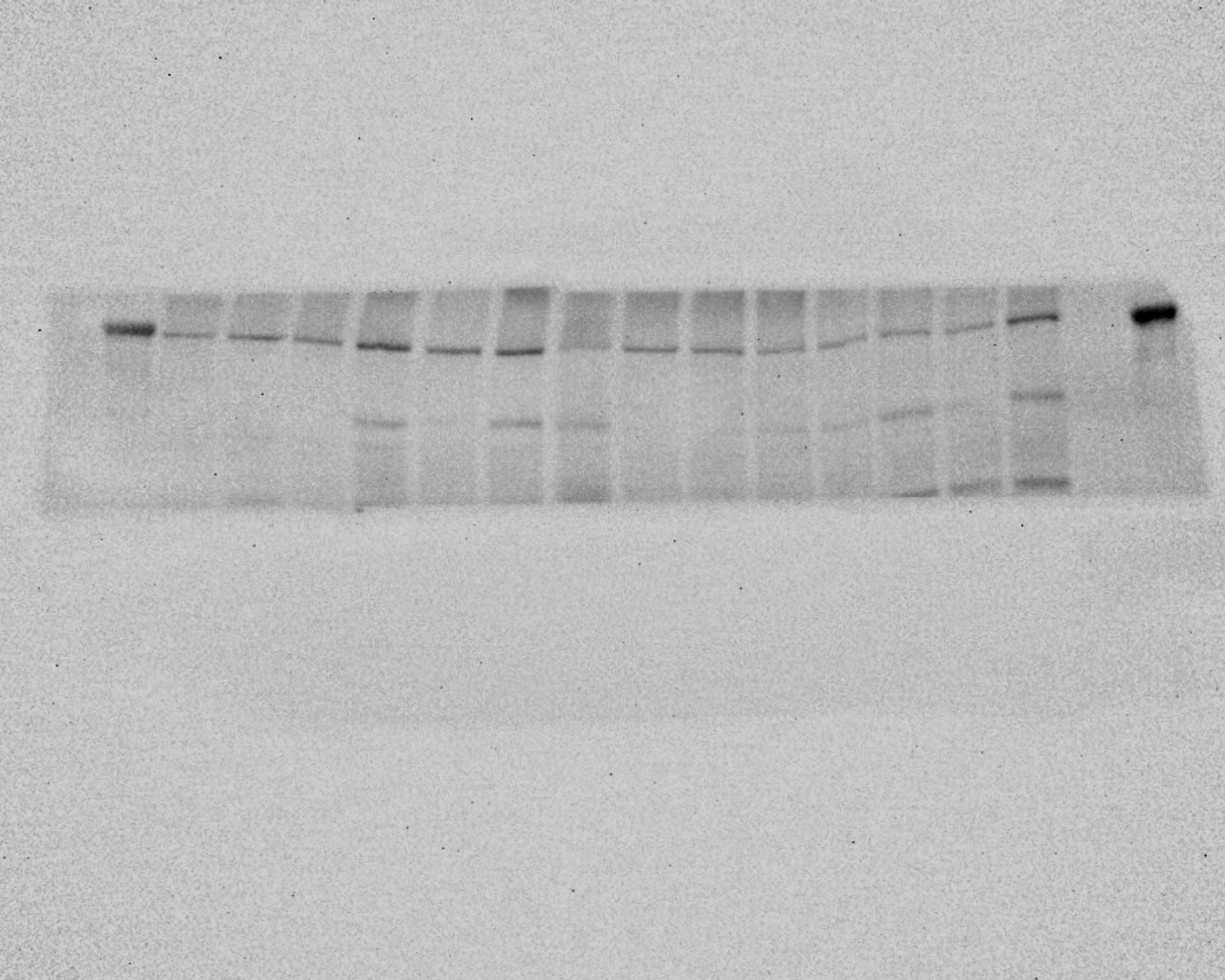

Supplement: Figure 5—source data 2. [file elife-81559-fig5-data2.zip › Figure 5-source data 2/Figure 5-S1/db-db eWAT IRS-1.tif]

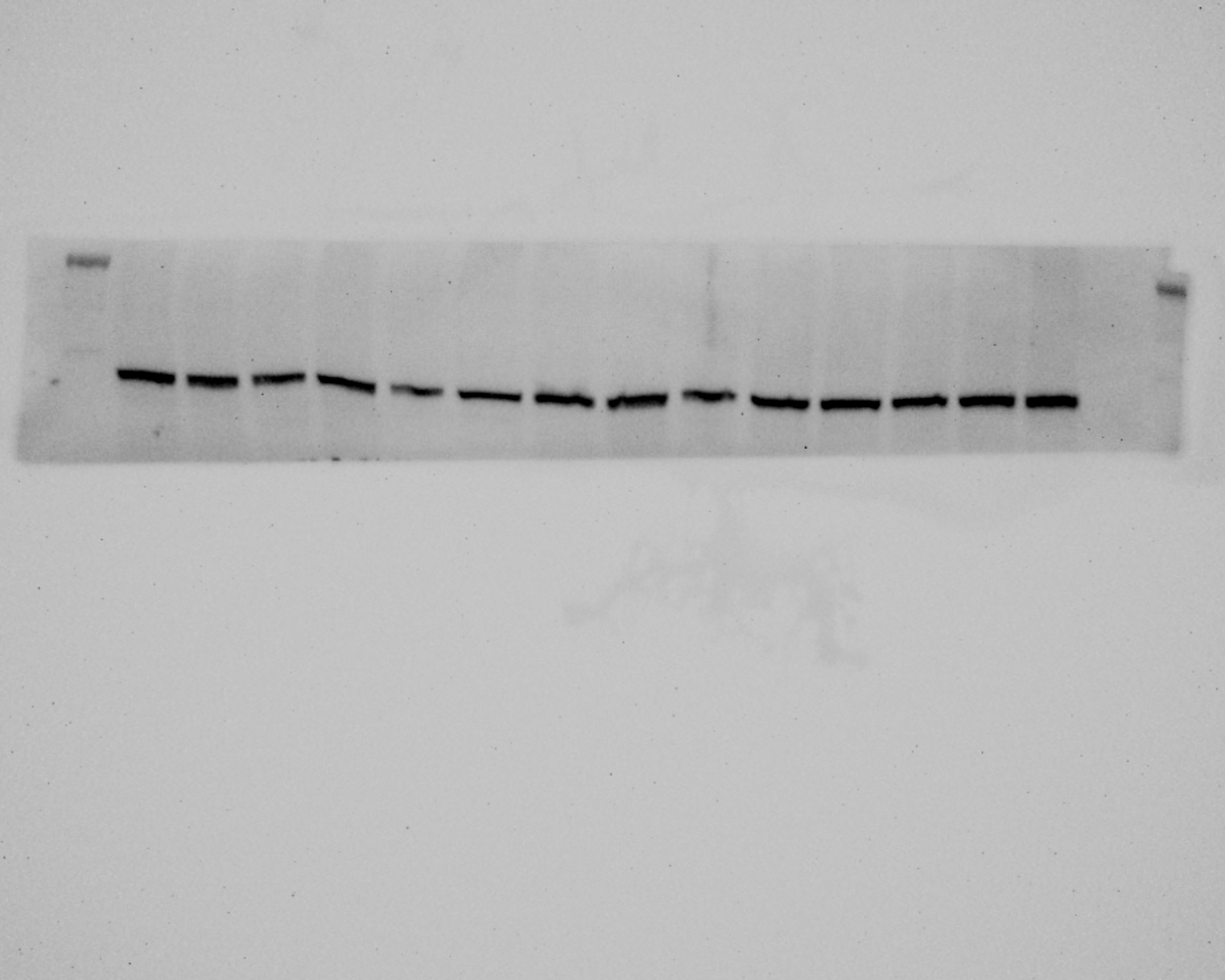

Supplement: Figure 5—source data 2. [file elife-81559-fig5-data2.zip › Figure 5-source data 2/Figure 5-S1/db-db eWAT Vinculin.tif]

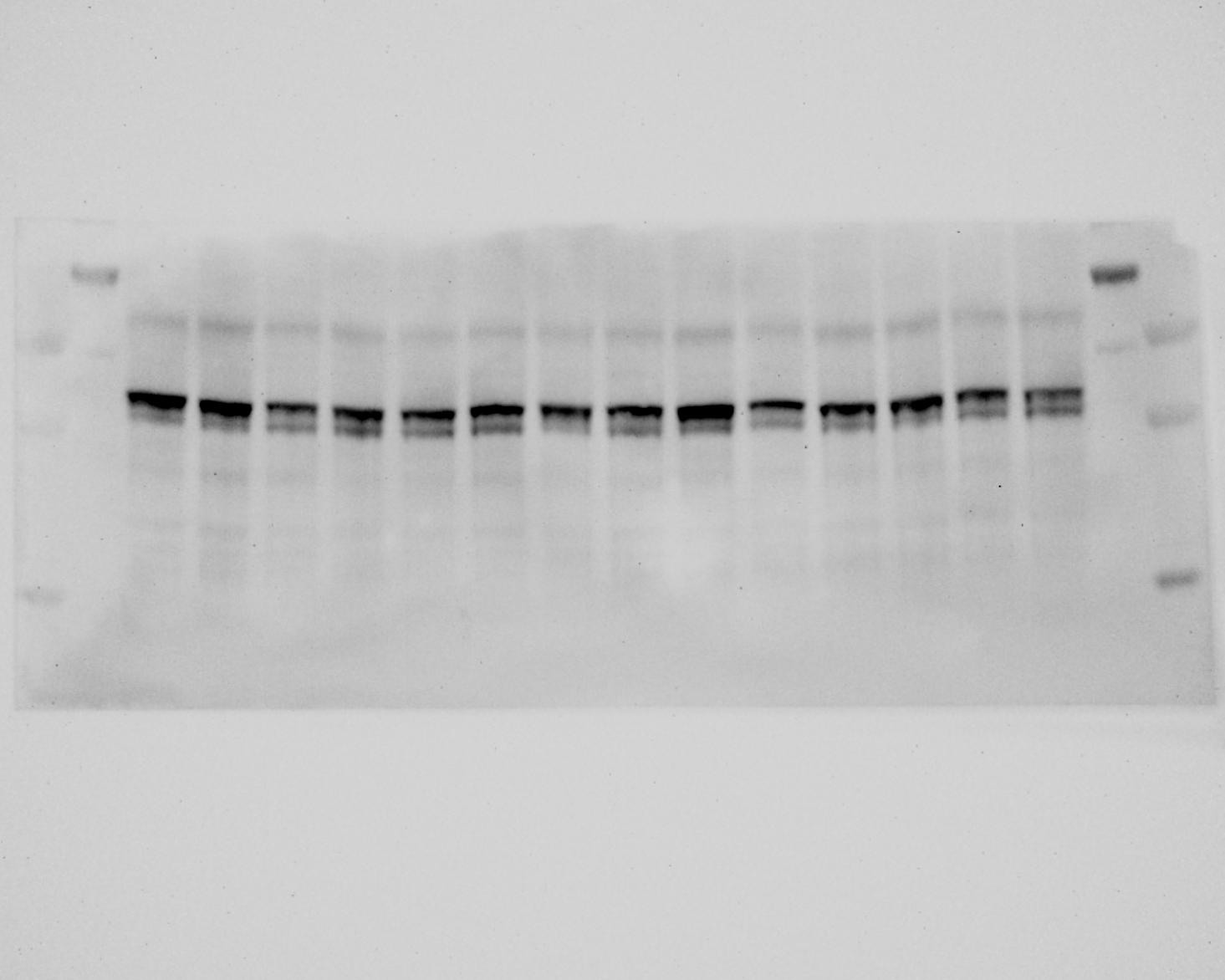

Supplement: Figure 5—source data 2. [file elife-81559-fig5-data2.zip › Figure 5-source data 2/Figure 5-S1/db-db Liver b-actin.tif]

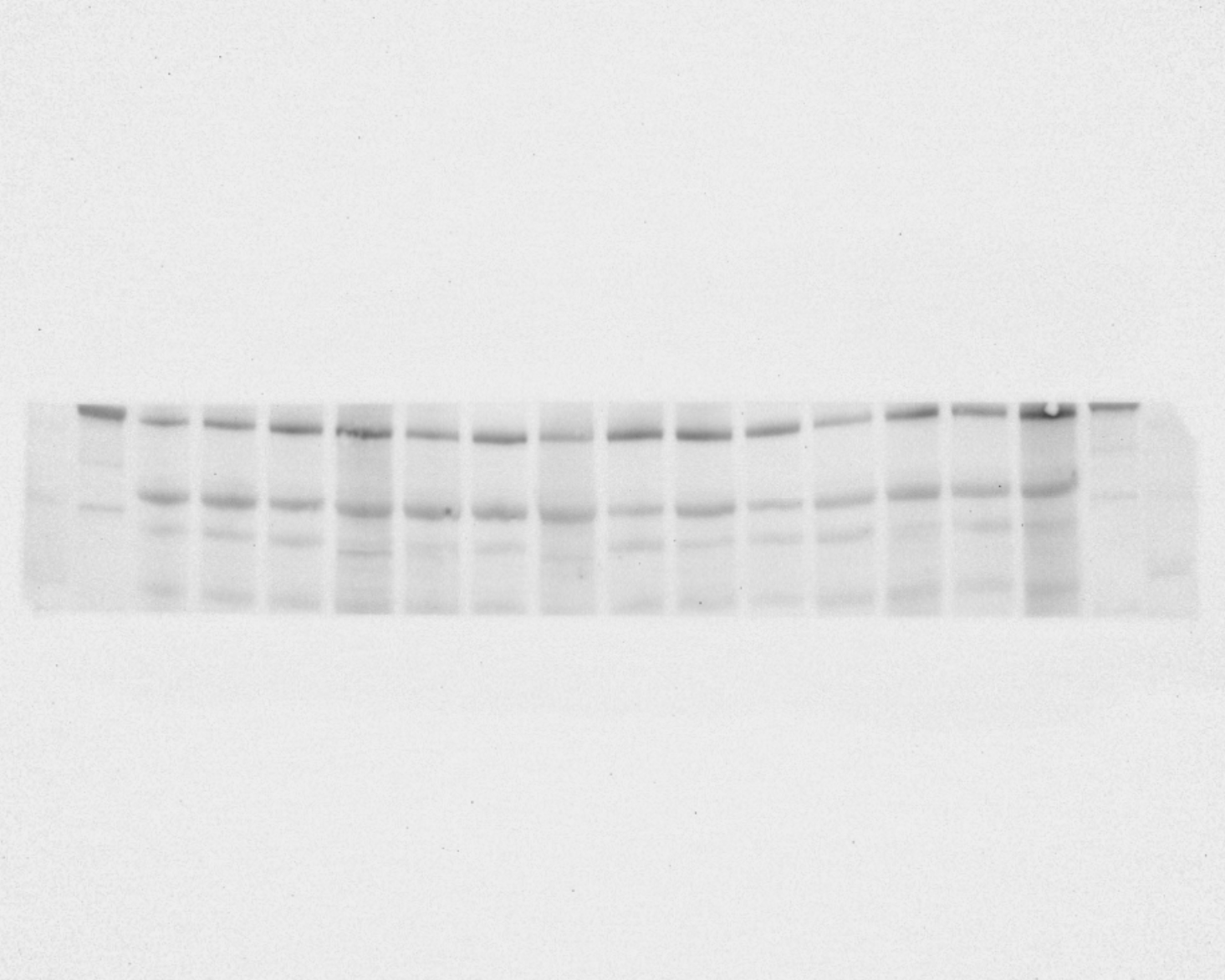

Supplement: Figure 5—source data 2. [file elife-81559-fig5-data2.zip › Figure 5-source data 2/Figure 5-S1/db-db Liver IRS-1.tif]

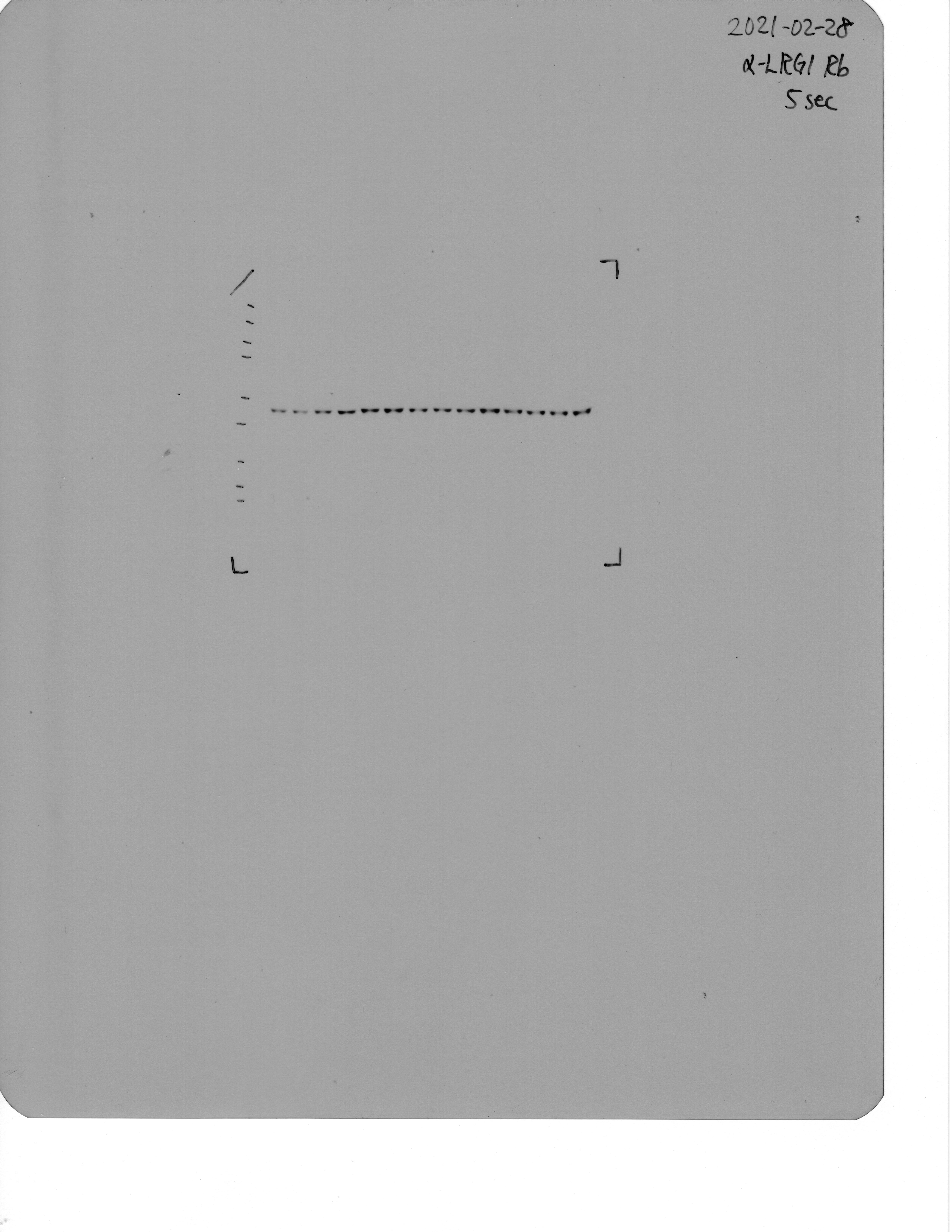

Supplement: Figure 5—source data 2. [file elife-81559-fig5-data2.zip › Figure 5-source data 2/Figure 5-S1/m-m vs db-db Serum a-LRG1 5s.jpg]

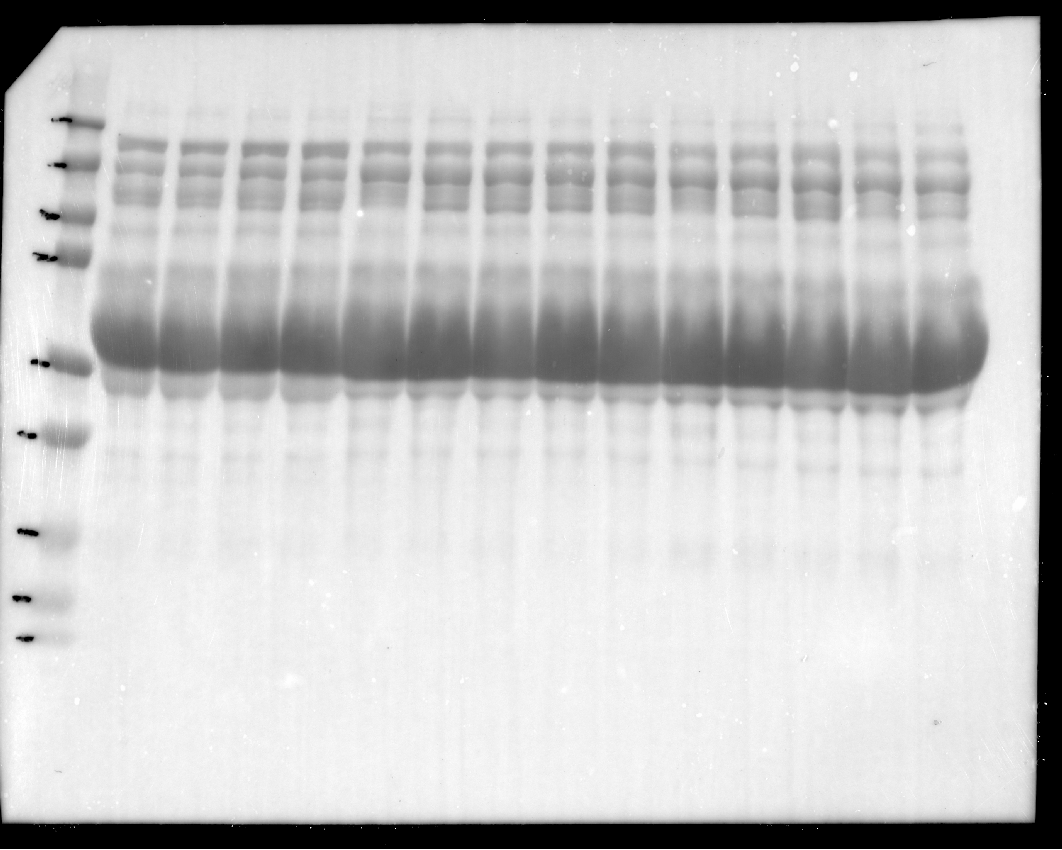

Supplement: Figure 5—source data 2. [file elife-81559-fig5-data2.zip › Figure 5-source data 2/Figure 5-S1/m-m vs db-db Serum Ponceau S.tif]

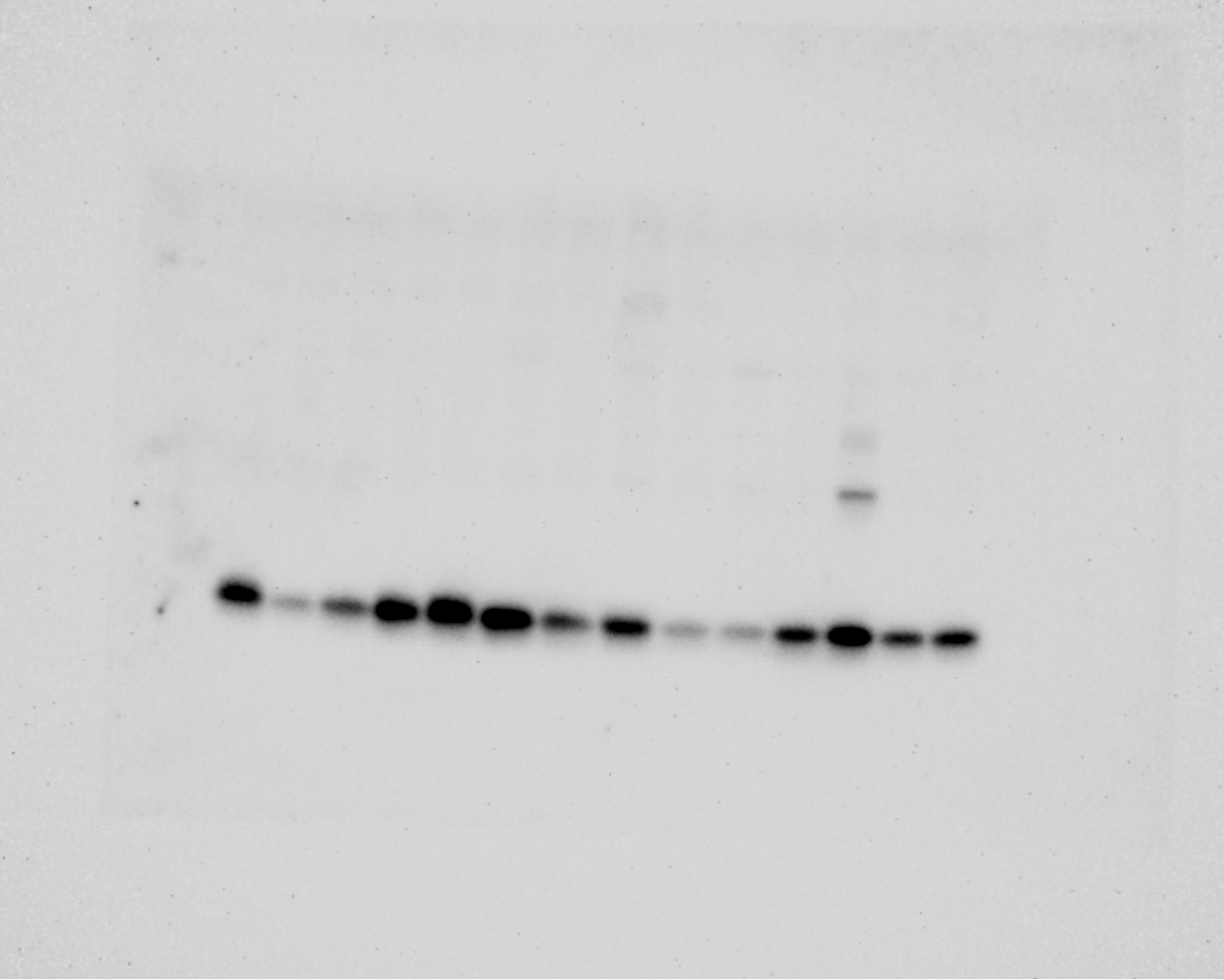

Supplement: Figure 7—source data 2. [file elife-81559-fig7-data2.zip › Figure 7-source data 2/B6 eWAT ISF a-Cyt c.tif]

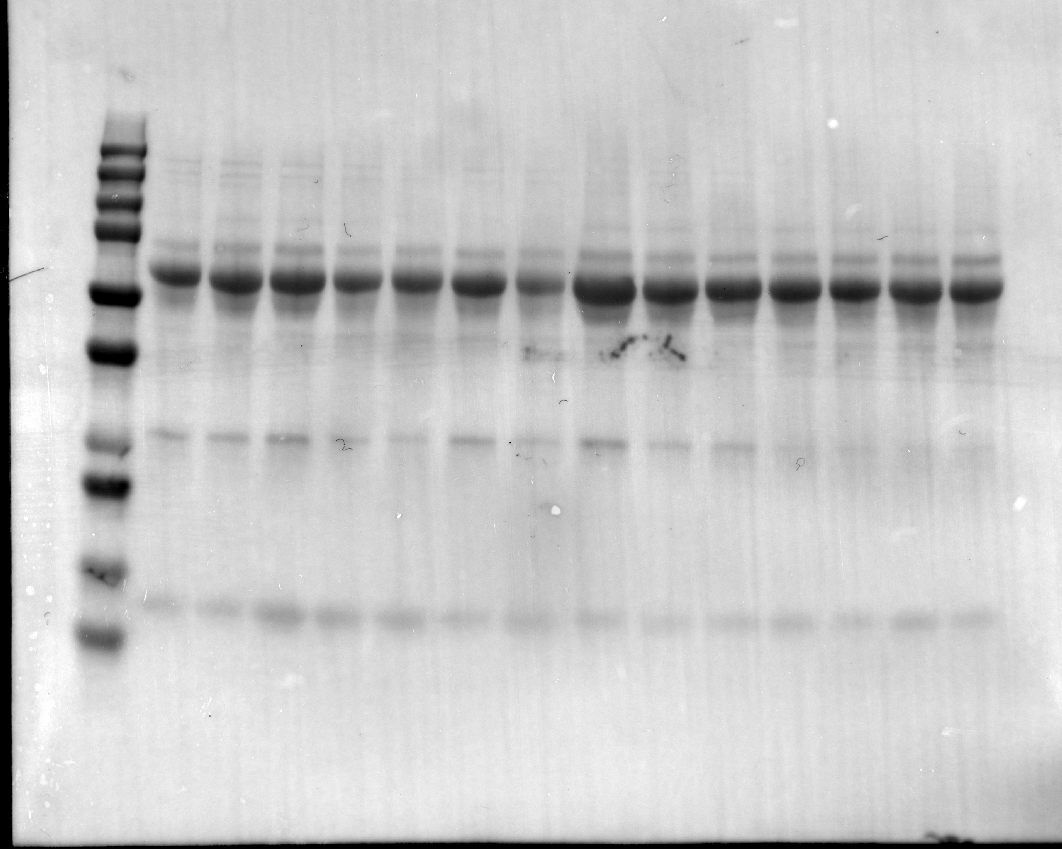

Supplement: Figure 7—source data 2. [file elife-81559-fig7-data2.zip › Figure 7-source data 2/B6 eWAT ISF Ponceau S.tif]

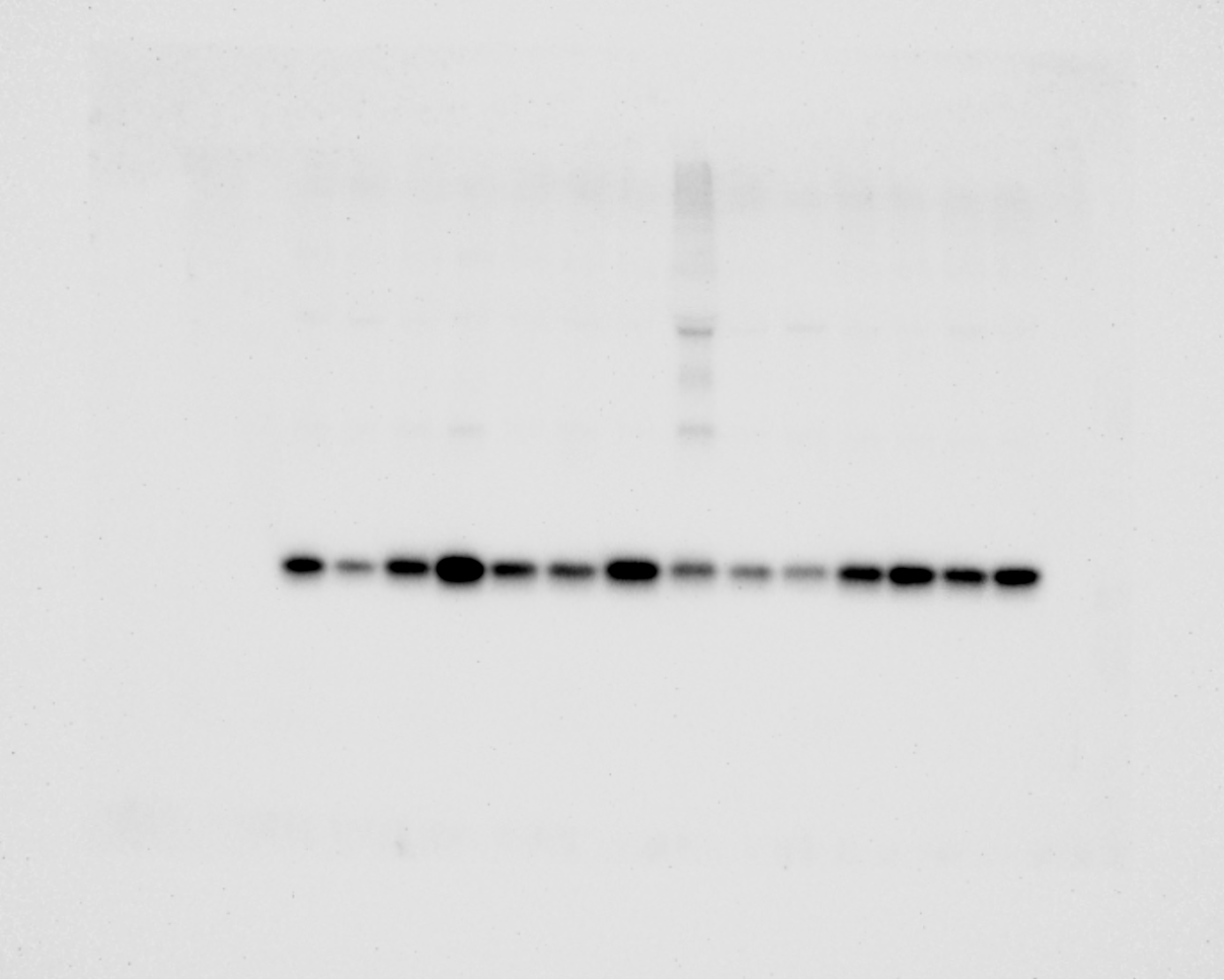

Supplement: Figure 7—source data 2. [file elife-81559-fig7-data2.zip › Figure 7-source data 2/B6 iWAT ISF a-Cyt c.tif]

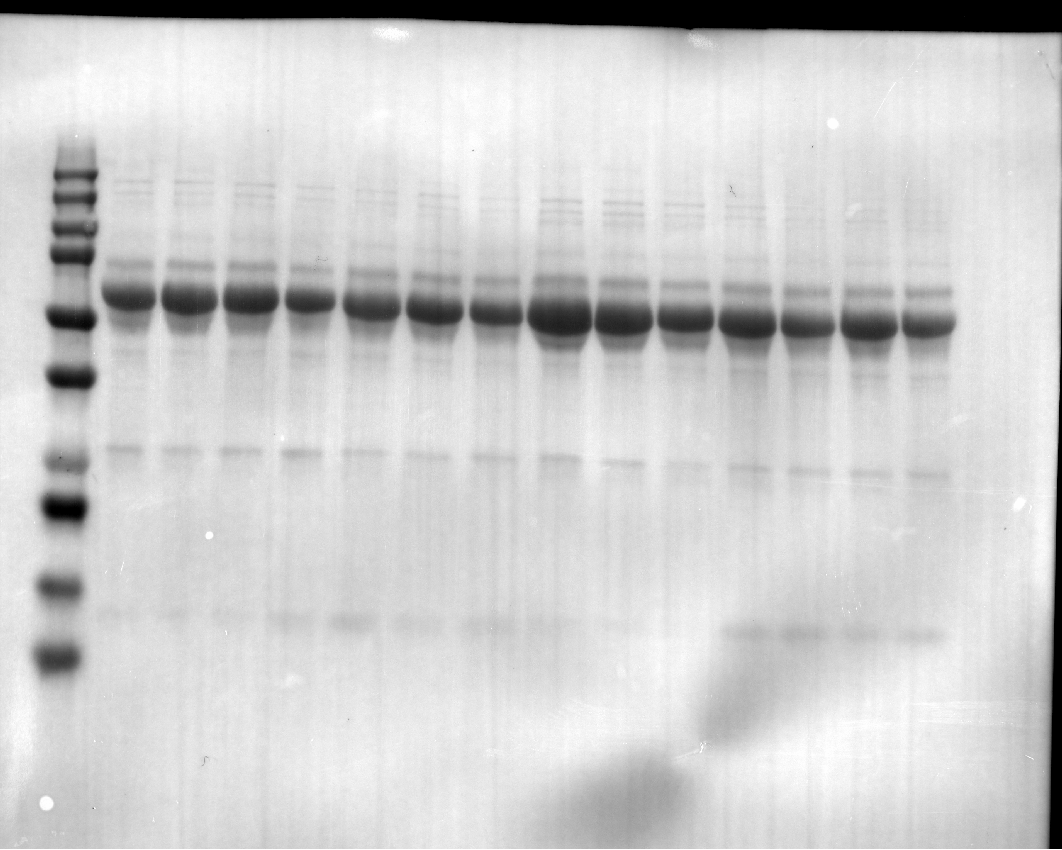

Supplement: Figure 7—source data 2. [file elife-81559-fig7-data2.zip › Figure 7-source data 2/B6 iWAT ISF Ponceau S.tif]

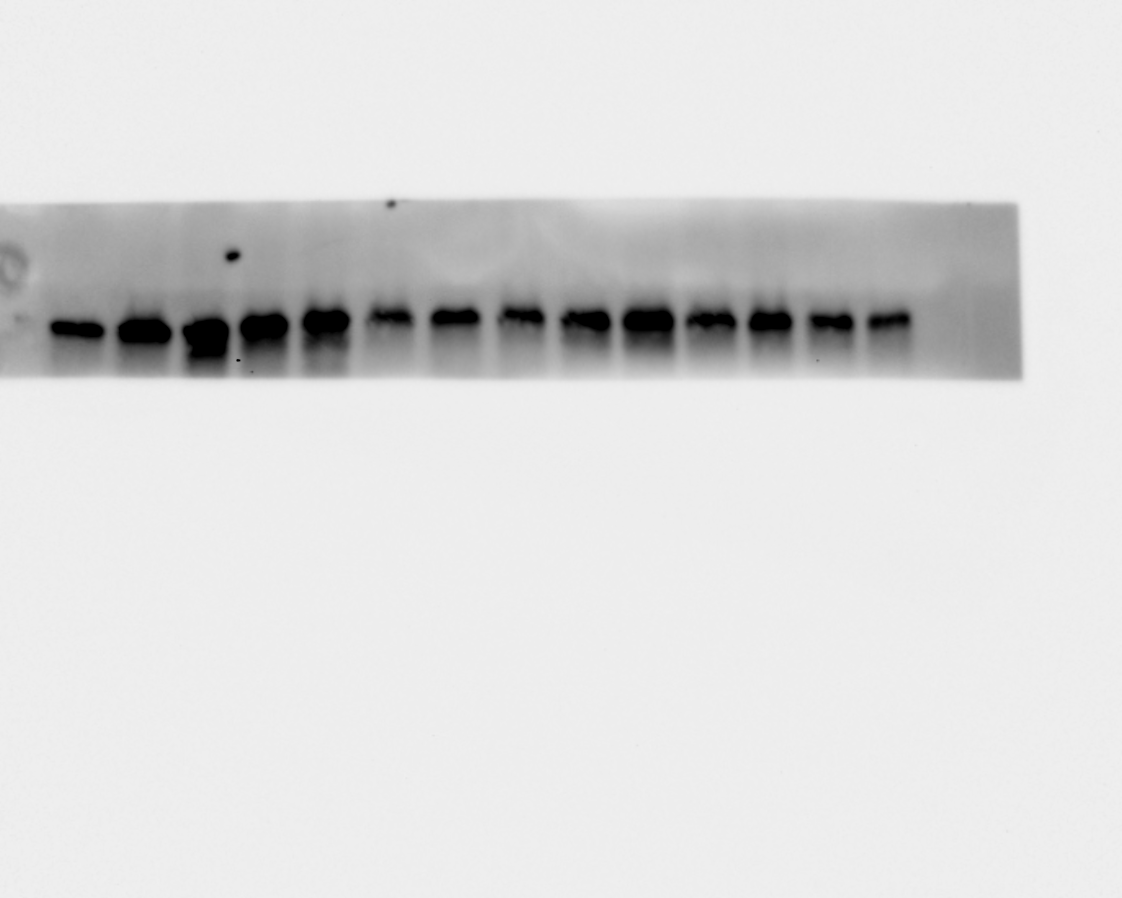

Supplement: Figure 7—source data 2. [file elife-81559-fig7-data2.zip › Figure 7-source data 2/db-db eWAT Caspase3.tif]

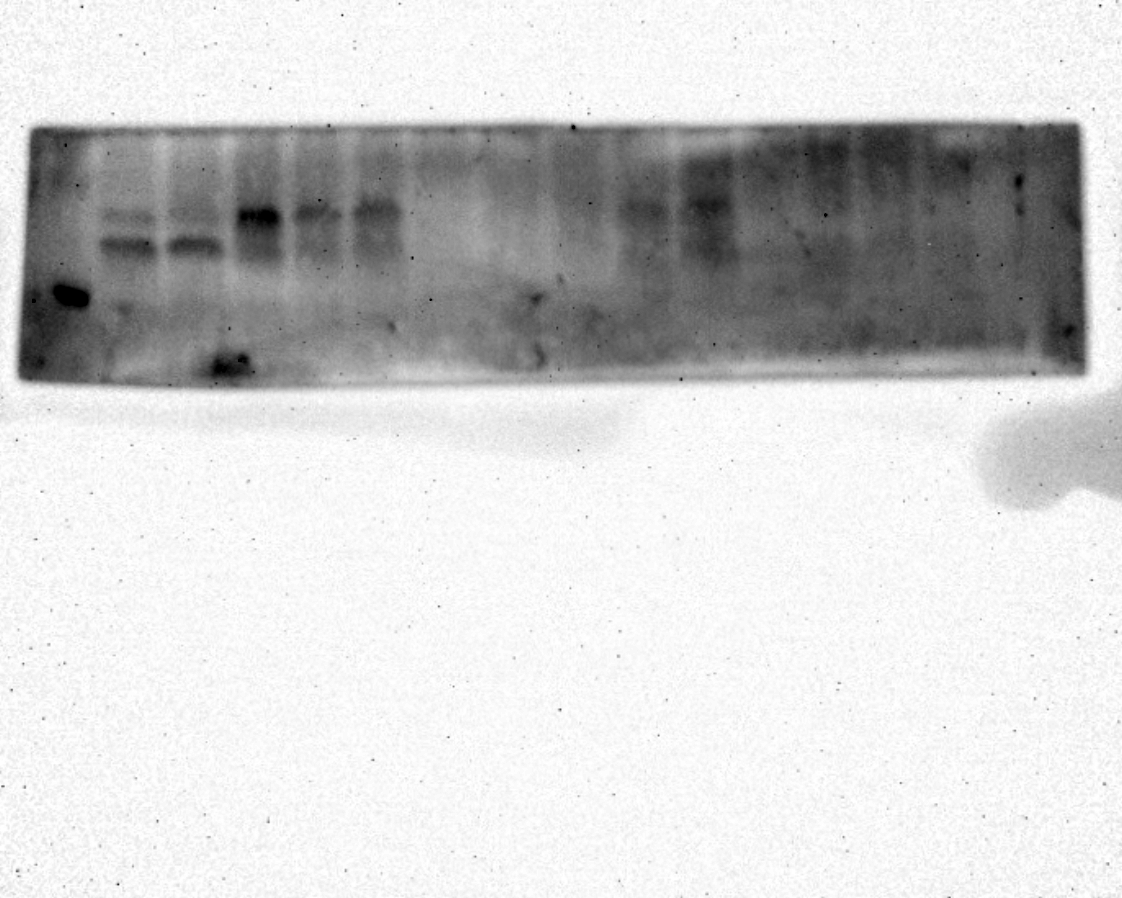

Supplement: Figure 7—source data 2. [file elife-81559-fig7-data2.zip › Figure 7-source data 2/db-db eWAT c-Caspase3.tif]

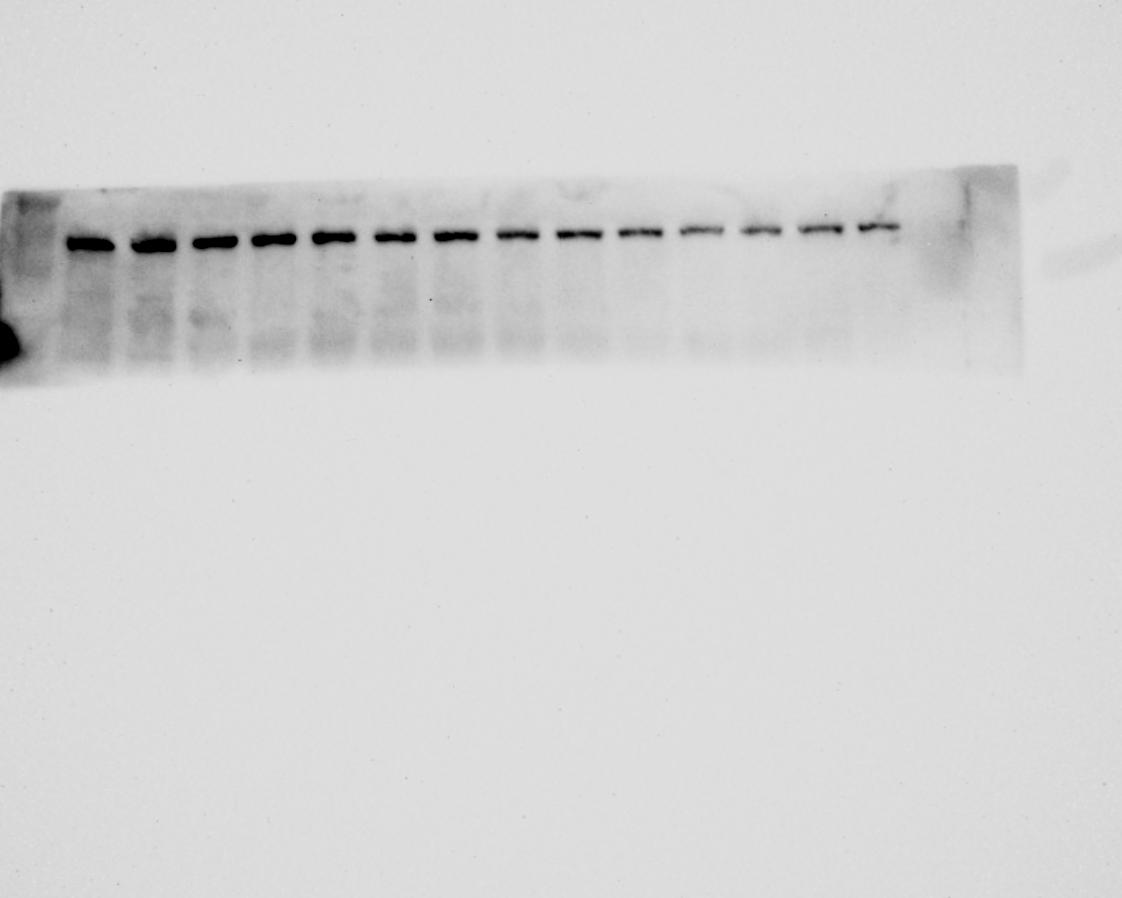

Supplement: Figure 7—source data 2. [file elife-81559-fig7-data2.zip › Figure 7-source data 2/db-db eWAT Vinculin.tif]

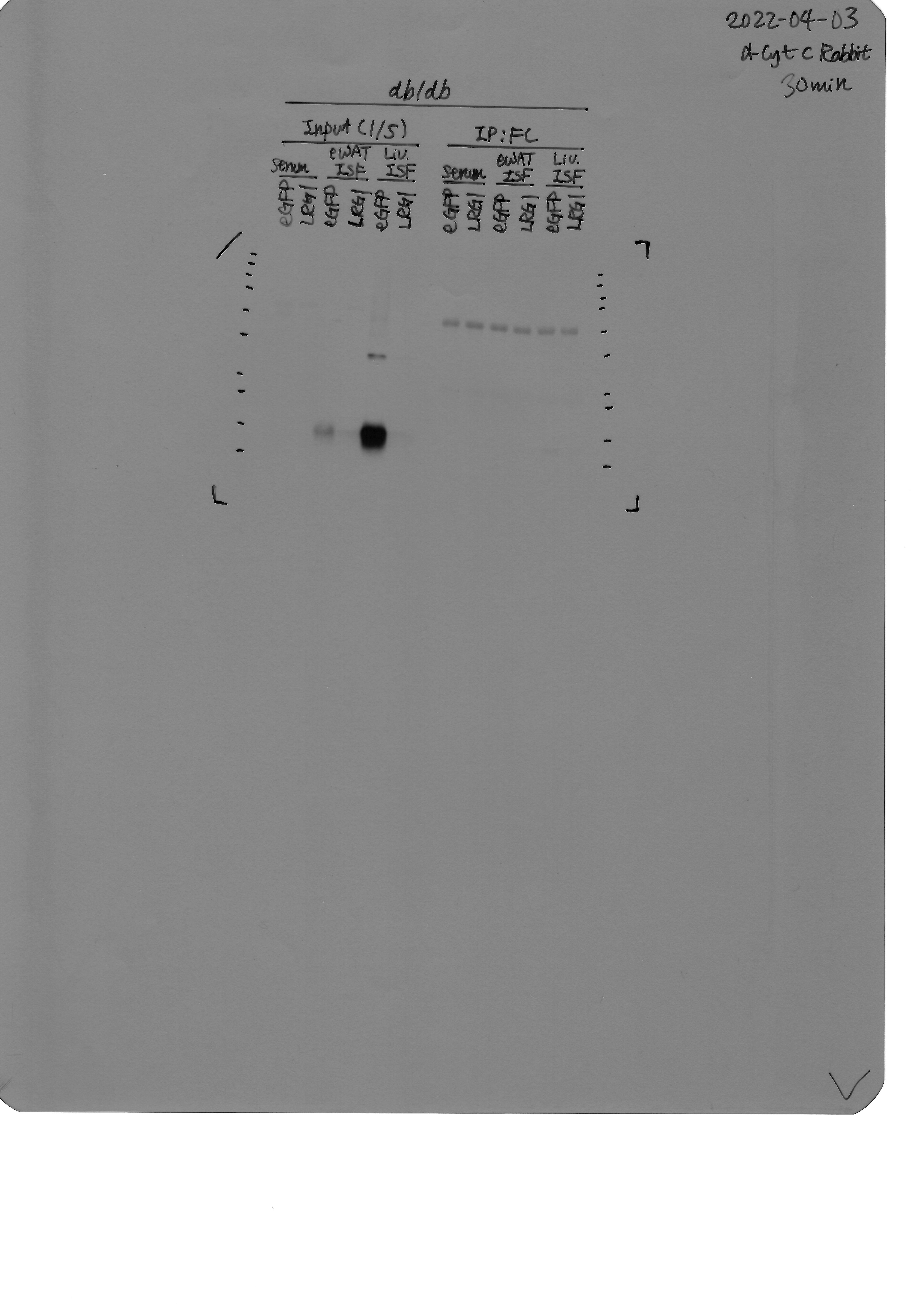

Supplement: Figure 7—source data 2. [file elife-81559-fig7-data2.zip › Figure 7-source data 2/db-db ISF a-Cyt c.tif]

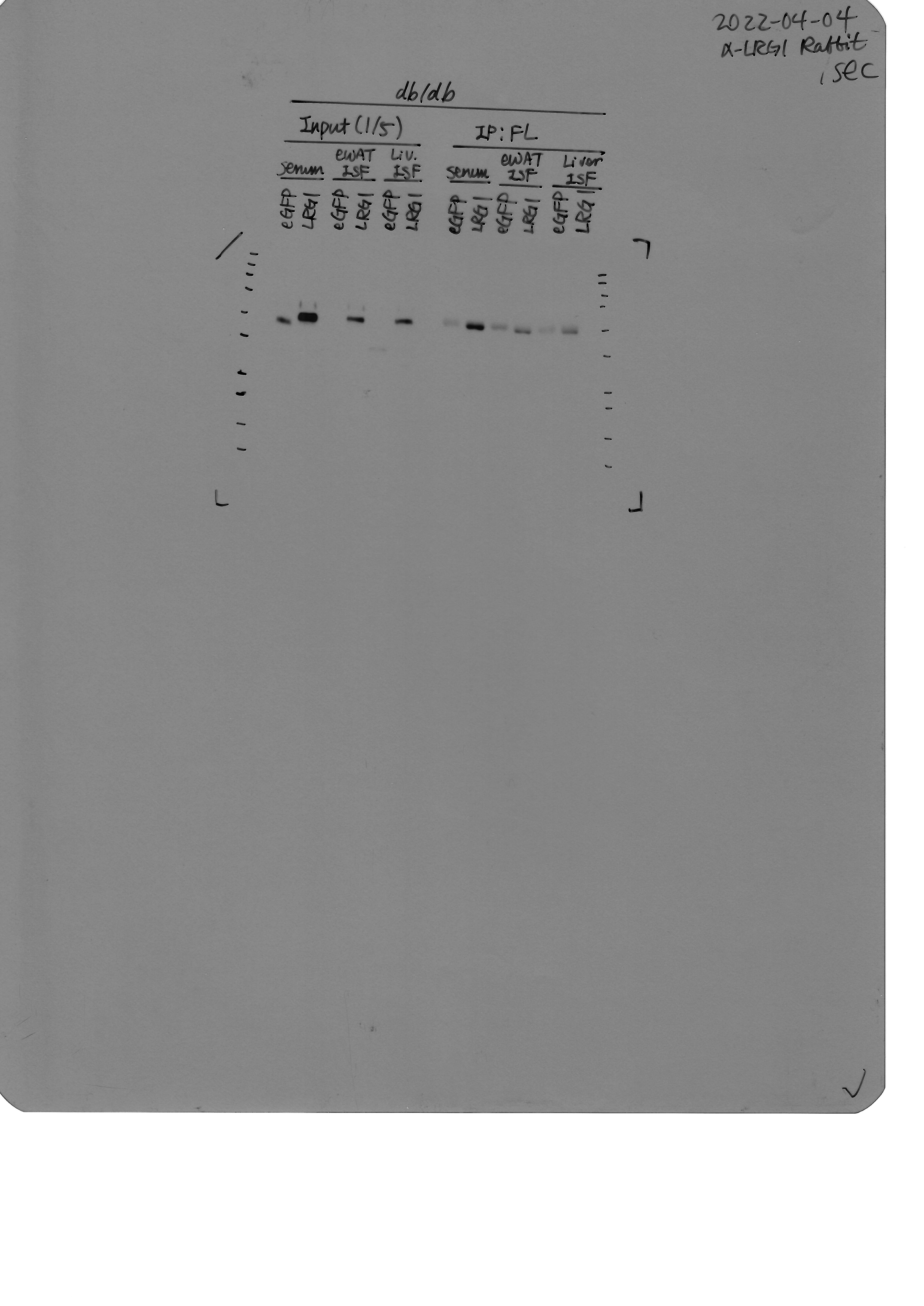

Supplement: Figure 7—source data 2. [file elife-81559-fig7-data2.zip › Figure 7-source data 2/db-db ISF a-LRG1.tif]

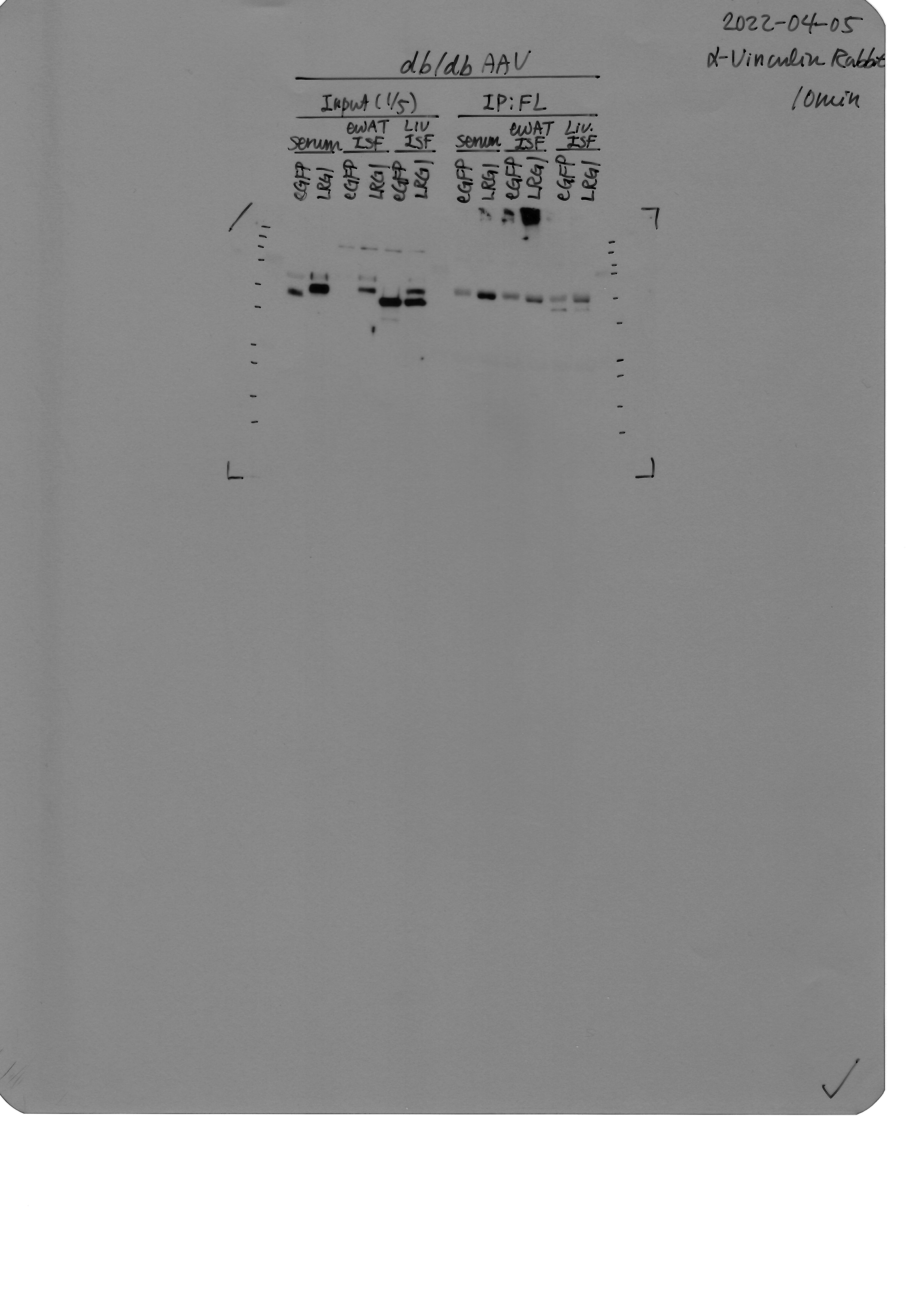

Supplement: Figure 7—source data 2. [file elife-81559-fig7-data2.zip › Figure 7-source data 2/db-db ISF a-Vinculin.tif]

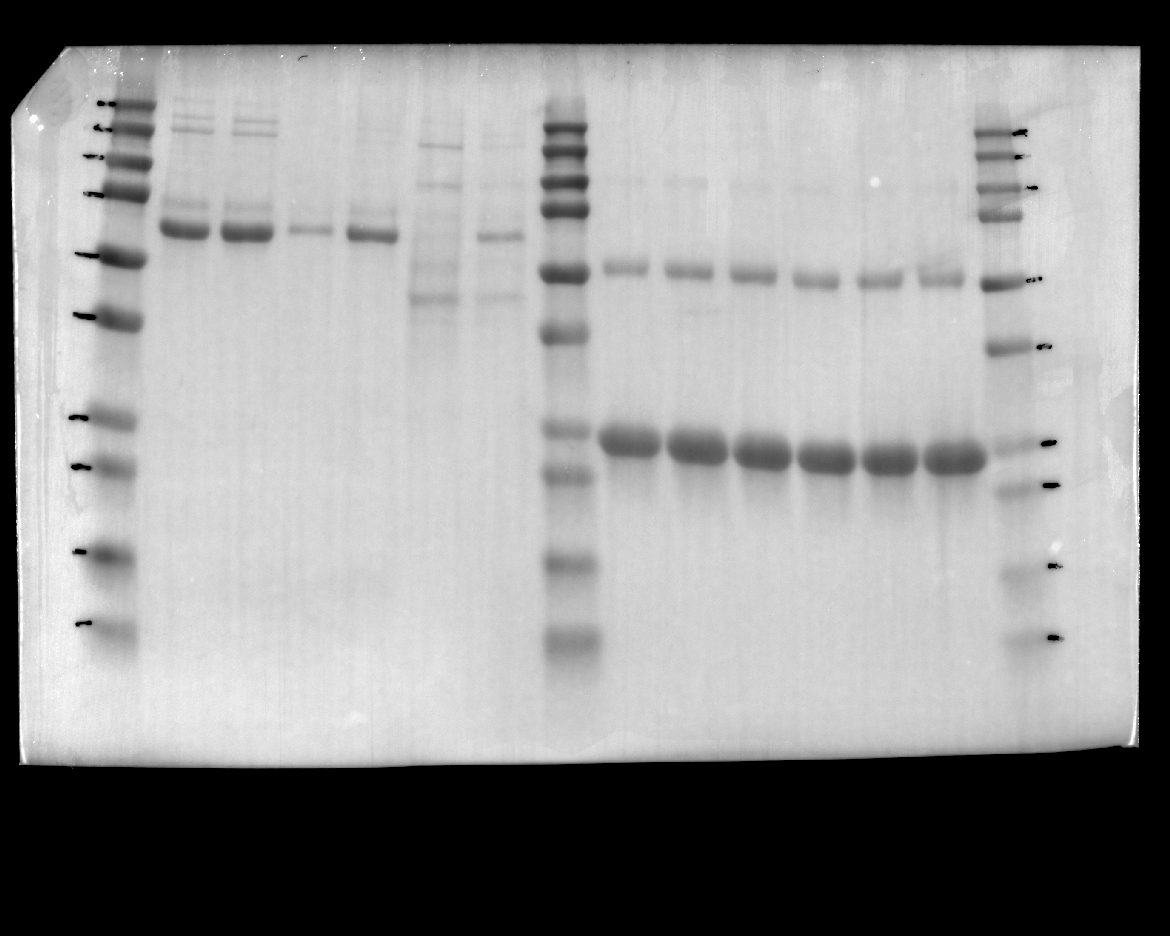

Supplement: Figure 7—source data 2. [file elife-81559-fig7-data2.zip › Figure 7-source data 2/db-db ISF Ponceau S.tif]

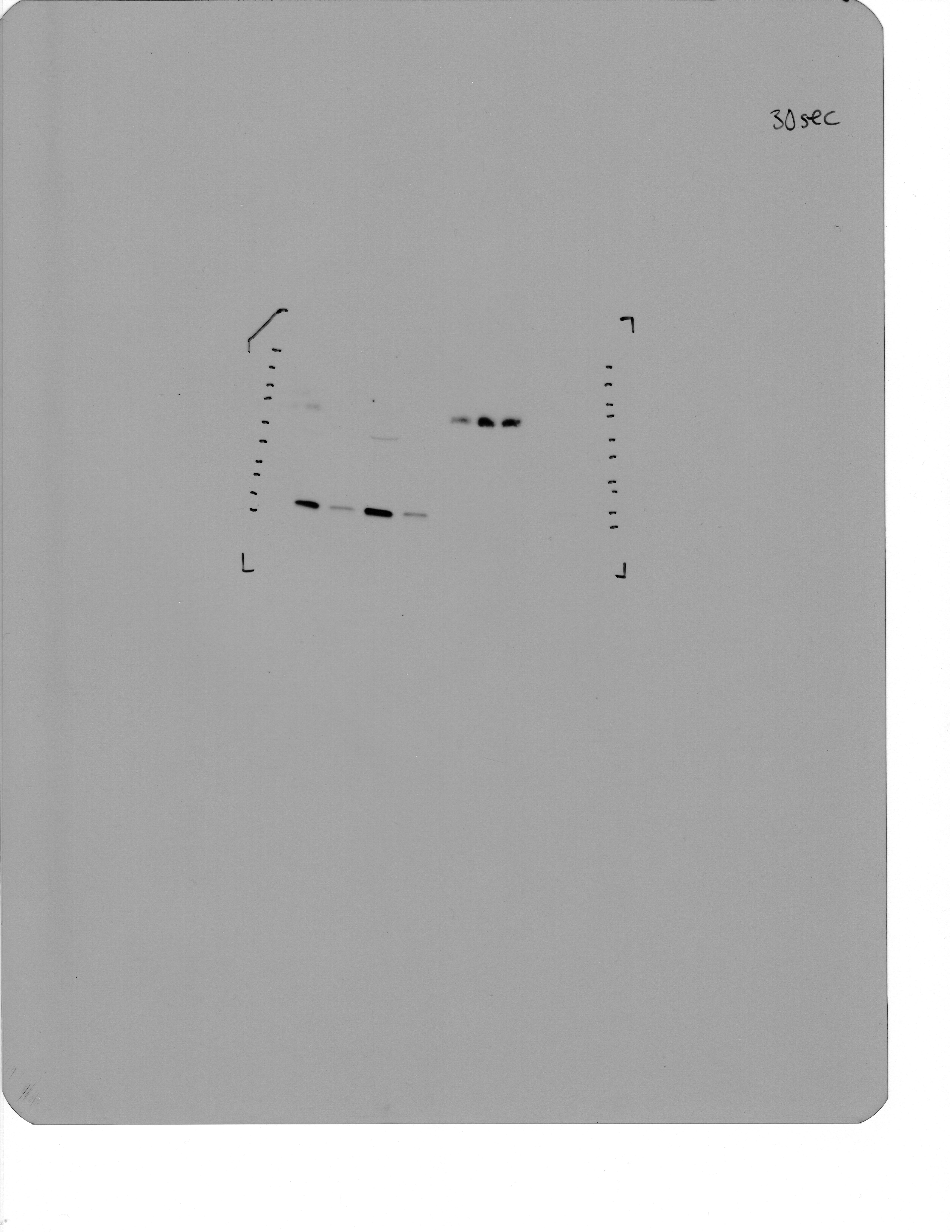

Supplement: Figure 7—source data 2. [file elife-81559-fig7-data2.zip › Figure 7-source data 2/db-db serum FLAG-IP a-Cyt c 30sec.jpg]

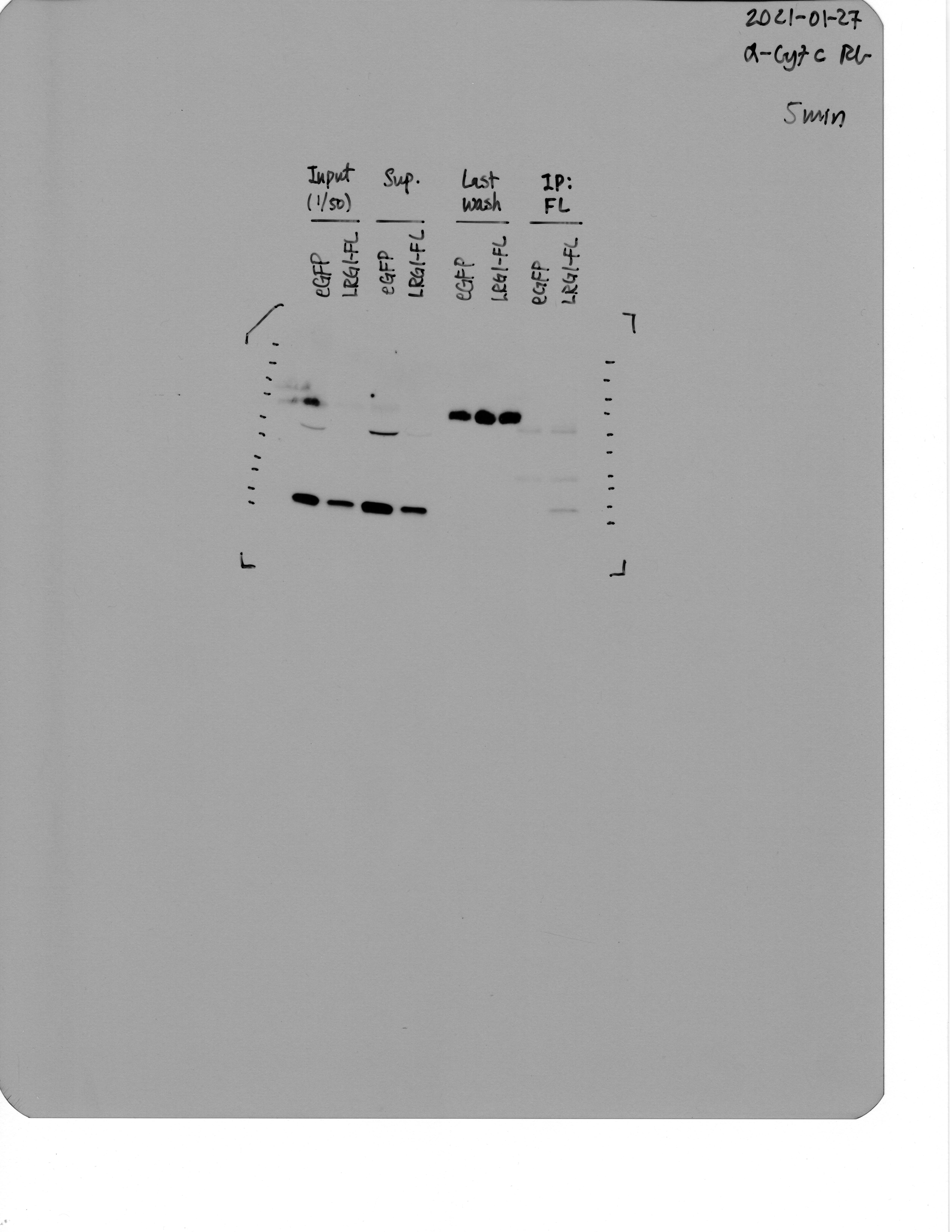

Supplement: Figure 7—source data 2. [file elife-81559-fig7-data2.zip › Figure 7-source data 2/db-db serum FLAG-IP a-Cyt c 5min.jpg]

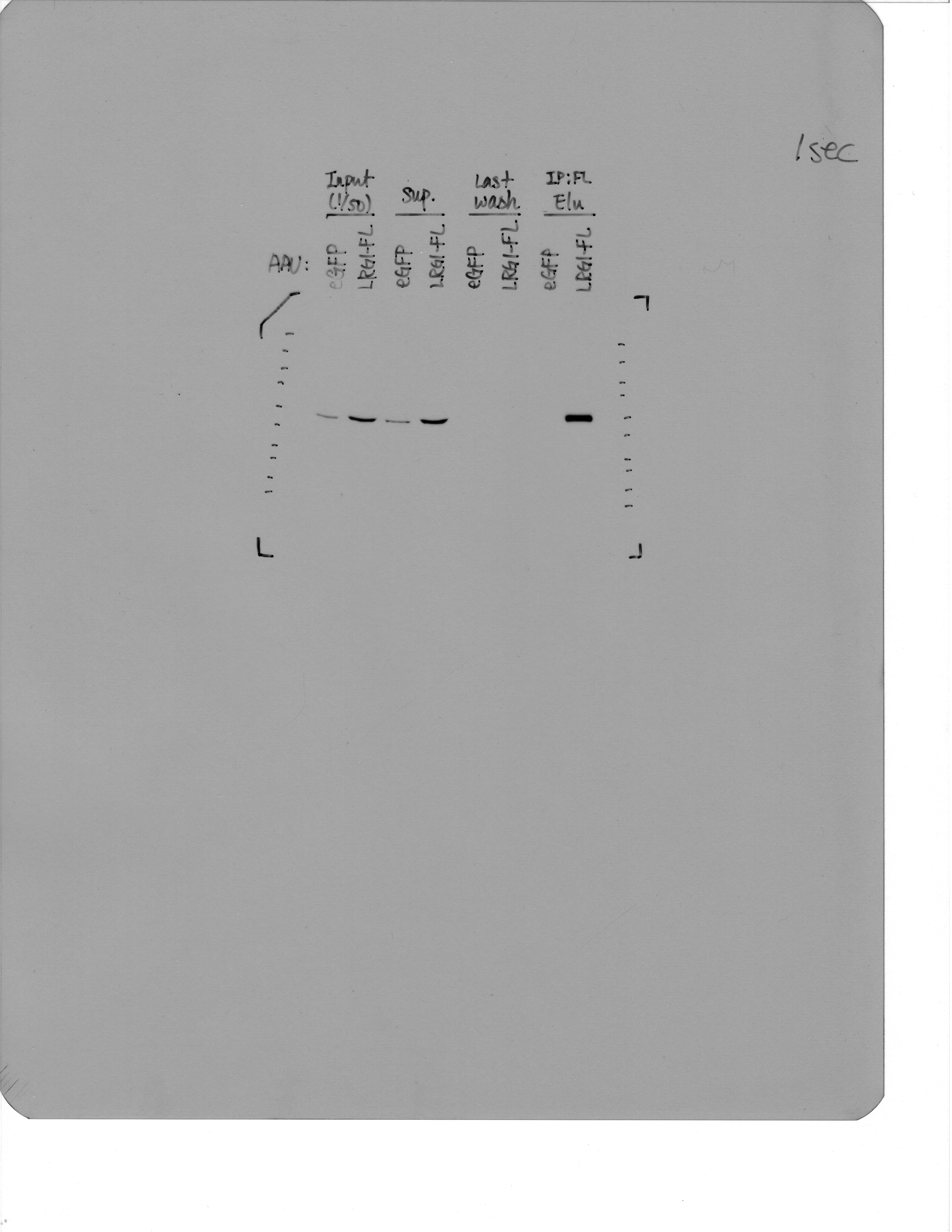

Supplement: Figure 7—source data 2. [file elife-81559-fig7-data2.zip › Figure 7-source data 2/db-db serum FLAG-IP a-LRG1 1sec.jpg]

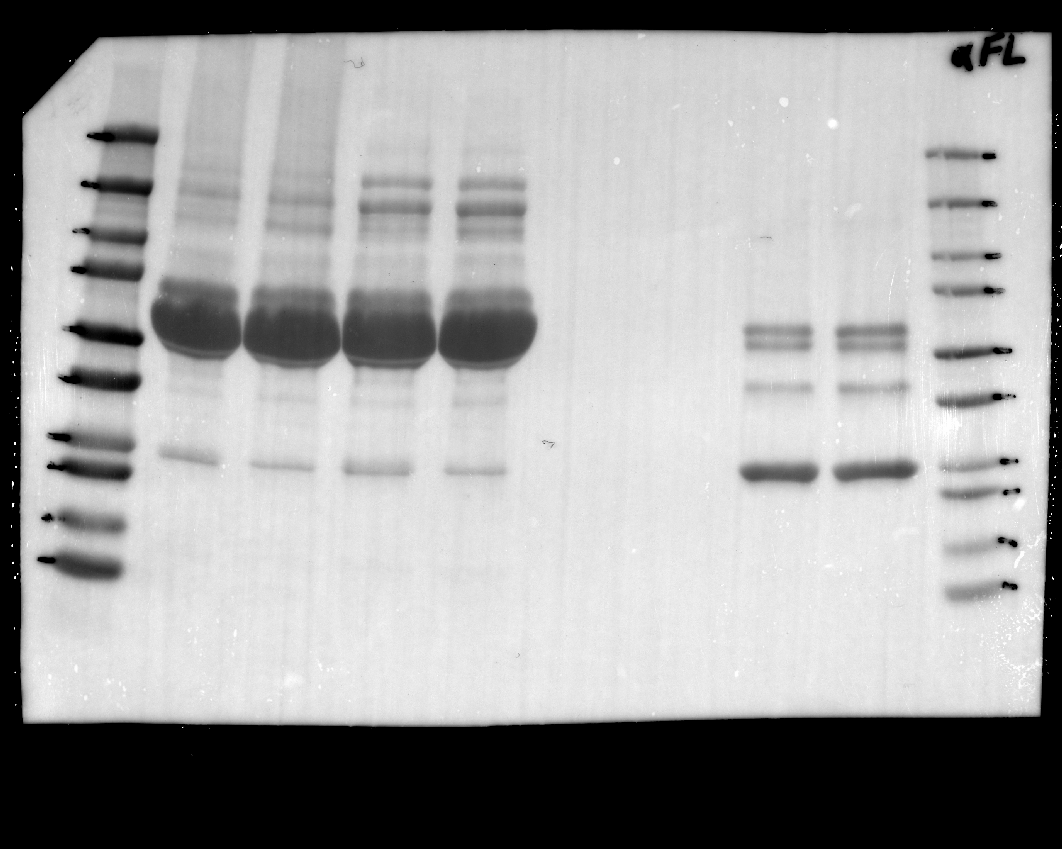

Supplement: Figure 7—source data 2. [file elife-81559-fig7-data2.zip › Figure 7-source data 2/db-db serum FLAG-IP Ponceau S.tif]

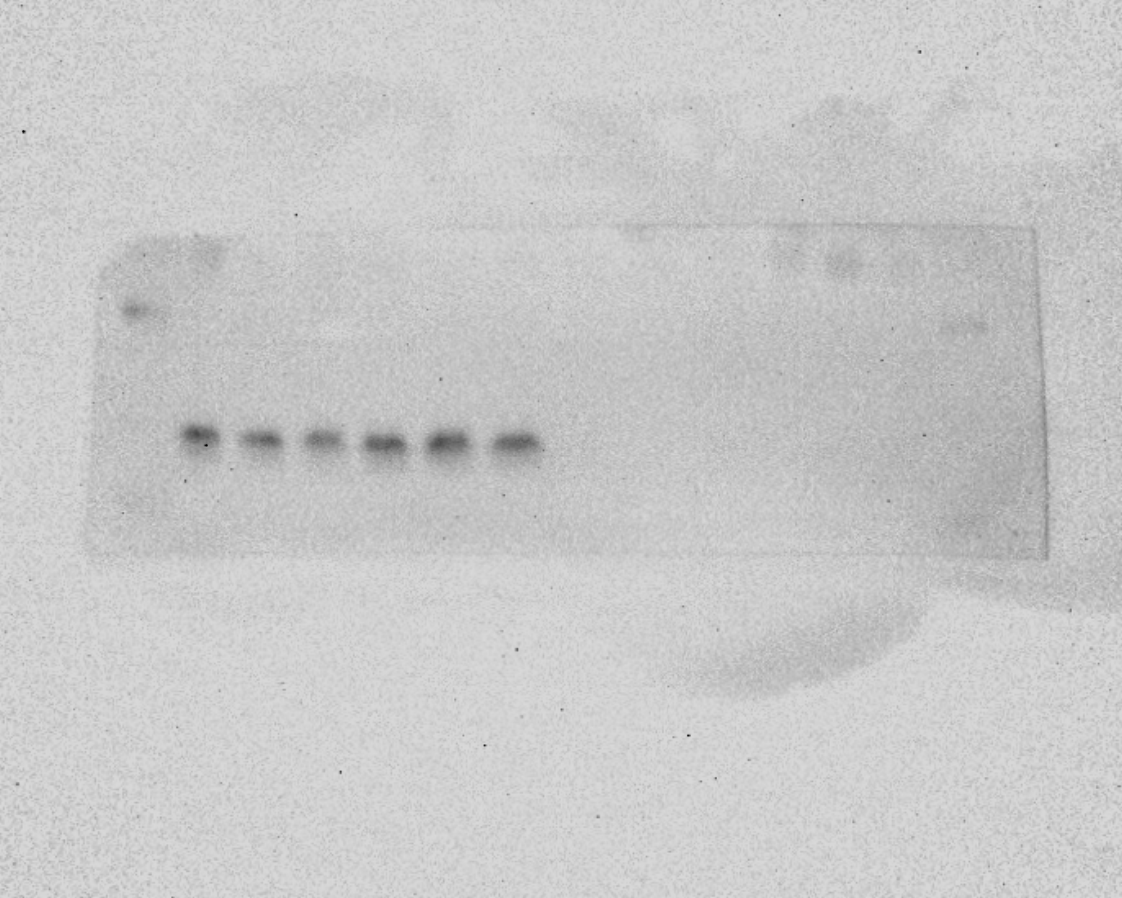

Supplement: Figure 7—source data 2. [file elife-81559-fig7-data2.zip › Figure 7-source data 2/Figure 7-S1/LRG1-KO vs. WT HFD Serum Cyt c.tif]

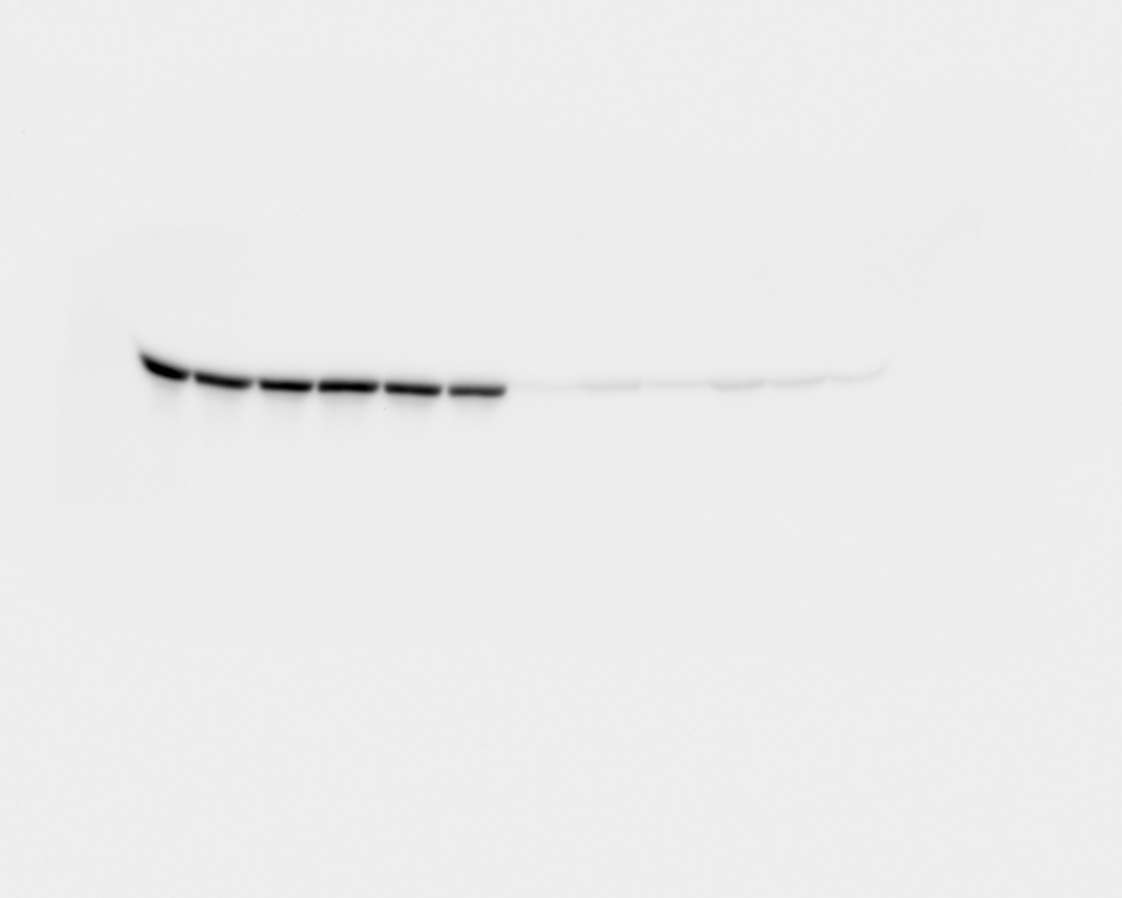

Supplement: Figure 7—source data 2. [file elife-81559-fig7-data2.zip › Figure 7-source data 2/Figure 7-S1/LRG1-KO vs. WT HFD Serum LRG1.tif]

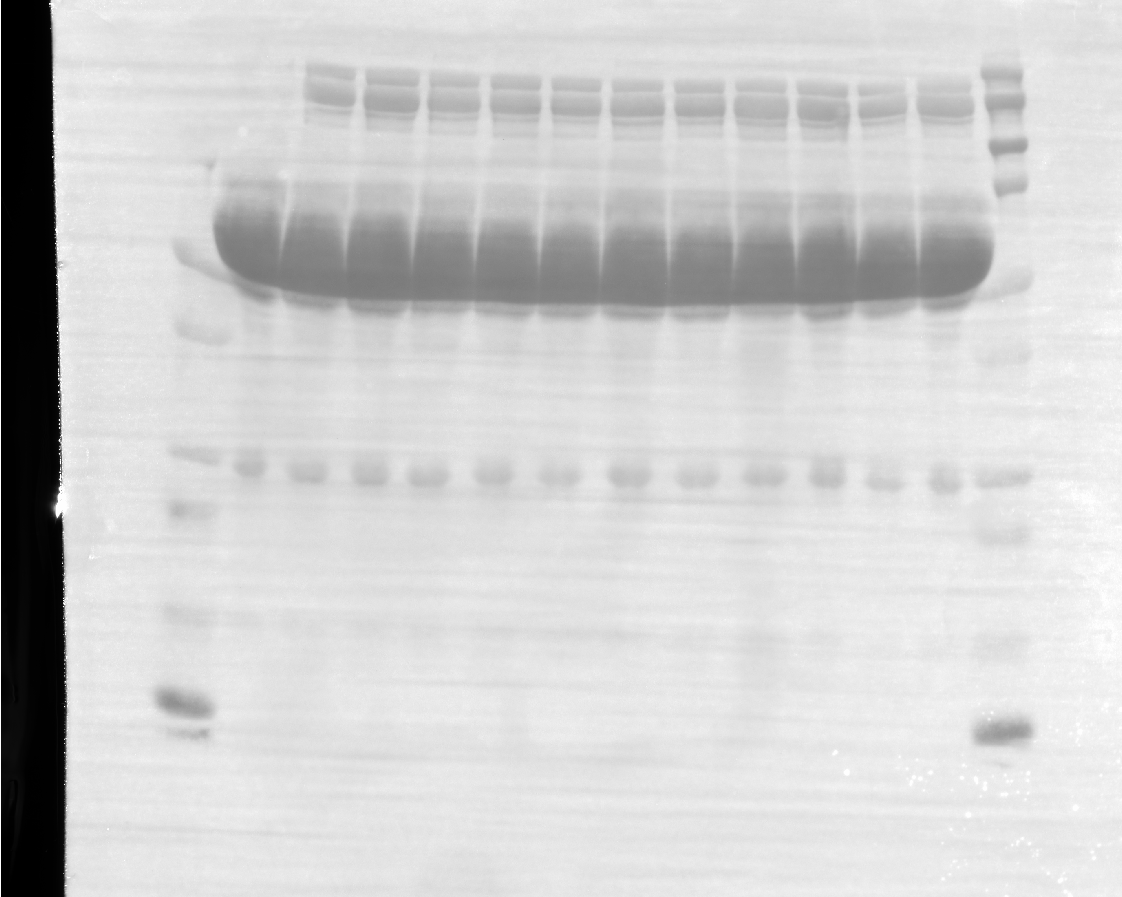

Supplement: Figure 7—source data 2. [file elife-81559-fig7-data2.zip › Figure 7-source data 2/Figure 7-S1/LRG1-KO vs. WT HFD Serum Ponceau S.tif]

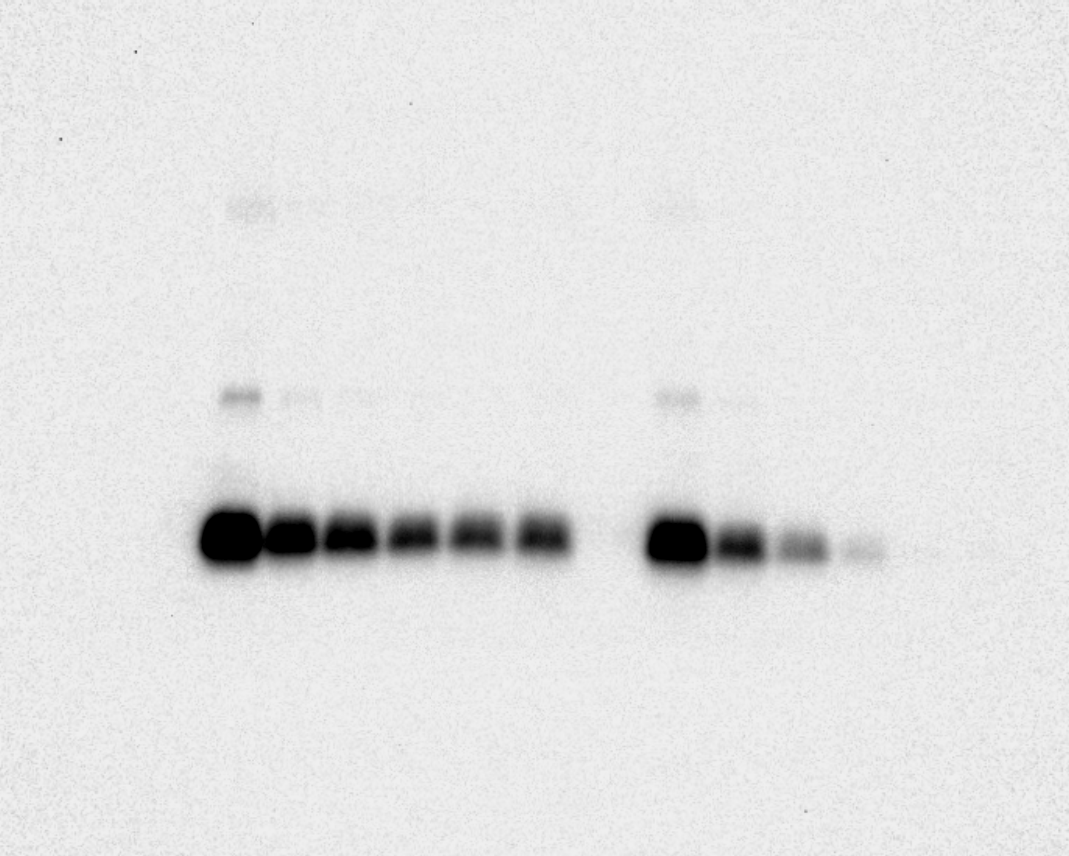

Supplement: Figure 7—source data 2. [file elife-81559-fig7-data2.zip › Figure 7-source data 2/Figure 7-S1/WT and LRG1-KO Cyt c clearance a-Cyt c.tif]

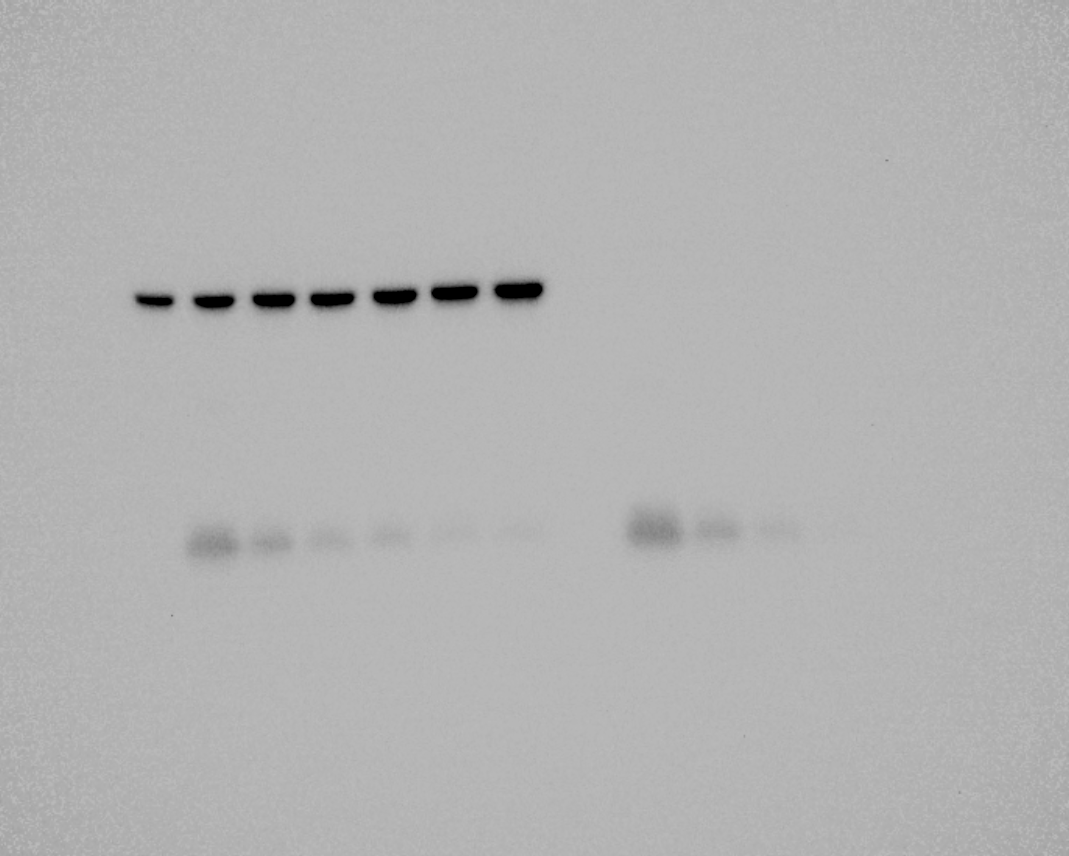

Supplement: Figure 7—source data 2. [file elife-81559-fig7-data2.zip › Figure 7-source data 2/Figure 7-S1/WT and LRG1-KO Cyt c clearance a-LRG1.tif]

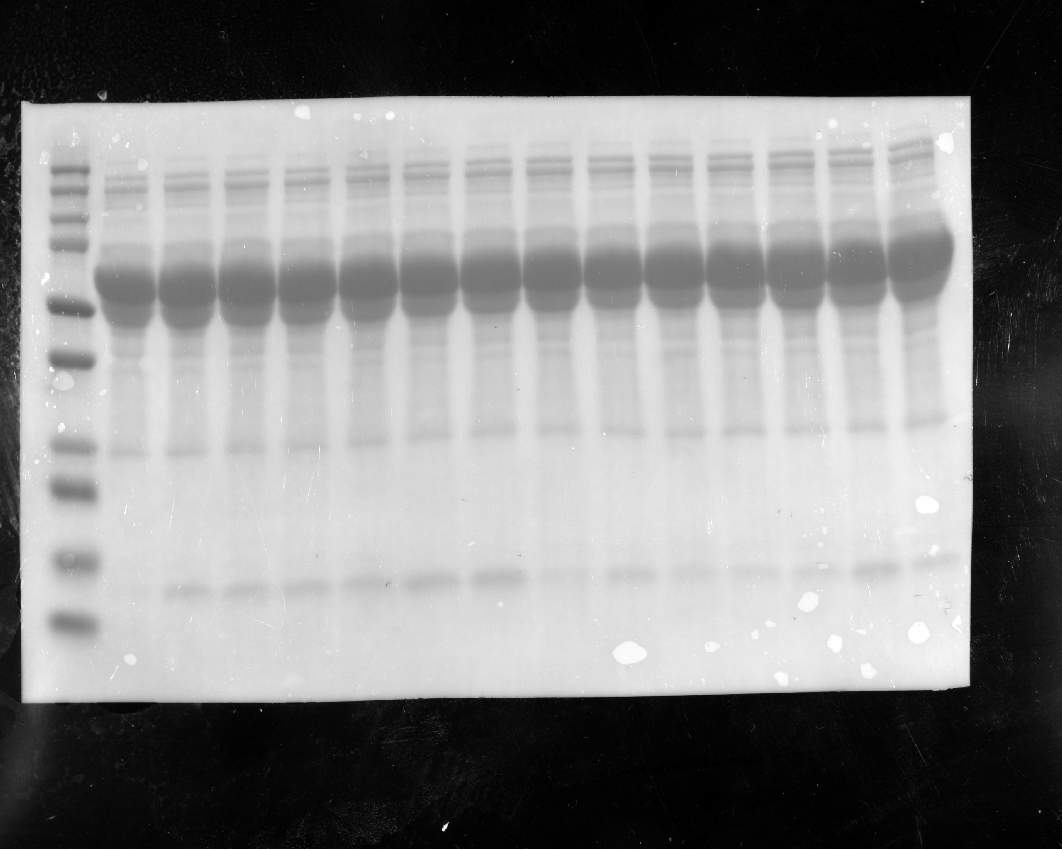

Supplement: Figure 7—source data 2. [file elife-81559-fig7-data2.zip › Figure 7-source data 2/Figure 7-S1/WT and LRG1-KO Cyt c clearance Ponceau S.tif]

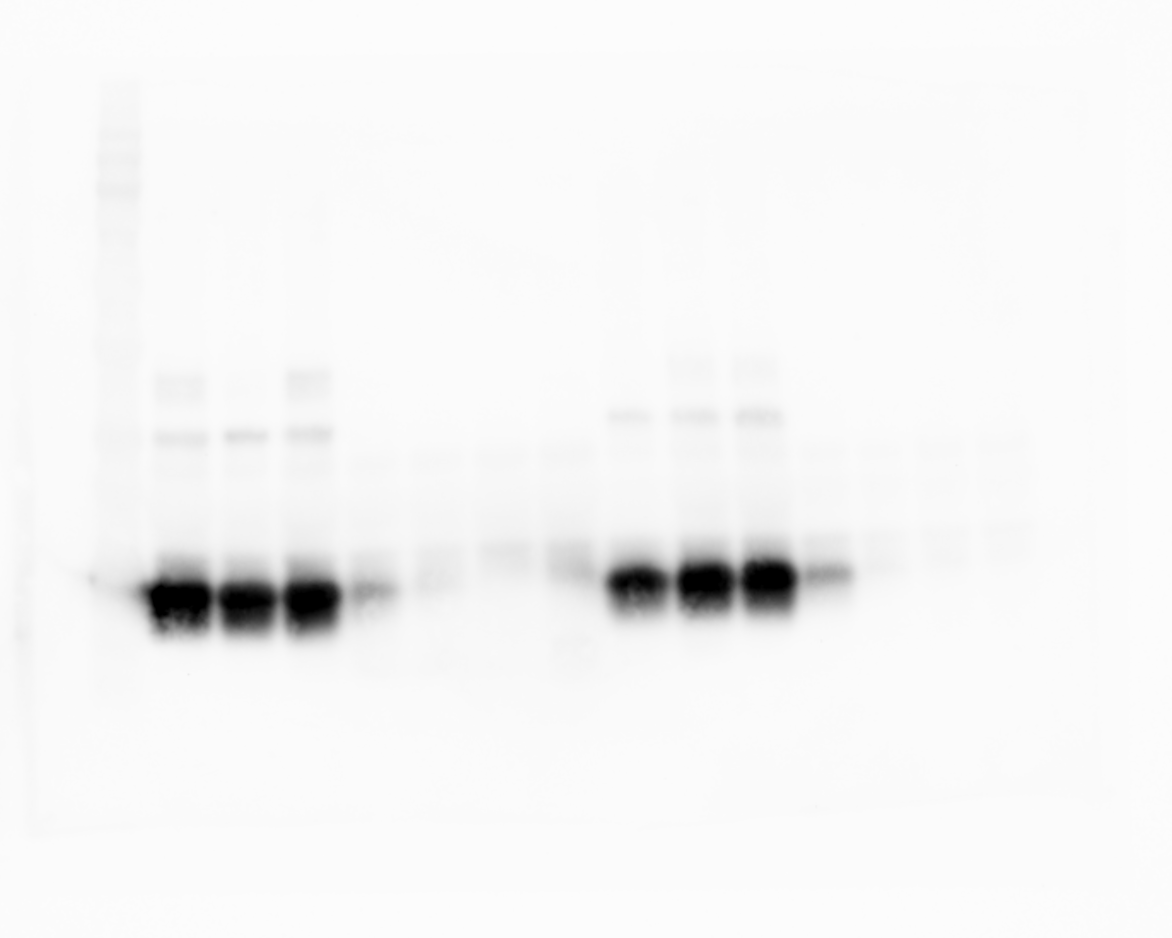

Supplement: Figure 7—source data 2. [file elife-81559-fig7-data2.zip › Figure 7-source data 2/Figure 7-S1/WT and LRG1-KO Urine a-Cyt c.tif]

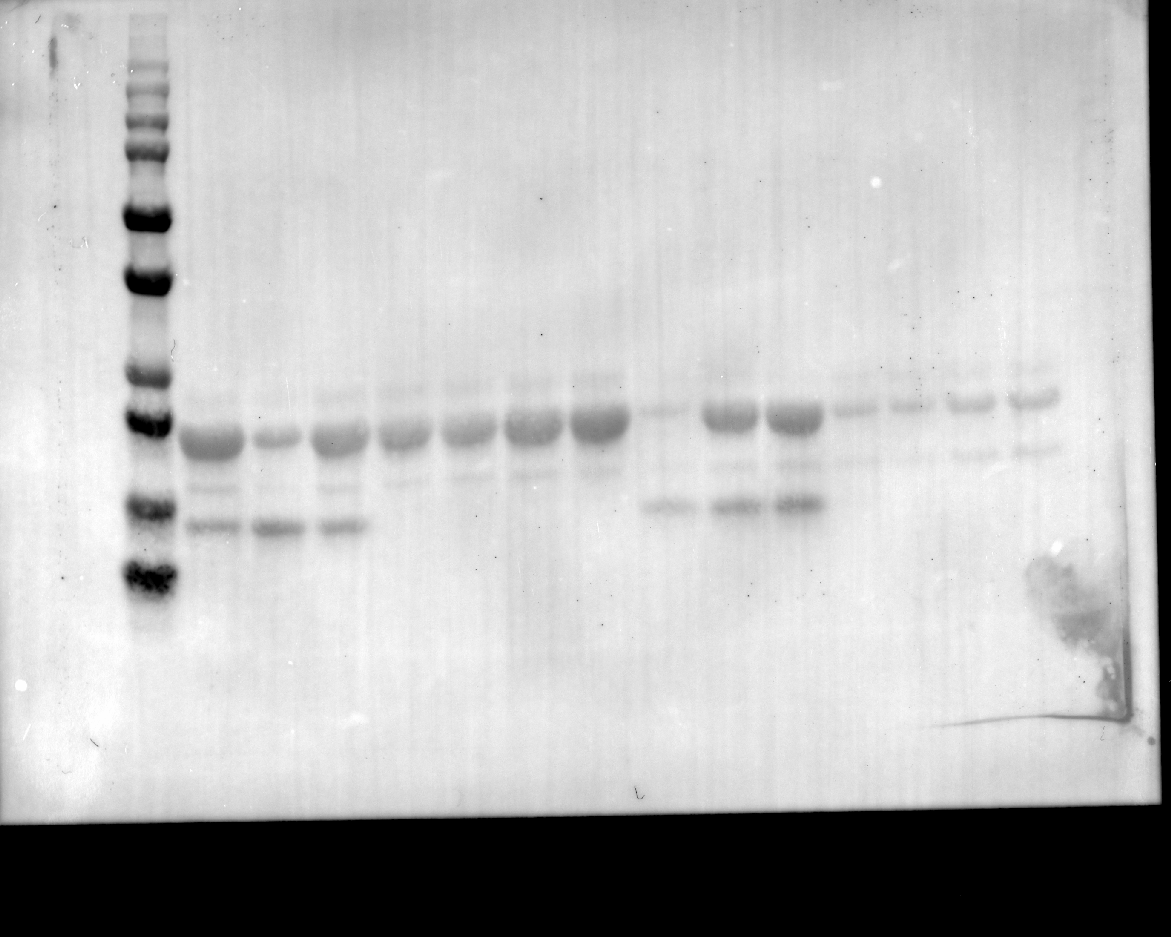

Supplement: Figure 7—source data 2. [file elife-81559-fig7-data2.zip › Figure 7-source data 2/Figure 7-S1/WT and LRG1-KO Urine Ponceau S.tif]

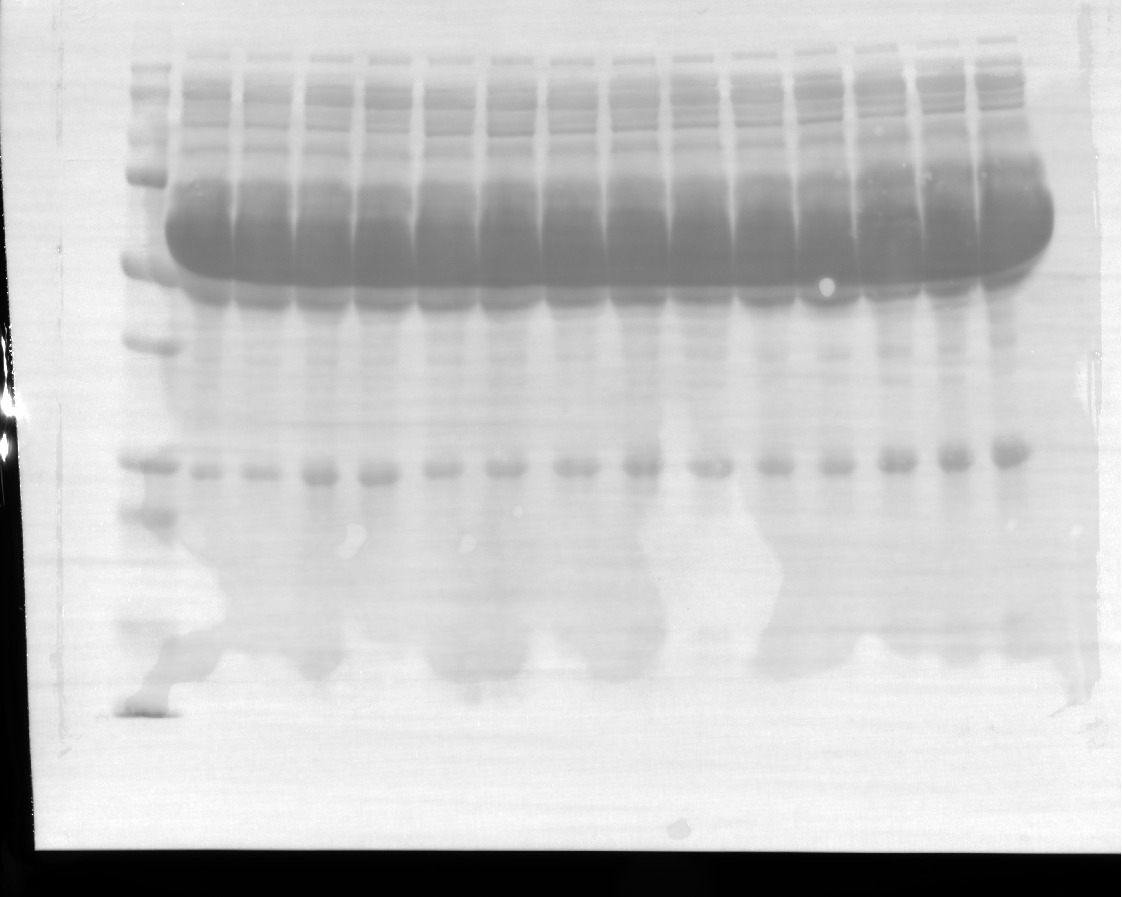

Supplement: Figure 7—source data 2. [file elife-81559-fig7-data2.zip › Figure 7-source data 2/Figure 7-S1/WT HFD Timecourse Serum Cyt c Ponceau S.tif]

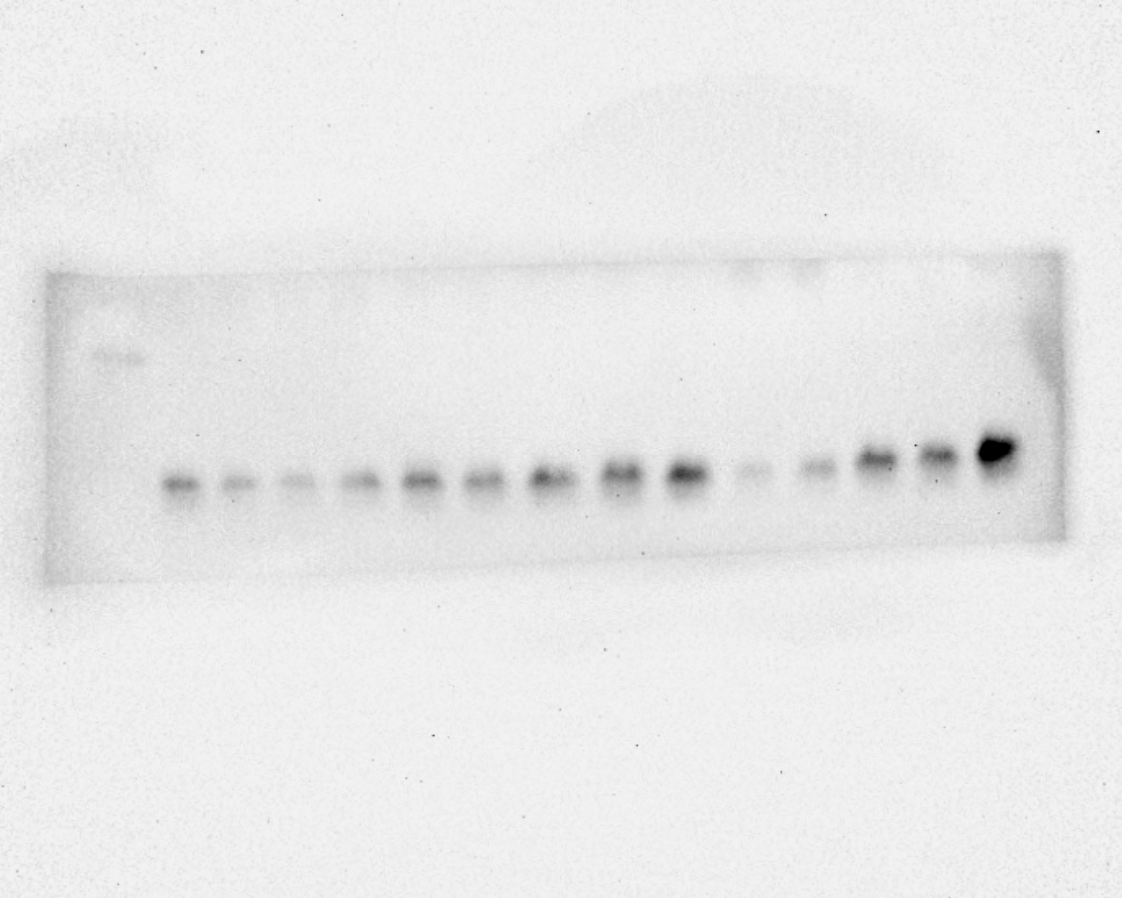

Supplement: Figure 7—source data 2. [file elife-81559-fig7-data2.zip › Figure 7-source data 2/Figure 7-S1/WT HFD Timecourse Serum Cyt c.tif]

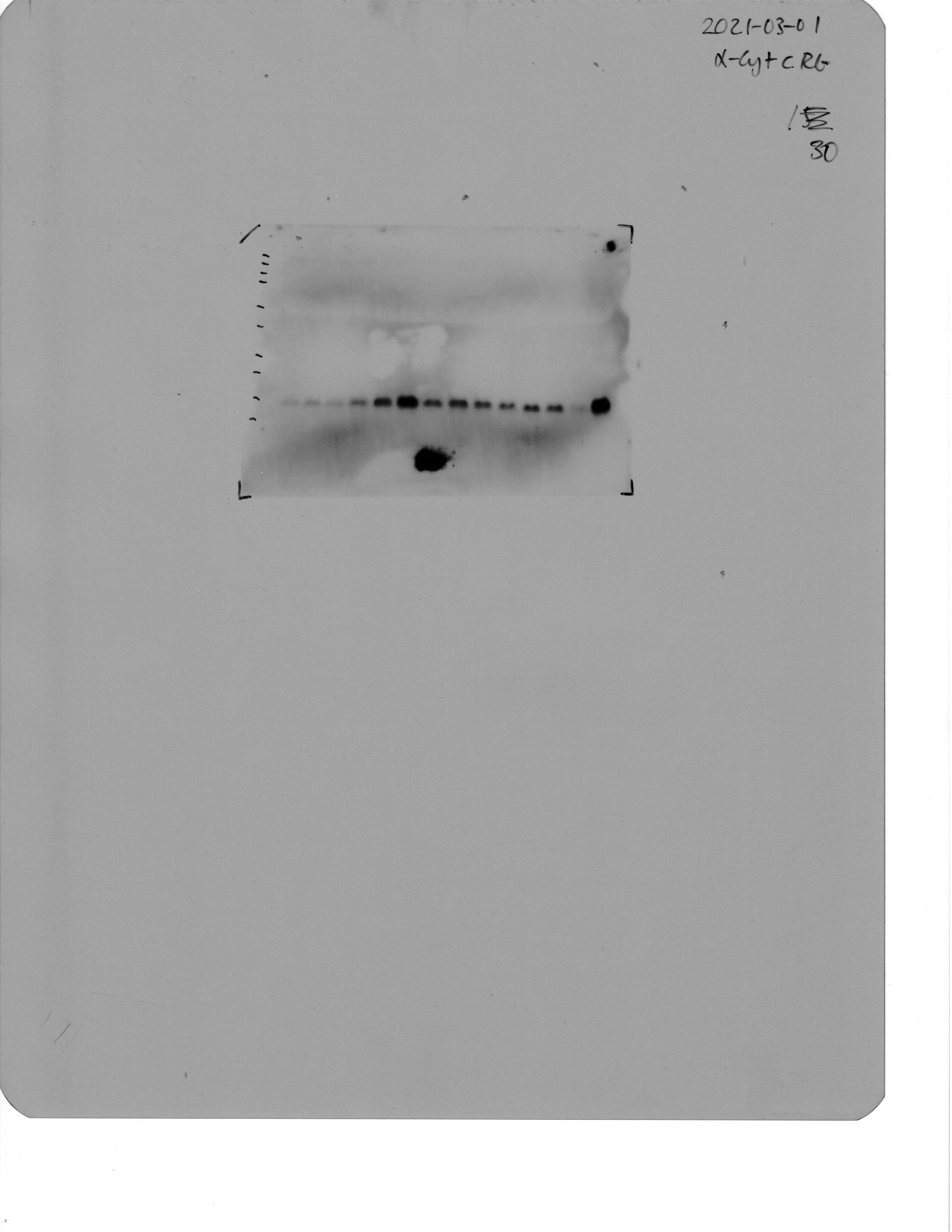

Supplement: Figure 7—source data 2. [file elife-81559-fig7-data2.zip › Figure 7-source data 2/m-m vs db-db Serum a-Cyt c 30sec.jpg]

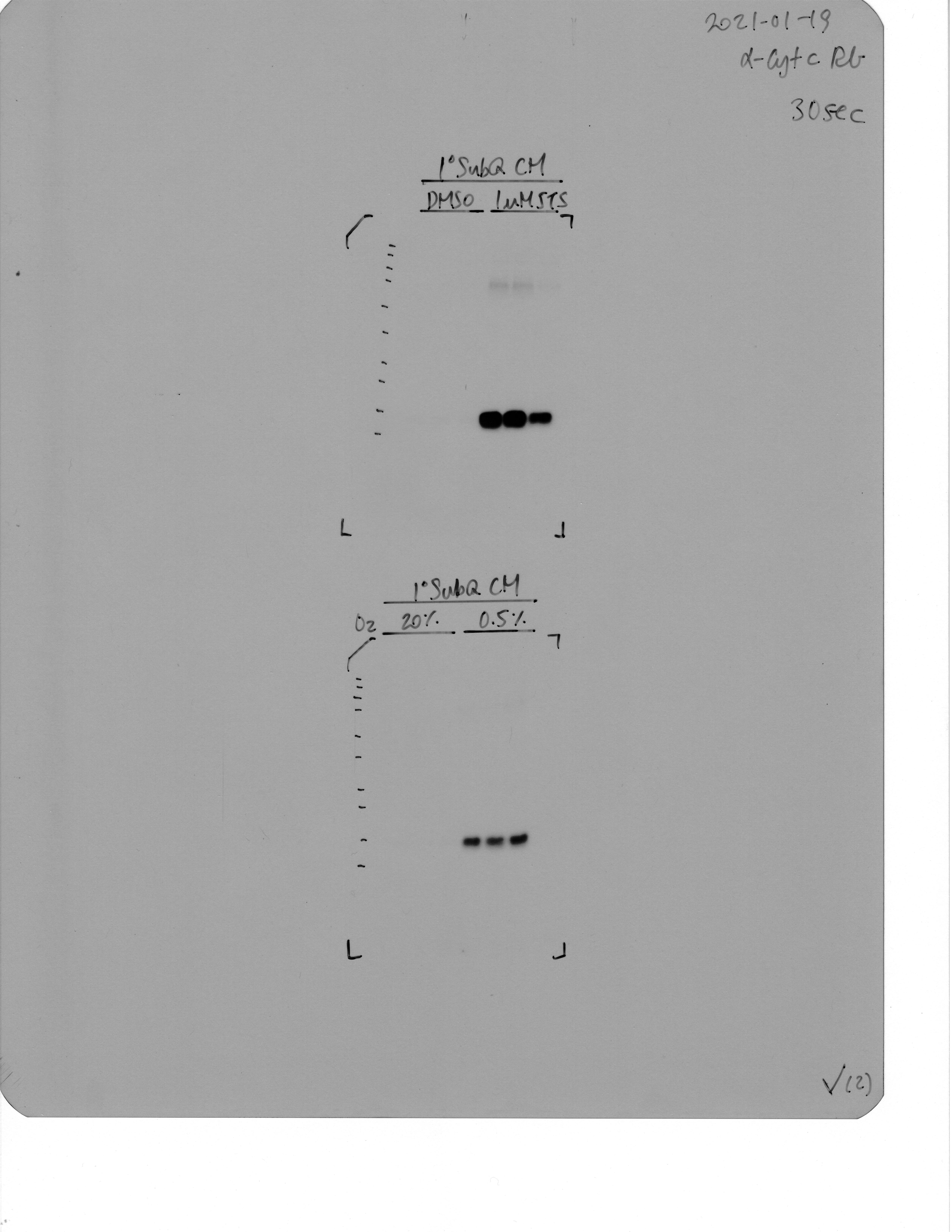

Supplement: Figure 7—source data 2. [file elife-81559-fig7-data2.zip › Figure 7-source data 2/SubQ CM Hypoxia a-Cyt c 30sec.jpg]

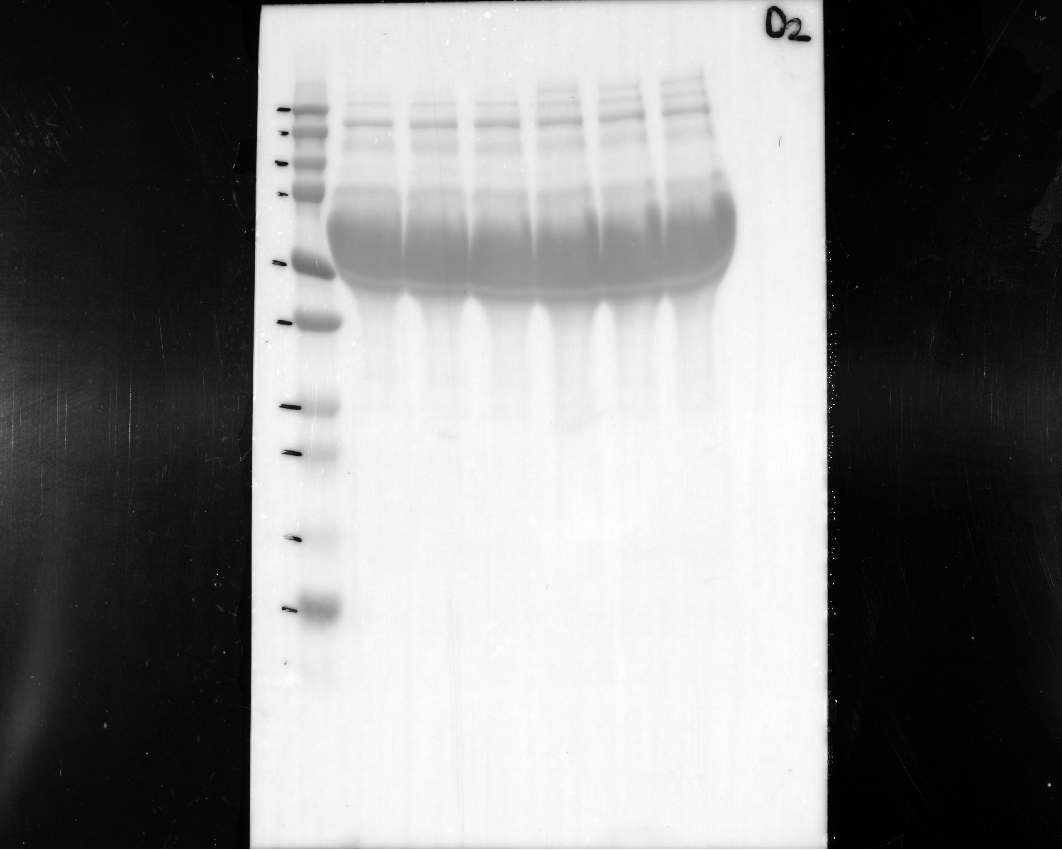

Supplement: Figure 7—source data 2. [file elife-81559-fig7-data2.zip › Figure 7-source data 2/SubQ CM Hypoxia Ponceau S.tif]

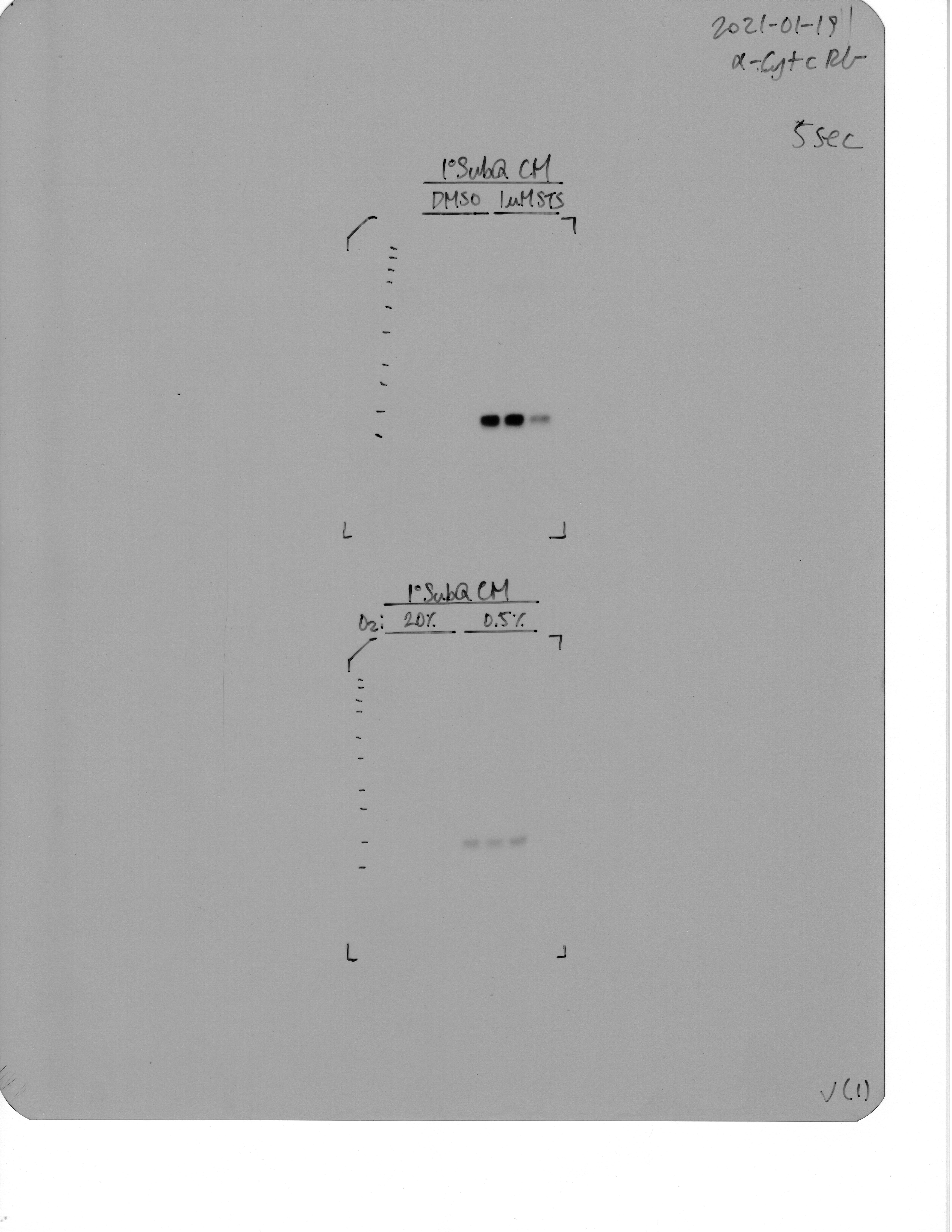

Supplement: Figure 7—source data 2. [file elife-81559-fig7-data2.zip › Figure 7-source data 2/SubQ CM STS a-Cyt c 5sec.jpg]

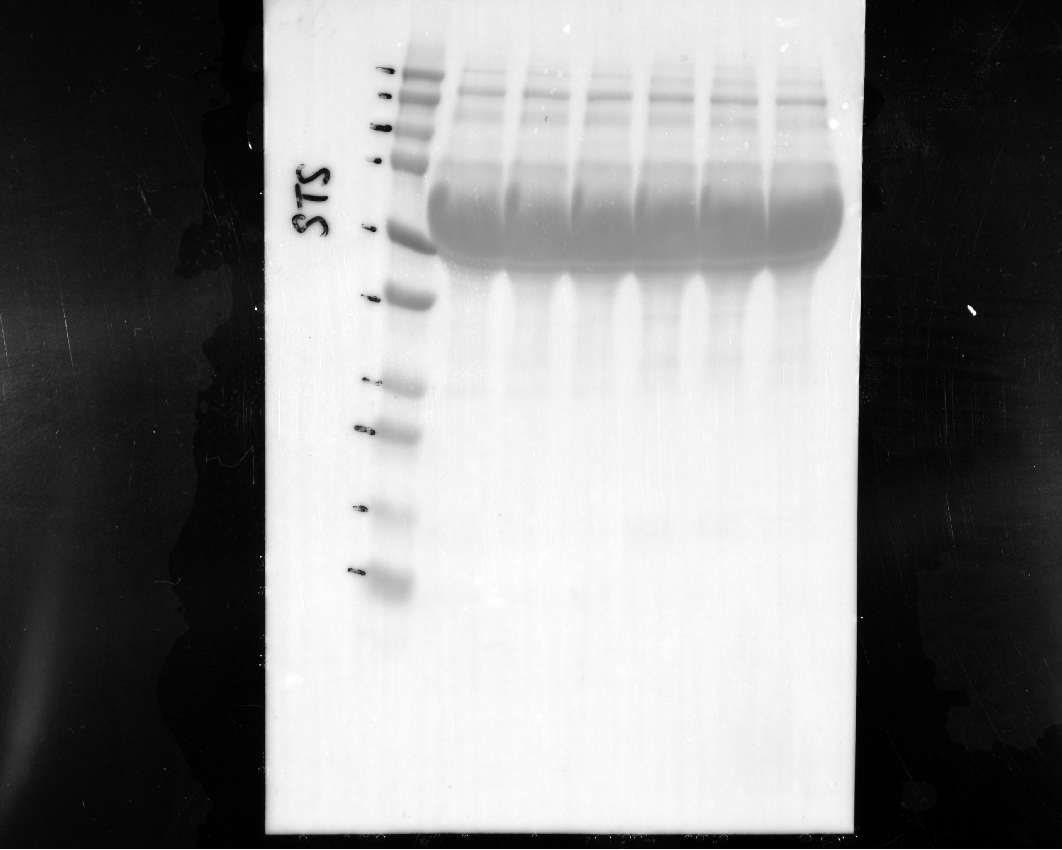

Supplement: Figure 7—source data 2. [file elife-81559-fig7-data2.zip › Figure 7-source data 2/SubQ CM STS Ponceau S.tif]
