# Supplementary material for: LRG1 is an adipokine that promotes insulin sensitivity and suppresses inflammation
Source: eLife. 2022 Nov 8;11:e81559. doi: 10.7554/eLife.81559 (PMC9674348; doi:10.7554/eLife.81559)

Figure 4—source data 1

Figure 4B

LRG1

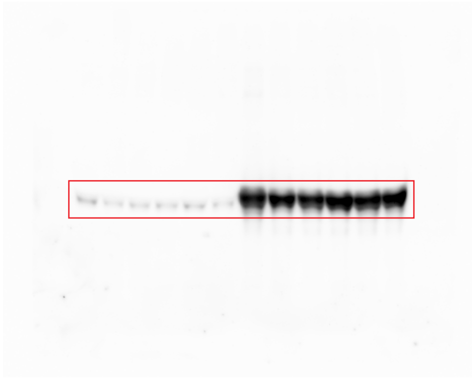

Ponceau S

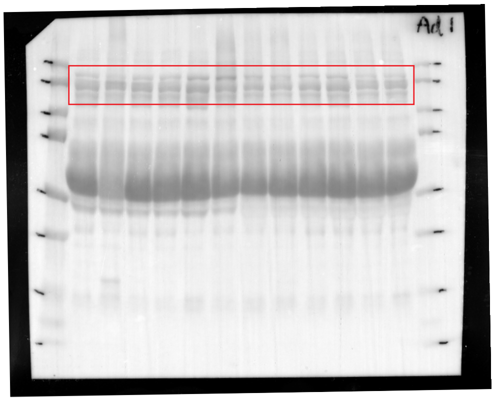

Figure 4G

LRG1

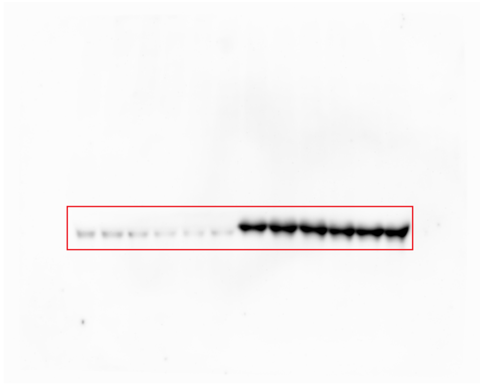

Ponceau S

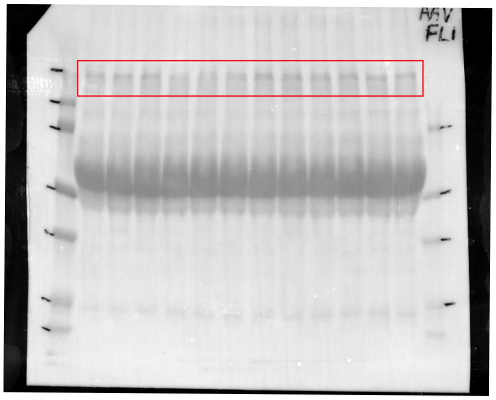

Figure 4I

LRG1

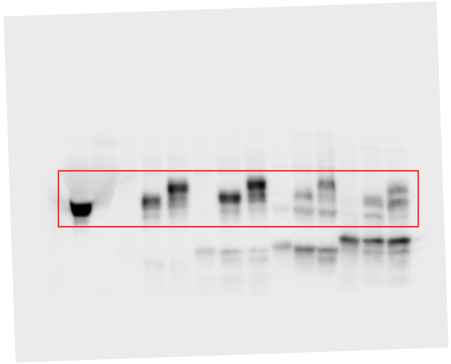

Vinculin

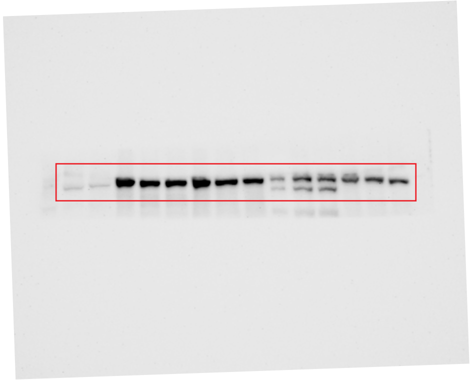

Ponceau S

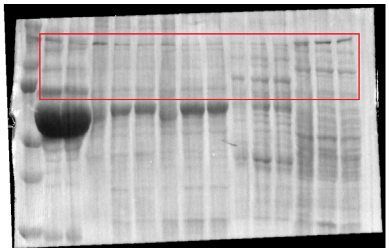

LRG1

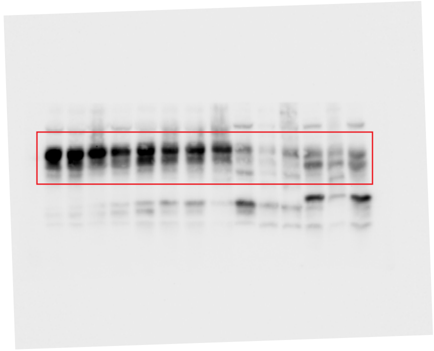

Vinculin

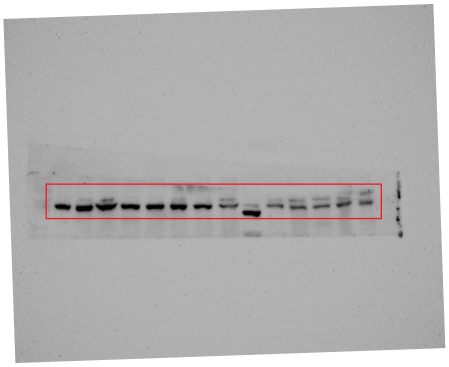

Ponceau S

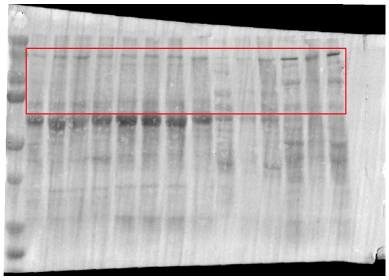

Figure 4—figure supplement 2C

LRG1

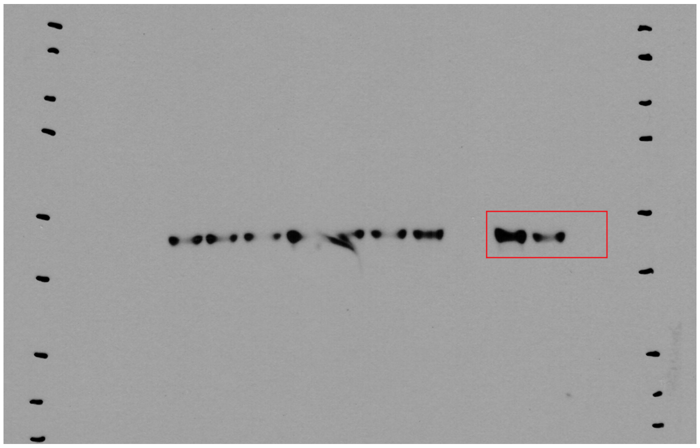

Ponceau S

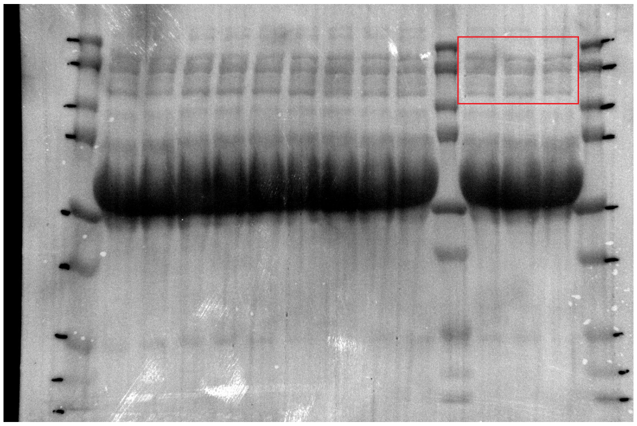

Supplement: Figure 4—source data 1. [file elife-81559-fig4-data1.pdf]
