# Supplementary material for: LRG1 is an adipokine that promotes insulin sensitivity and suppresses inflammation
Source: eLife. 2022 Nov 8;11:e81559. doi: 10.7554/eLife.81559 (PMC9674348; doi:10.7554/eLife.81559)

# Figure 5—source data 1

## Figure 5B

LRG1

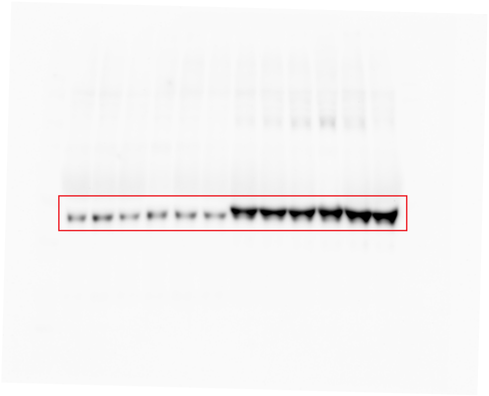

Ponceau S

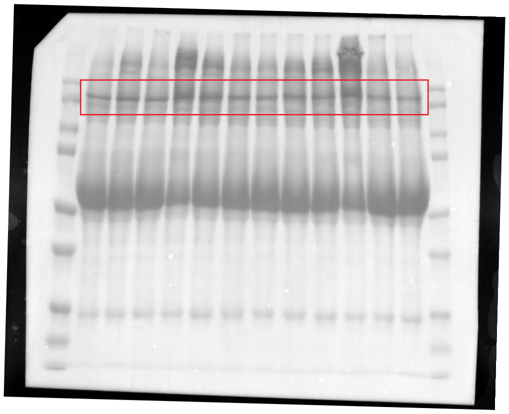

## Figure 5—figure supplement 1C

LRG1

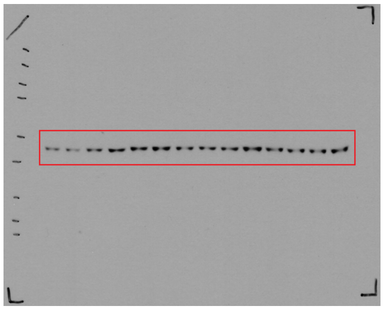

Ponceau S

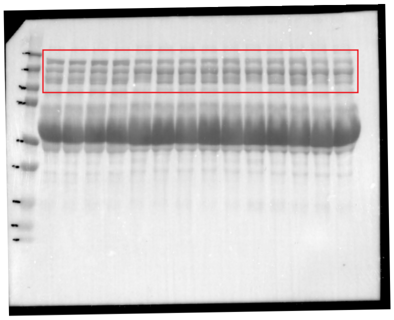

## Figure 5—figure supplement 1F

LRG1

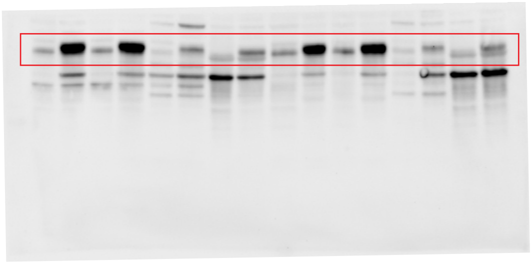

Vinculin

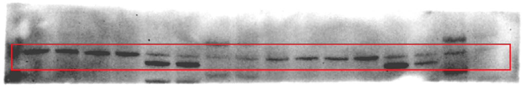

## Figure 5—figure supplement 1I

IRS-1

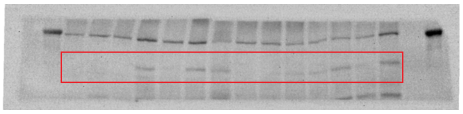

Vinculin

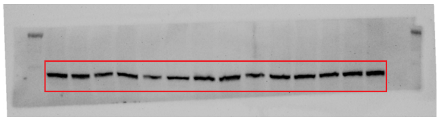

IRS-1

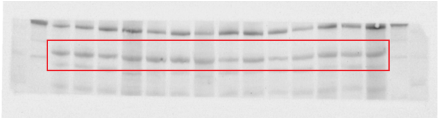

β-actin

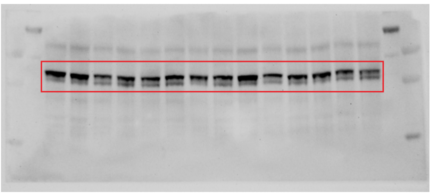

Supplement: Figure 5—source data 1. [file elife-81559-fig5-data1.pdf]
