# Supplementary material for: LRG1 is an adipokine that promotes insulin sensitivity and suppresses inflammation
Source: eLife. 2022 Nov 8;11:e81559. doi: 10.7554/eLife.81559 (PMC9674348; doi:10.7554/eLife.81559)

Figure 7—source data 1

Figure 7A

LRG1

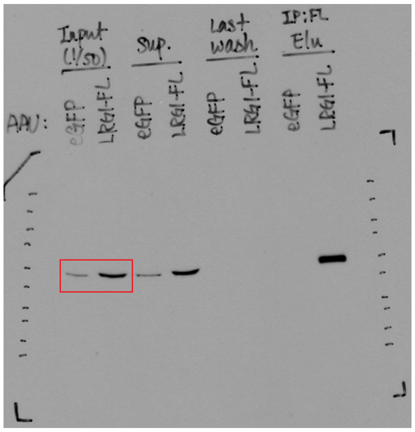

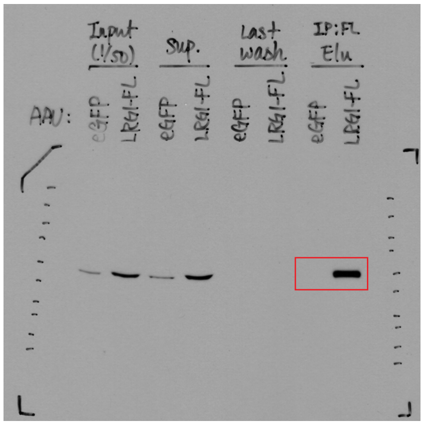

Cyt *c*

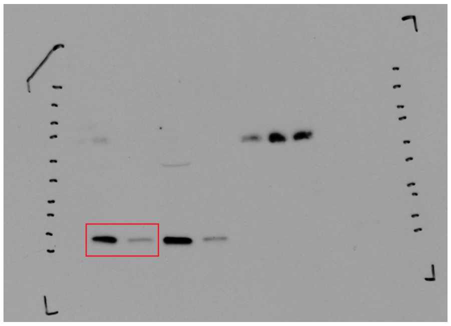

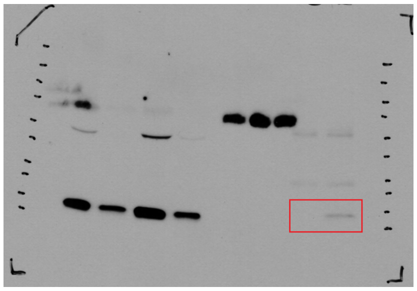

Ponceau S

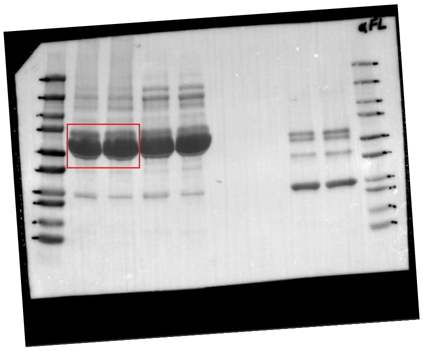

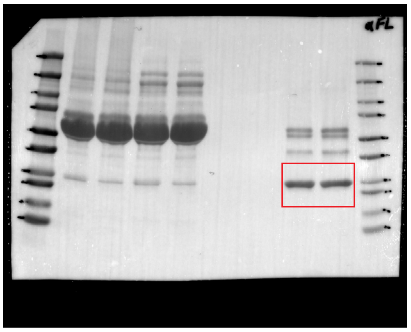

Figure 7B

Cyt *c*

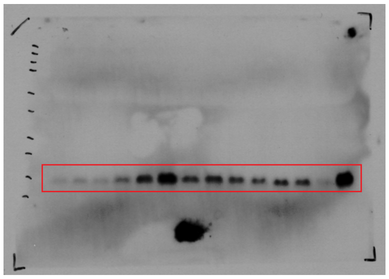

Ponceau S

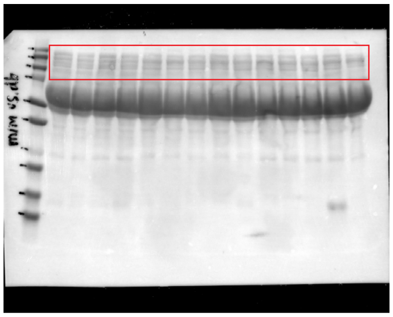

Figure 7D

Cyt *c*

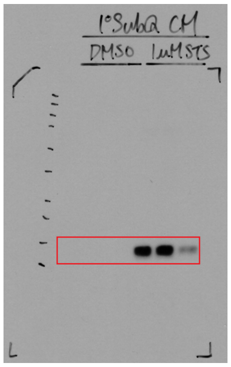

Ponceau S

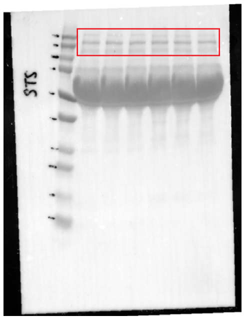

Cyt *c*

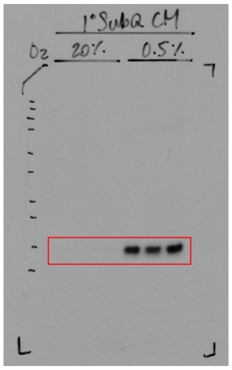

Ponceau S

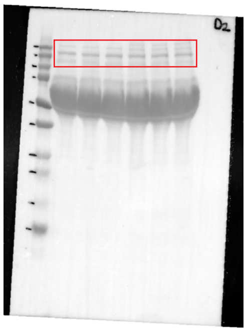

Figure 7E

Cyt *c*

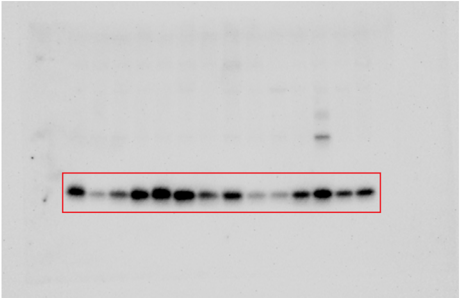

Ponceau S

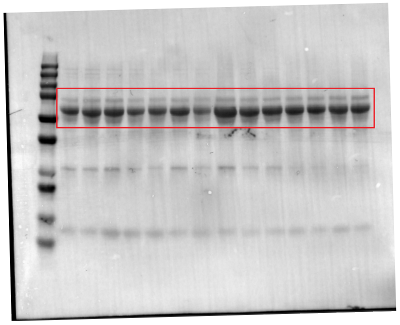

Cyt *c*

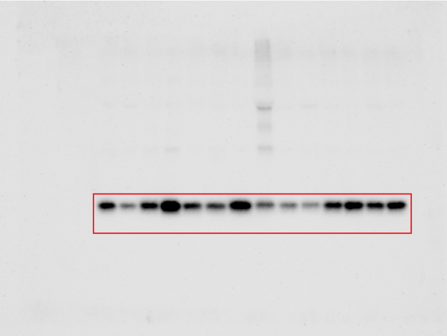

Ponceau S

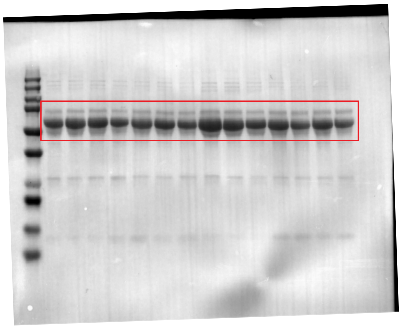

Figure 7H

Caspase-3

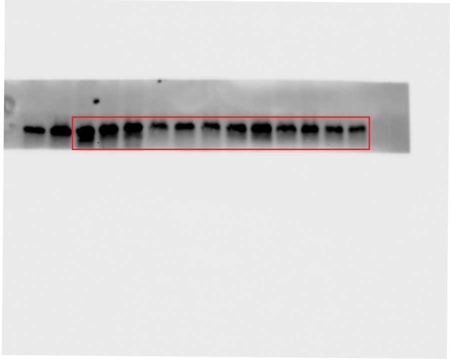

Cleaved Caspase-3

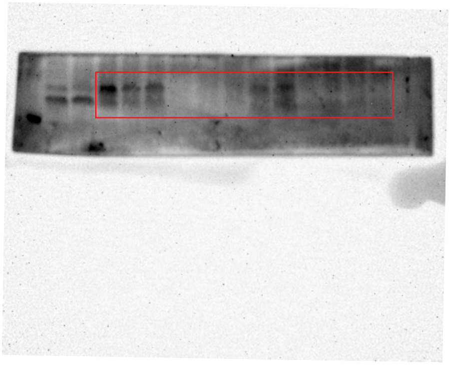

Vinculin

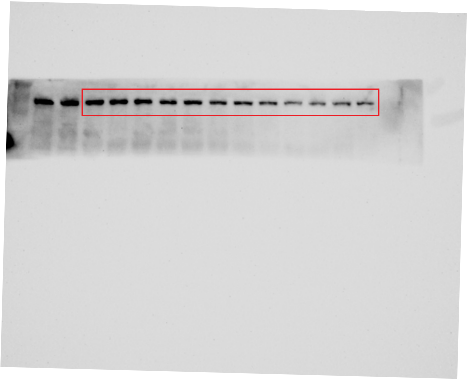

Figure 7I

LRG1

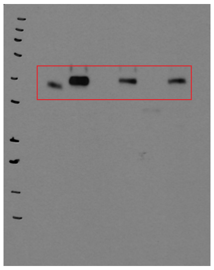

Cyt *c*

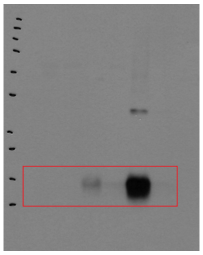

Vinculin

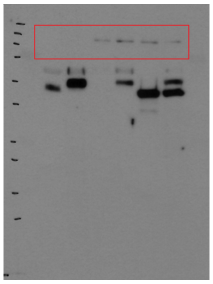

Ponc.S

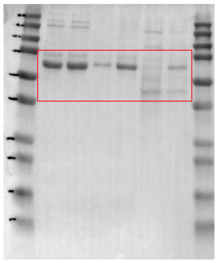

Figure 7—figure supplement 1C

Cyt *c*

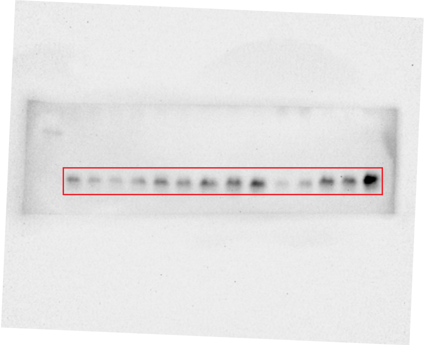

Ponceau S

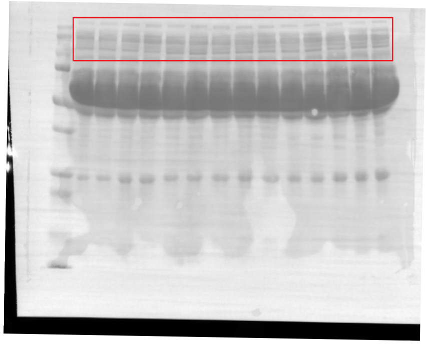

Figure 7—figure supplement 1I

Cyt *c*

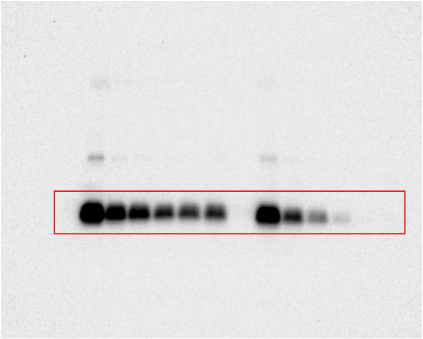

LRG1

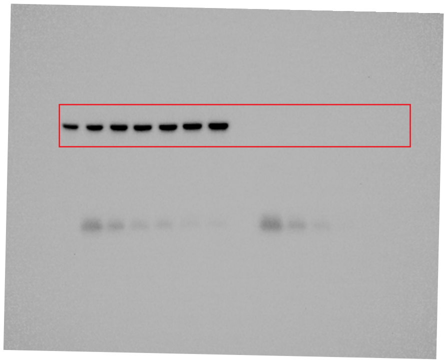

Ponceau S

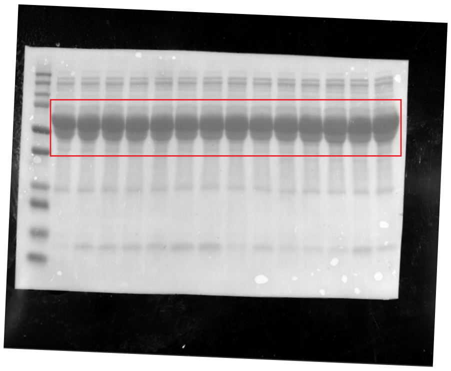

Figure 7—figure supplement 1L

Cyt *c*

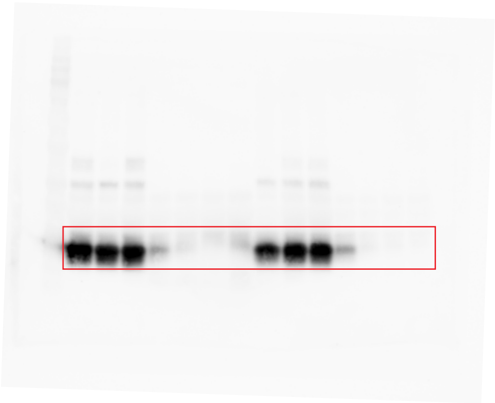

Ponceau S

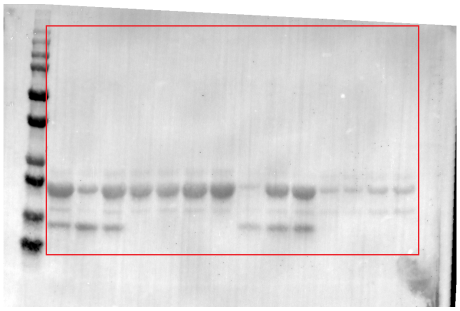

Figure 7—figure supplement 1N

Cyt *c*

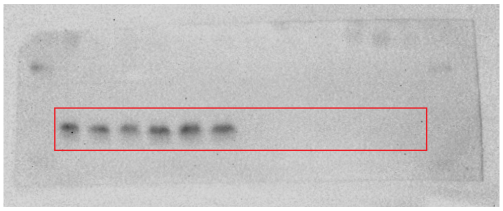

LRG1

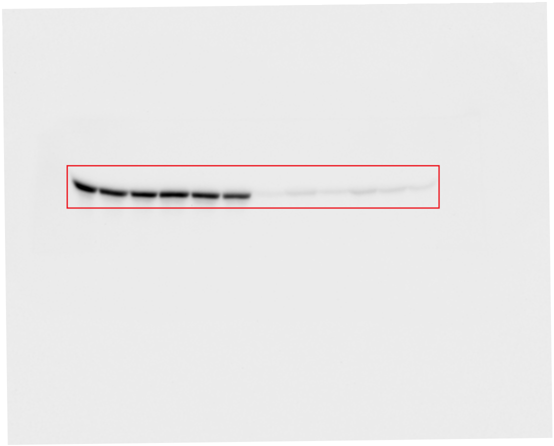

Ponceau S

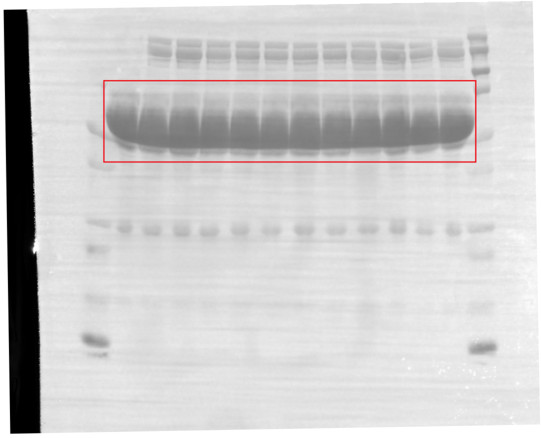

Supplement: Figure 7—source data 1. [file elife-81559-fig7-data1.pdf]
