# Supplementary material for: LRG1 is an adipokine that promotes insulin sensitivity and suppresses inflammation
Source: eLife. 2022 Nov 8;11:e81559. doi: 10.7554/eLife.81559 (PMC9674348; doi:10.7554/eLife.81559)
Supplement: Supplementary file 1. [file elife-81559-supp1.docx]

**Supplementary file 1. Proteins detected in adipocyte CM with enrichment score above 80%.**

|  |  | **% Enrichment** | | |
| --- | --- | --- | --- | --- |
|  |  | **Combined** | **eWAT** | **BAT** |
| 1 | *Retn* | 94.2 | 94.2 | 94.2 |
| 2 | *Fabp4* | 94.2 | 91.9 | 94.2 |
| 3 | *Adipoq* | 88.4 | 88.4 | 89.5 |
| 4 | *Cfd* | 87.2 | 87.2 | 87.2 |
| 5 | *Gpd1* | 87.2 | 69.8 | 95.3 |
| 6 | *Lrg1* | 83.7 | 83.7 | 83.7 |
| 7 | *Ghr* | 81.4 | 80.2 | 82.6 |
| 8 | *Hp* | 81.4 | 82.6 | 80.2 |
| 9 | *Rbp4* | 81.4 | 79.1 | 87.2 |
| 10 | *Amy1* | 81.4 | 79.1 | 87.2 |
| 11 | *Angptl4* | 81.4 | 70.9 | 82.6 |
| 12 | *Ccdc80* | 80.2 | 80.2 | 80.2 |
| 13 | *Sod3* | 80.2 | 74.4 | 86.0 |
| 14 | *H2-Q10* | 80.2 | 0.0 | 89.5 |
| 15 | *Lama4* | 75.6 | 66.3 | 80.2 |
| 16 | *Pla1a* | 75.6 | 81.4 | 70.9 |
| 17 | *Agt* | 74.4 | 86.0 | 69.8 |
| 18 | *Cp* | 68.6 | 91.9 | 65.1 |
| 19 | *Me1* | 61.6 | 43.0 | 82.6 |
| 20 | *Tkt* | 19.8 | 2.3 | 86.0 |
| 21 | *Ace* | 15.1 | 87.2 | 0.0 |
| 22 | *Kng1* | 0.0 | 0.0 | 89.5 |
| 23 | *Pcsk5* | 0.0 | 95.3 | 0.0 |
