# Supplementary material for: LRG1 is an adipokine that promotes insulin sensitivity and suppresses inflammation
Source: eLife. 2022 Nov 8;11:e81559. doi: 10.7554/eLife.81559 (PMC9674348; doi:10.7554/eLife.81559)
Supplement: Supplementary file 2. [file elife-81559-supp2.docx]

**Supplementary file 2. Comparison of MS-based adipose secretome studies**

|  | This work | Ali Khan et al. | Deshmukh et al. |
| --- | --- | --- | --- |
| Organism | Mouse | Mouse | Human |
| Type of adipocytes | Visc, SubQ, and Brown | White (SubQ) and Brown | White (SubQ) and Brown |
| Conditions | Basal | Basal and NE stimulation | Basal and NE stimulation |
| Method | FBS-supplemented medium with AHA pulse | FBS-supplemented medium with AHA pulse | Serum-free medium |
| MS Quantification | Label-free | SILAC | Label-free |
| Total # of proteins detected | 604 | 1337 | 1866 |
| Criteria used for secretion prediction/validation | SignalP, PredGPI, SecretomeP, DeepLoc, and GOCC annotation | SignalP and Uniprot annotation | Signal peptide and Uniprot/GOCC annotation |
| # of proteins containing SP or annotated as EC | 472 | 453 | 471 |
| Leptin | Not detected | Not detected | Detected in SubQ |

|  | This work | | Ali Khan et al. | | Deshmukh et al. | |
| --- | --- | --- | --- | --- | --- | --- |
| Batokines | **Detected** | **Enrichment** | **Detected** | **Enrichment** | **Detected** | **Enrichment** |
| BMP8B | Yes | Found in SubQ 1/3 | - |  | - |  |
| SLIT2 | Yes | Cluster 3  (SubQ and Brown) | Yes |  | - |  |
| IL6 | - |  | Yes |  | - |  |
| VEGFA | Yes | Cluster 4 (Brown) | Yes |  | Yes | Brown-exclusive |
| CXCL14 | - |  | - |  | - |  |
| GDF15 | Yes | Cluster 4 (Brown) | Yes |  | - |  |
| NGF | Yes | Cluster 4 (Brown) | Yes | Brown-enriched | - |  |
| S100B | - |  | - |  | Yes |  |
| NRG4 | - |  | - |  | - |  |
| MSTN | - |  | - |  | - |  |
| FGF21 | Yes | SubQ- and Brown-exclusive | Yes |  | - |  |
| EPDR1 | Yes | Found in Brown 1/3 | - |  | Yes | Brown-exclusive |

|  | This work | | Ali Khan et al. | | Deshmukh et al. | |
| --- | --- | --- | --- | --- | --- | --- |
| Secreted factor | **Detected?** | **Enrichment** | **Detected?** | **Enrichment** | **Detected?** | **Enrichment** |
| CFH | Yes | Cluster 1 (Visc) | Yes | SubQ-enriched | Yes | Brown-enriched |
| CKMT1 | - |  | - |  | Yes | Brown-enriched |
| COL18A1 | Yes | Cluster 2 (SubQ) | - |  | Yes | SubQ-exclusive |
| TNC | Yes |  | Yes |  | Yes | SubQ-enriched |
| MUP2 | Yes | Cluster 2 (SubQ) | Yes | SubQ-enriched | - |  |
| LYZ2 | Yes | Cluster 1 (Visc) | Yes | Brown-enriched | - |  |
| SPON1 | Yes | Cluster 2 (SubQ) | Yes | SubQ-enriched | Yes |  |
| SPARCL1 | Yes | Cluster 1 (Visc) | Yes | SubQ-enriched | Yes |  |
| CTSS | Yes | Cluster 1 (Visc) | Yes | Brown-enriched | - |  |
| ECM proteins | Cluster 2 (SubQ)-enriched | | Higher in Brown | | Higher in SubQ | |
