# Supplementary material for: LRG1 is an adipokine that promotes insulin sensitivity and suppresses inflammation
Source: eLife. 2022 Nov 8;11:e81559. doi: 10.7554/eLife.81559 (PMC9674348; doi:10.7554/eLife.81559)
Supplement: Supplementary file 3. [file elife-81559-supp3.docx]

**Supplementary file 3. Primer sequences used for RT-qPCR analysis**

| **Gene** | **Forward Primer (5’ to 3’)** | **Reverse Primer (5’ to 3’)** |
| --- | --- | --- |
| *Fabp4* | ACACCGAGATTTCCTTCAAACTG | CCATCTAGGGTTATGATGCTCTTCA |
| *Pparg2* | GCATGGTGCCTTCGCTGA | TGGCATCTCTGTGTCAACCATG |
| *Adipoq* | GCACTGGCAAGTTCTACTGCAA | GTAGGTGAAGAGAACGGCCTTGT |
| *Prdm16* | CAGCACGGTGAAGCCATTC | GCGTGCATCCGCTTGTG |
| *Ucp1* | CTTTGCCTCACTCAGGATTGG | ACTGCCACACCTCCAGTCATT |
| *Lrg1* | ATCAAGGAAGCCTCCAGGATCT | CTCAGCCGACTGCAGTATCA |
| *Il1b* | GCAACTGTTCCTGAACTCAACT | ATCTTTTGGGGTCCGTCAACT |
| *Cxcl10* | GGATCCCTCTCGCAAGGA | ATCGTGGCAATGATCTCAACA |
| *Il6* | TAGTCCTTCCTACCCCAATTTCC | TTGGTCCTTAGCCACTCCTTC |
| *Nos2* | GTTCTCAGCCCAACAATACAAGA | GTGGACGGGTCGATGTCAC |
| *Arg1* | CTCCAAGCCAAAGTCCTTAGAG | AGGAGCTGTCATTAGGGACATC |
| *Clec10a* | TGAGAAAGGCTTTAAGAACTGGG | GACCACCTGTAGTGATGTGGG |
| *Mgl2* | TTAGCCAATGTGCTTAGCTGG | GGCCTCCAATTCTTGAAACCT |
| *Chil3* | CAGGTCTGGCAATTCTTCTGAA | GTCTTGCTCATGTGTGTAAGTGA |
| *Rn18s* | CGATGCTCTTAGCTGAGTGT | GGTCCAAGAATTTCACCTCT |
